# Long-term exposure to multiple air pollutants and multi-level socioeconomic status: joint effects on age-related macular degeneration, subsequent ocular comorbidity, and death in middle-aged and older adults

**DOI:** 10.1186/s12916-025-04224-6

**Published:** 2025-07-01

**Authors:** Yang Yang, Liangkai Chen, Qichen Liu, Min Mu, Jing Huang, Guangming Zhang, Qianqian Song

**Affiliations:** 1 Department of Nutrition and Food Hygiene, Hubei Key Laboratory of Food Nutrition and Safety, School of Public Health, Tongji Medical College, Huazhong University of Science and Technology, Wuhan, 430074 China; 2 School of Public Health, Imperial College London, London, SW7 2AZ UK; 3 Ministry of Education Key Lab of Environment and Health, School of Public Health, Tongji Medical College, Huazhong University of Science and Technology, Wuhan, 430074 China; 4 Department of Epidemiology and Biostatistics, School of Public Health, Peking University, Beijing, 10010 China; 5 Beijing Center for Disease Prevention and Control, Institute for Environmental Health, Beijing, 10010 China; 6 School of Public Health, Anhui University of Science and Technology, Hefei, 231131 China; 7 Department of Occupational and Environmental Health Sciences, School of Public Health, Peking University, Haidian District, 38 Xueyuan Road, Beijing, 100191 China; 8 School of Biomedical Informatics, The University of Texas Health Science Center at Houston, Houston, TX 77030 USA; 9 Department of Health Outcomes and Biomedical Informatics, University of Florida, Gainesville, FL 32611 USA

**Keywords:** Air pollution, Socioeconomic status, Age-related macular degeneration, Ocular comorbidity, Mortality

## Abstract

**Background:**

Both air pollution and socioeconomic status (SES) are recognized as significant determinants of health outcomes. However, no study has explored the combined effects of air pollutants and SES on (1) age-related macular degeneration (AMD) incidence; (2) trajectories from baseline to AMD, subsequent ocular comorbidity (OCMD), and mortality; and (3) life expectancy in middle-aged and older adults.

**Methods:**

Using UK Biobank data, we created two composite air pollution scores (APS) and assessed SES at individual and neighborhood levels. OCMD was defined as glaucoma or cataract occurrence after AMD diagnosis. Cox proportional hazard regression models, multistate models, and life tables were used to assess associations and calculate life expectancy.

**Results:**

Over a median of 12.5 years, 3859 participants developed AMD, 2907 participants developed OCMD, and 23,363 died. Compared to those with low APS and favorable SES, individuals with high APS and unfavorable SES had highest risk (APS1: individual-level SES HR 1.41, 95% CI: 1.18–1.67, area-level SES HR 1.31, 95% CI: 1.15–1.49; APS2: individual-level SES HR 1.51, 95% CI: 1.27–1.80; area-level SES HR 1.31, 95% CI: 1.15–1.49), after adjusting for all potential covariates. Among five transitions, the combined effects were significant in transitions from baseline to incident AMD, from AMD to OCMD, and from baseline to death. Significant life expectancy disparities were observed; individuals with low individual-level SES had shortest life expectancies across APS tertiles, with similar but less pronounced effects for area-level SES.

**Conclusions:**

Our study underscores the need for interventions addressing air pollution and SES to reduce AMD risk, improve ocular health, and enhance life expectancy in aging populations.

**Supplementary Information:**

The online version contains supplementary material available at 10.1186/s12916-025-04224-6.

## Background

Age-related macular degeneration (AMD) is a leading cause of irreversible vision loss among middle-aged and older adults [1]. Approximately 170 million people are affected globally, accounting for 8.7% of reported blindness, with numbers projected to reach 288 million by 2040 [2]. As the population ages, AMD prevalence will increase, necessitating deeper understanding of its etiological factors. While contributors such as age, lifestyle, and genetics are well-studied [1, 3], environmental risk factors remain under-explored.

Ambient air pollution warrants particular attention given its ubiquitous presence and well-established links to increased morbidity and mortality worldwide [4]. Emerging evidence has identified ambient air pollution as a potential risk factor for AMD [5–7]. The association likely operates through oxidative stress and inflammation, to which the retina is particularly vulnerable as one of the highest oxygen-consuming tissues, making it susceptible to cumulative oxidative damage that leads to dysfunction and cell loss with aging [8–10].

Socioeconomic status (SES) may be an important factor influencing AMD risk at both individual and area levels. Air pollution-related health effects vary across SES backgrounds, aligning with the triple jeopardy hypothesis: individuals of low SES and those residing in deprived areas face (1) greater exposure to air pollutants and (2) increased susceptibility to adverse health outcomes due to psychosocial stressors, limited health-promoting choices, and restricted healthcare access, ultimately leading to (3) health inequalities [11–14]. Understanding the interplay between air pollutants and SES is vital for identifying at-risk populations and informing targeted public health interventions aimed at reducing AMD incidence and its complications.

However, prior research has limitations. First, most studies examining individual air pollutants fail to capture complex pollutant interactions [15]. As pollutants often share sources and correlate strongly, single-pollutant analyses may not reflect real-world exposure to pollutant mixtures [16]. Second, previous studies have typically addressed a single trajectory of AMD progression, such as the transition from healthy to AMD, or AMD to death, limiting a comprehensive understanding of how air pollution and SES affect AMD’s dynamic progression. Examining the complete and natural disease progression from baseline to AMD incidence, subsequent ocular comorbidities (OCMD), and ultimately to mortality is particularly important because AMD frequently co-occurs with other age-related eye diseases, including glaucoma and cataracts, which are highly prevalent among the elderly. While AMD, glaucoma, and cataracts are distinct clinical entities with unique genetic factors, emerging evidence suggests they may share certain age-related biological processes, including cellular senescence and oxidative stress [17, 18], autophagy dysfunction [19, 20], and chronic inflammation [21], all of which may contribute to their co-occurrence patterns. Furthermore, growing evidence suggests AMD may serve as an indicator of underlying systemic processes that influence longevity, with epidemiological studies demonstrating associations between AMD and increased mortality risk [22, 23]. Understanding this interconnected disease progression is essential for comprehensively evaluating how environmental and socioeconomic factors influence the complete trajectory of AMD and its sequelae. Third, many studies are constrained by cross-sectional design, restricting the ability to evaluate temporal relationships, thereby reducing the strength to infer causality [5–7].

Therefore, this study aims to address these gaps by investigating the combined effects of long-term exposure to multiple air pollutants and multi-level SES on (1) incident AMD; (2) trajectories from baseline to incident AMD, subsequent OCMD, and death; and (3) life expectancy in middle-aged and older adults.

## Methods

### Study population

The UK Biobank represented a large prospective cohort study, enrolling over half a million participants aged between 37 and 73 years during 2006–2010 throughout the UK. This study gathered extensive information both at the initial enrollment and during subsequent follow-up periods. At one of 22 assessment centers, participants engaged in both touchscreen and nurse-conducted questionnaires, underwent a series of physical measurements, and provided biological samples. The methodology and protocols of the UK Biobank have been thoroughly outlined elsewhere [24]. The UK Biobank study was granted under the approval of the North West Multicenter Research Ethical Committee (REC reference 21/NW/0157). All participants provided written informed consent. This research utilized the UK Biobank Resource under Application Number 63424 and 88,159.

In this study, we excluded participants younger than 45 years at baseline (*N* = 51,773) and those with incomplete data on air pollutants (*N* = 36,828) or SES (*N* = 68,724). Additionally, 23,029 participants with prevalent AMD, glaucoma, or cataract at baseline and 1274 participants due to incorrect temporal sequencing were excluded (specifically participants whose glaucoma or cataract occurred concurrently with or prior to AMD onset), resulting in a final sample size of 320,565 participants for the main analysis. Details of the selection process were presented in Additional file 1: Fig. S1.

### Air pollution assessment

Annual average air pollution concentration of the year 2010 was used to represent long-term exposure [25, 26]. Annual air pollutant estimates were derived using a Land Use Regression (LUR) model from the ESCAPE project (http://www.escapeproject.eu), which utilized GIS-derived predictors like land use, traffic, and topography at a 100 m × 100 m resolution. Air pollution concentrations were assigned to participants based on residential coordinates. Detailed information about the model is available elsewhere, with leave-one-out cross-validation indicating good performance [27, 28]. Additionally, similar distribution patterns were observed when comparing the air pollution exposures in the UK Biobank with the spatial distributions of air pollutants from the public UK Air Information Resources (https://uk-air.defra.gov.uk/data/pcm-data) [26, 29].

To assess joint exposure to multiple ambient air pollutants, two methods were used to calculate air pollution score (APS) [26, 30]. First, principal component analysis (PCA) with varimax rotation was performed to calculate APS, which ranged from − 3.75 to 21.14. Second, we developed a weighted APS through adding concentrations of the five air pollutants, weighted by the multivariable-adjusted risk estimates (*β* coefficients) on incident AMD in this analysis. The equation was as follows: APS = (*β*[PM_2.5_] * PM_2.5_ + *β*[PM_10_] * PM_10_ + *β*[PM_2.5–10_] * PM_2.5–10_ + *β*[NO_2_] * NO_2_ + *β*[NO_x_] * NO_x_) / (5 / sum of the *β* coefficients). The APS varied between 41.97 and 118.32, as a higher score indicating higher exposure to ambient air pollution. Correlation analysis of air pollutants and factor loadings from PCA are presented in Additional file 1: Tables S1 and S2.

### Socioeconomic status assessment

We calculated individual-level SES (ISES) by using latent class analysis, incorporating educational background, household income, and employment status. Three latent classes were identified, which respectively represented a high, medium, and low ISES according to the item-response probabilities. Townsend deprivation index (TDI) was utilized as a measure of area-level SES (ASES), which was derived from national census data according to postcodes of residence, incorporating car ownership, household overcrowding, owner occupation, and unemployment [31]. Additionally, we replaced the TDI with the Index of Multiple Deprivation (IMD) as the indicator of ASES, which was published by UK government to classify relative deprivation. Details are provided in Additional file 1: Supplement Method 1.

### Polygenic risk score

The genotyping process and quality control for the UK Biobank have been documented elsewhere [32]. The polygenic risk score (PRS) for AMD was obtained from the UK Biobank PRS Release on the UK Biobank’s Research Access Platform. The PRS was generated using Bayesian analysis based on meta-analyses of summary statistics from external GWASs (standard PRS). The PRS calculation involved multiplying the genome-wide sum of the per-variant posterior effect size by allele dosage. Detailed information regarding the methods can be found at https://biobank.ndph.ox.ac.uk/showcase/refer.cgi?id=5202 and https://www.medrxiv.org/content/10.1101/2022.06.16.22276246v2. The AMD-PRS was further categorized into tertiles, representing low, medium, and high genetic risk levels.

### Covariates

Age, sex, ethnicity, region, smoking status, frequency of alcohol intake, and sleep duration were collected through touchscreen questionnaire. Body mass index (BMI) was calculated through weight divided by the square of height (kg/m^2^). The Metabolic Equivalent Task (MET) minutes, derived from items in the short International Physical Activity Questionnaire (IPAQ), were used to categorize physical activity as “high,” “moderate,” or “low” [33]. We defined a healthy diet score based on dietary priorities for cardiometabolic disease [34], where a higher score indicated healthier dietary habits. Definitions of each component of a healthy diet score were described in Additional file 1: Table S3. Environmental exposure-related covariates including 24-h weighted average noise, proximity to major roads, greenspace percentage within a 300-m buffer of home location, and length of time at current address were also included.

### Ascertainment of outcome and life expectancy

The key outcomes included incident AMD, OCMD, and death. OCMD was defined as the occurrence of either glaucoma or cataract after an AMD diagnosis. All outcomes were identified through linkage sources from primary care, hospital inpatient, and death registry records. Follow-up time was calculated from the date of assessment center attended until the date of death, the date of losing follow-up, or the end of study (30 September 2021 for England and Wales and 31 October 2021 for Scotland). Details for disease coded were shown in Additional file 1: Table S4.

To calculate life expectancy, we used life tables from age 50 to 100 years [35, 36], incorporating: (i) sex- and age-specific population mortality rates from the Office for National Statistics (https://www.ons.gov.uk/peoplepopulationandcommunity/birthsdeathsandmarriages/lifeexpectancies/datasets/singleyearlifetablesuk1980to2018/singleyearlifetablesuk); (ii) sex-specific adjusted hazard ratios for all-cause mortality across APS and SES interactions; and (iii) sex-specific prevalence of each interactive level. Detailed methodology is provided in Additional file 1: Supplement Method 2.

### Statistical analysis

Descriptive characteristics were presented as frequencies with percentages for categorical variables and means with standard deviations (SD) for continuous variables. Spearman’s correlation coefficients were computed to present the pairwise associations among the air pollutants. Missing data of covariates were imputed with multiple imputations by chained equations approach with five imputed datasets and ten iterations [37]. Each of the five imputed datasets was analyzed independently and then the results were aggregated with Rubin’s rule to account for the uncertainty.

Cox proportional hazard regression models were employed to assess the joint effects of APS and SES on AMD. The proportional hazards assumption was verified through statistical tests and graphical diagnostics based on scaled Schoenfeld residuals, revealing no violations. We performed two models. Model 1 adjusted for age at recruitment, sex, region, body mass index, ethnicity, smoking status, alcohol assumption, physical activity, healthy diet score, sleep duration, noise, greenspace, inverse distance to nearest major road, and length of time at current address. Model 2 further adjusted for AMD-PRS, genotyping array, and the first ten genetic principal components. Interactions were evaluated using likelihood ratio tests to compare models with and without a cross-product term. Collinearity between covariates was scrutinized through a correlation matrix analysis and the calculation of the variance inflation factor, revealing no multicollinearity issues. Additionally, both additive and multiplicative interactions between APS and SES regarding incident AMD were analyzed.

We used multistate models to examine the associations of APS and SES with the trajectory from baseline to AMD, subsequent OCMD, and death. The multistate model is a probabilistic model that incorporates multiple states enabling modeling of the rate of transitions between states [38, 39]. It extends the traditional Cox proportional hazards model by simultaneously investigating the influence of risk factors on different stages of disease progression, while accounting for competing risks [40]. Five transitions were constructed as (1) baseline to AMD, (2) baseline to death, (3) AMD to OCMD, (4) AMD to death, (5) and OCMD to death. For participants entering different stages on the same date, we calculated the entering date of theoretically prior state as the date of the latter state minus 0.5 day based on previous study [25, 41].

In the supplement analysis, we investigated independent effects of APS and SES on AMD, OCMD, and all-cause mortality, respectively. The concentration–response associations between APS and each outcome were examined using restricted cubic splines (RCS) with 4 knots at the 5th, 35th, 65th, and 95th percentiles. Moreover, we investigated the combined effects of APS with AMD-PRS, as well as the combined effects of APS with healthy lifestyles on the risk of AMD.

Several sensitivity analyses were conducted to test the robustness of the findings. For multistate analysis, we: (1) further adjusted for prevalent hypertension, diabetes, cardiovascular disease, asthma, chronic obstructive pulmonary disease, depression, and anxiety, (2) further adjusted for solid fuel use and passive smoking, (3) mutually adjusted for individual-level or area-level SES, (4) treated missing values as a distinct category labeled “missing,” (5) excluded participants experiencing events or deaths in the first 2 years to mitigate the potential issue of reverse causality, and (6) excluded participants who experienced different states on the same day. For incident AMD analysis, we replaced the TDI with the IMD as the area-level SES indicator and compared their joint effects with APS on AMD.

All statistical analyses were conducted using R software version 4.3.1. We used Monte Carlo simulation (parametric bootstrapping) with 10,000 runs to calculate the CIs of the life expectancy estimation with boot R package. All statistical tests were two sided, and we considered *P* value of less than 0.05 to be statistically significant.

## Results

### Baseline and exposure characteristics

The baseline characteristics of the included participants stratified by incident AMD were presented in Table 1. Of the 320,565 participants, the mean (SD) age at recruitment was 57.6 (6.8) years, and 167,665 (52.3%) were females. During a median follow-up of 12.5 years, 3859 (1.2%) participants experienced AMD, 2907 (0.9%) participants experienced OCMD, and 23,363 (7.3%) experienced all-cause mortality. The baseline characteristics of the included participants, stratified by tertiles of APS1 and APS2, are presented in Additional file 1: Tables S5 and S6.

**Table 1:** Baseline characteristics of the participants stratified by incident AMD

	**Overall**	**AMD**	**Non-AMD**	***P* value**
**Number**	320,565	3859	316,706	
**Age at recruitment, mean (SD)**	57.6 (6.8)	62.6 (5.3)	57.5 (6.8)	< 0.001
**Sex, *n* (%)**				
Female	167,665 (52.3)	2307 (59.8)	165,358 (52.2)	< 0.001
Male	152,900 (47.7)	1552 (40.2)	151,348 (47.8)	
**Body mass index (BMI), kg/m**^**2**^**, *n* (%)**				
18.5–24.9	102,609 (32.0)	1125 (29.2)	101,484 (32.0)	< 0.001
< 18.5	1549 (0.5)	15 (0.4)	1534 (0.5)	
25.0–29.9	138,494 (43.2)	1705 (44.2)	136,789 (43.2)	
≥ 30.0	77,913 (24.3)	1014 (26.3)	76,899 (24.3)	
**Ethnicity, *n* (%)**				
White	306,959 (95.8)	3721 (96.4)	303,238 (95.7)	0.042
Other	13,606 (4.2)	138 (3.6)	13,468 (4.3)	
**Region, *n* (%)**				
Urban	296,389 (92.5)	3606 (93.4)	292,783 (92.4)	0.021
Rural	24,176 (7.5)	253 (6.6)	23,923 (7.6)	
**Household income, £/year, *n* (%)**				
Greater than 100,000	16,487 (5.1)	75 (1.9)	16,412 (5.2)	< 0.001
52,000 to 100,000	62,949 (19.6)	467 (12.1)	62,482 (19.7)	
31,000 to 51,999	82,804 (25.8)	789 (20.4)	82,015 (25.9)	
18,000 to 30,999	84,129 (26.2)	1196 (31.0)	82,933 (26.2)	
Less than 18,000	74,196 (23.1)	1332 (34.5)	72,864 (23.0)	
**Education, *n* (%)**				
College or university degree	108,054 (33.7)	1046 (27.1)	107,008 (33.8)	< 0.001
Professional qualification	90,323 (28.2)	1028 (26.6)	89,295 (28.2)	
Secondary school	71,563 (22.3)	870 (22.5)	70,693 (22.3)	
Primary school	50,625 (15.8)	915 (23.7)	49,710 (15.7)	
**Employment, *n* (%)**				
Active	182,887 (57.1)	1289 (33.4)	181,598 (57.3)	< 0.001
Inactive	137,678 (42.9)	2570 (66.6)	135,108 (42.7)	
**Individual-level SES, *n* (%)**				
High	98,014 (30.6)	647 (16.8)	97,367 (30.7)	< 0.001
Medium	150,049 (46.8)	1793 (46.5)	148,256 (46.8)	
Low	72,502 (22.6)	1419 (36.8)	71,083 (22.4)	
**Area-level SES, *n* (%)**				
Least deprived	106,857 (33.3)	1254 (32.5)	105,603 (33.3)	0.323
Moderately deprived	106,854 (33.3)	1328 (34.4)	105,526 (33.3)	
Severely deprived	106,854 (33.3)	1277 (33.1)	105,577 (33.3)	
**Smoking status, *n* (%)**				
Never	172,607 (53.8)	1886 (48.9)	170,721 (53.9)	< 0.001
Previous	115,879 (36.1)	1624 (42.1)	114,255 (36.1)	
Current	32,079 (10.0)	349 (9.0)	31,730 (10.0)	
**Alcohol drinking frequency, *n* (%)**				
≥ 3 times/week	147,906 (46.1)	1725 (44.7)	146,181 (46.2)	< 0.001
< 3 times/week	149,902 (46.8)	1774 (46.0)	148,128 (46.8)	
Never	22,757 (7.1)	360 (9.3)	22,397 (7.1)	
**Sleep duration, *n* (%)**				
7–8 h/day	218,176 (68.1)	2557 (66.3)	215,619 (68.1)	< 0.001
< 7 h/day	78,689 (24.5)	951 (24.6)	77,738 (24.5)	
> 8 h/day	23,700 (7.4)	351 (9.1)	23,349 (7.4)	
**Physical activity, *n* (%)**				
High	128,154 (40.0)	1544 (40.0)	126,610 (40.0)	0.999
Moderate	131,275 (41.0)	1579 (40.9)	129,696 (41.0)	
Low	61,136 (19.1)	736 (19.1)	60,400 (19.1)	
**Healthy diet score, *n* (%)**				
0–2	118,913 (37.1)	1279 (33.1)	117,634 (37.1)	< 0.001
3–5	190,161 (59.3)	2424 (62.8)	187,737 (59.3)	
≥ 6	11,491 (3.6)	156 (4.0)	11,335 (3.6)	
**AMD-PRS, *n* (%)**				
Low	106,856 (33.3)	918 (23.8)	105,938 (33.4)	
Medium	106,854 (33.3)	1080 (28.0)	105,774 (33.4)	
High	106,855 (33.3)	1861 (48.2)	104,994 (33.2)	
** Noise, mean (SD)**	56.0 (4.3)	55.9 (4.2)	56.0 (4.3)	0.197
** Inverse distance to nearest major road, mean (SD)**	0.01 (0.02)	0.01 (0.01)	0.01 (0.02)	0.216
**Greenspace, *n* (%)**				
First quartile	80,152 (25.0)	893 (23.1)	79,259 (25.0)	0.001
Second quartile	80,131 (25.0)	974 (25.2)	79,157 (25.0)	
Third quartile	80,145 (25.0)	1064 (27.6)	79,081 (25.0)	
Fourth quartile	80,137 (25.0)	928 (24.0)	79,209 (25.0)	
**Length of time at current address, *n* (%)**				
≤ 10 years	106,488 (33.2)	1120 (29.0)	105,368 (33.3)	< 0.001
10–20 years	84,644 (26.4)	803 (20.8)	83,841 (26.5)	
> 20 years	129,433 (40.4)	1936 (50.2)	127,497 (40.3)	
** NO**_**2**_**, mean (SD)**	26.38 (7.57)	26.43 (7.28)	26.38 (7.58)	0.708
** NO**_**x**_**, mean (SD)**	43.55 (15.46)	43.63 (14.55)	43.55 (15.47)	0.769
** PM**_**10**_**, mean (SD)**	16.20 (1.90)	16.26 (1.89)	16.2 (1.90)	0.041
** PM**_**2.5**_**, mean (SD)**	9.96 (1.05)	10.01 (1.02)	9.96 (1.06)	0.009
** PM**_**2.5–10**_**, mean (SD)**	6.42 (0.90)	6.44 (0.92)	6.42 (0.90)	0.163
**APS1, *n* (%)**				
First tertile	106,856 (33.3)	1211 (31.4)	105,645 (33.4)	0.021
Second tertile	106,854 (33.3)	1350 (35.0)	105,504 (33.3)	
Third tertile	106,855 (33.3)	1298 (33.6)	105,557 (33.3)	
**APS2, *n* (%)**				
First tertile	106,855 (33.3)	1207 (31.3)	105,648 (33.4)	0.023
Second tertile	106,856 (33.3)	1336 (34.6)	105,520 (33.3)	
Third tertile	106,854 (33.3)	1316 (34.1)	105,538 (33.3)	
**Death, *n* (%)**				0.743
No	297,202 (92.7%)	293,630 (92.7%)	3572 (92.6%)	
Yes	23,363 (7.3%)	23,076 (7.3%)	287 (7.4%)	

*SES*, socioeconomic status; *APS1*, air pollution score calculated by PCA; *APS2*, air pollution score calculated by weighted coefficients; *NO2*, nitrogen dioxide; *NOx*, nitrogen oxides; *PM2.5*, particular matter with aerodynamic diameter ≤ 2.5 mm; *PM10*, particular matter with an aerodynamic diameter ≤ 10 mm; *PM2.5–10*, particular matter with aerodynamic diameter 2.5–10 mm; *AMD*, age-related macular degeneration; *PRS*, polygenic risk score

Health diet score was calculated based on self-reported servings of fruits, vegetables, whole grains, vegetable oil, fish, dairy, refined grains, unprocessed meats, processed meats, and sugar-sweetened beverages

Individual-level SES was constructed by employment, education, and household income and calculated by latent class analysis

Townsend index (including measures of unemployment, non-car ownership, non-home ownership, and household overcrowding), derived from respondents’ postcode, was used as an indicator of area-level SES

### Cox regression results

**Fig. 1:**
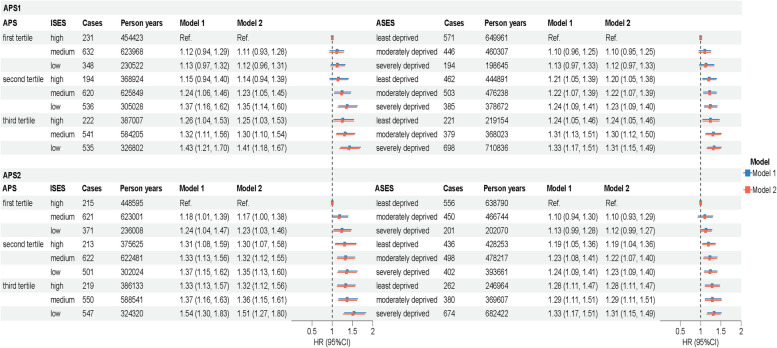
Joint associations of air pollutants and socioeconomic status with incident AMD. CI, confidence interval; HR, hazard ratio; APS1, air pollution score calculated by PCA; APS2, air pollution score calculated by weighted coefficients; ISES, individual-level socioeconomic status; ASES, area-level socioeconomic status; AMD, age-related macular degeneration. Model 1 adjusted for age at recruitment, sex, region, body mass index, ethnicity, smoking status, alcohol assumption, physical activity, healthy diet score, sleep duration, noise, green space, inverse distance to nearest major road, and length of time at current address. Model 2 further adjusted for AMD genetic risk score, genotyping array, and the first 10 principal components of ancestry

The results of joint analyses involving APS and SES in relation to incident AMD are presented in Fig. 1, which revealed significant interaction effects (all *P* for interaction < 0.001). Compared to individuals in the low tertile of APS with favorable SES, those in the top tertile of APS with unfavorable SES exhibited the greatest risk of AMD, even after adjusting for all potential covariates (APS1: ISES, HR 1.41, 95% CI: 1.18–1.67; ASES, HR 1.31, 95% CI: 1.15–1.49; APS2: ISES, HR 1.51, 95% CI: 1.27–1.80; ASES, HR 1.31, 95% CI: 1.15–1.49). Dose–response associations between air pollutants and each outcome are presented in Additional file 1: Figs. S2–S4. Joint associations of APS and SES with OCMD and all-cause mortality are shown in Additional file 1: Figs. S5–S8.

### Multistate analyses

**Fig. 2:**
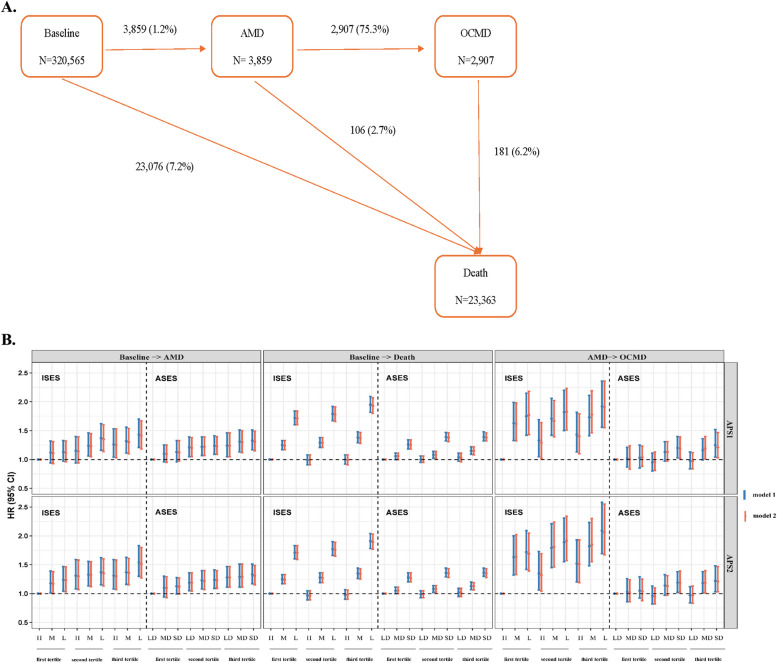
Associations of air pollutants and socioeconomic status with transitions from baseline to AMD, subsequent OCMD, and death*. *Transitions from AMD to death and from OCMD to death were not presented due to the small sample size, which resulted in no statistical significance. CI, confidence interval; HR, hazard ratio; APS1, air pollution score calculated by PCA; APS2, air pollution score calculated by weighted coefficients; ISES, individual-level socioeconomic status; ASES, area-level socioeconomic status; AMD, age-related macular degeneration; OCMD, ocular comorbidity; H, high; M, medium; L, low; LD, least deprived; MD, moderately deprived; SD, severely deprived. Model 1 adjusted for age at recruitment, sex, region, body mass index, ethnicity, smoking status, alcohol assumption, physical activity, healthy diet score, sleep duration, noise, green space, inverse distance to nearest major road, and length of time at current address. Model 2 further adjusted for AMD genetic risk score, genotyping array, and the first 10 principal components of ancestry

Numbers (percentages) of participants in transitioning from baseline to AMD, subsequent OCMD, and death are presented in Fig. 2A. Among five transitions, the combined effects of APS and SES were consistently associated with an increased risk of transitions from baseline to AMD, from baseline to death, and from AMD to OCMD (Fig. 2B). Less pronounced effects were observed for the combination of APS and area-level SES compared to the combination of APS and individual-level SES.

### Estimated life expectancy

**Fig. 3:**
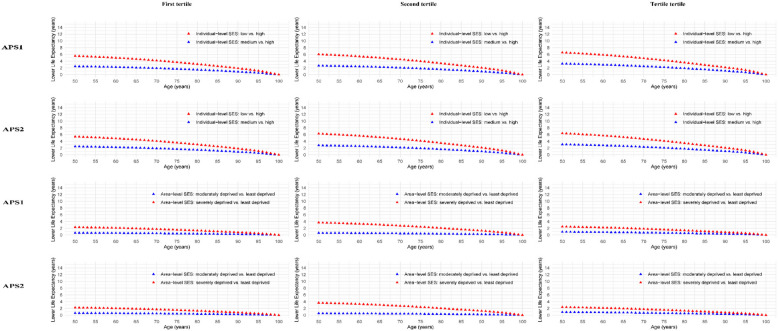
Differences in estimated life expectancy from 50 years of age onward in men by socioeconomic status across air pollution score. For the analysis of life expectancy, air pollution scores were categorized into three tertiles. Within each tertile, the reduction in life expectancy was compared between individuals with medium and low SES versus those with high SES at the individual level. For area-level SES, life expectancy reductions were compared between areas classified as moderately deprived and severely deprived versus those classified as least deprived. Model was adjusted for age at recruitment, sex, region, body mass index, ethnicity, smoking status, alcohol assumption, physical activity, healthy diet score, sleep duration, noise, green space, inverse distance to nearest major road, and length of time at current address. APS1, air pollution score calculated by PCA; APS2, air pollution score calculated by weighted coefficients; SES, socioeconomic status

Figure 3 shows life expectancy differences from age 50 in men by SES across APS tertiles. Life expectancy progressively decreased from favorable to unfavorable SES consistently across APS1 and APS2 tertiles. At age 50, compared to those with high ISES, males with low ISES lost 5.5 (95% CI: 4.8–6.3) years for first tertile, 6.0 (95% CI: 5.3–6.8) years for second tertile, 6.6 (95% CI:5.9–7.3) years for third tertile in APS1, and 5.4 (95% CI: 4.7–6.1) years for first tertile, 6.3 (95% CI: 5.4–7.1) years for second tertile, 6.4 (95% CI: 5.7–7.1) years for third tertile in APS2, respectively. Less pronounced reductions in ASES showed decreases of 2.3 (95% CI: 1.8–2.8), 3.7 (95% CI: 3.1–4.3), and 2.5 (95% CI: 2.0–3.0) years for APS1, and 2.2 (95% CI: 1.7–2.7), 3.6 (95% CI: 3.1–4.3), and 2.4 (95% CI: 1.9–2.9) years for APS2, respectively.

**Fig. 4:**
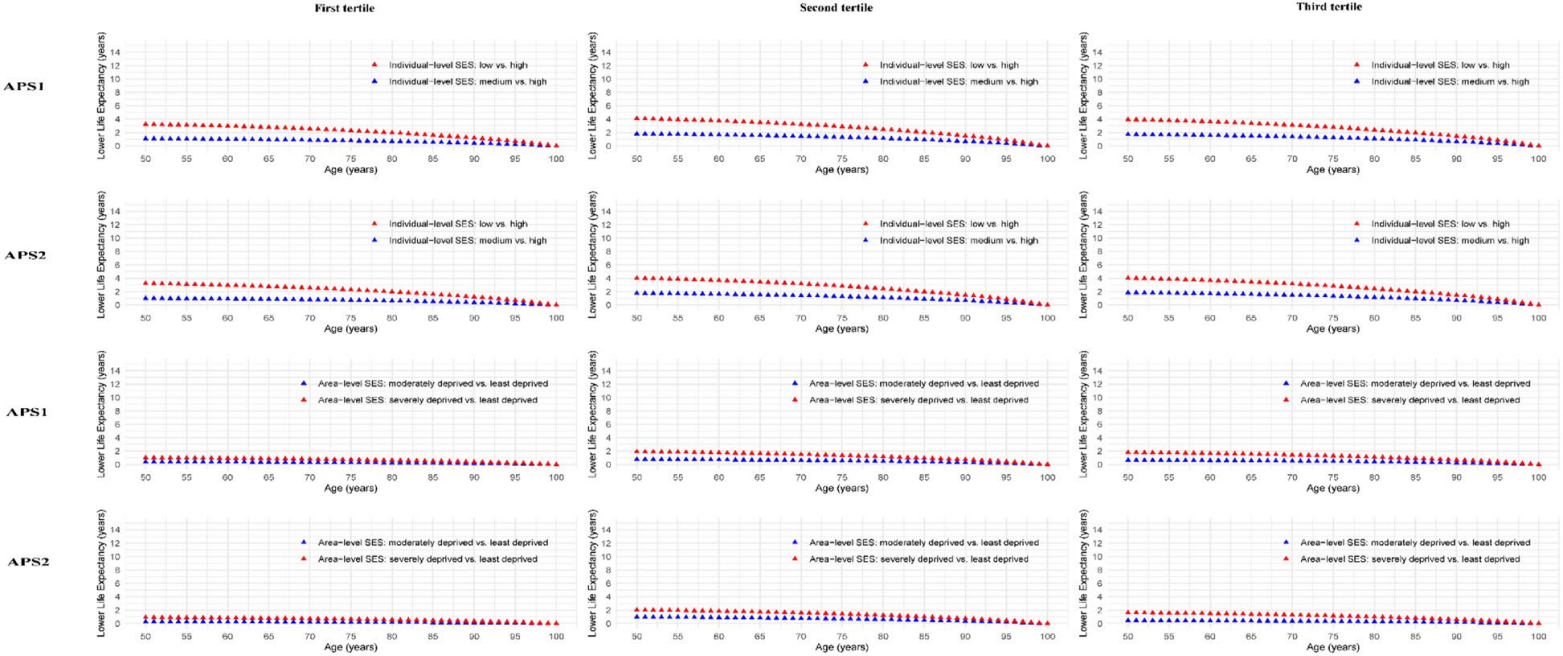
Differences in estimated life expectancy from 50 years of age onward in women by socioeconomic status across air pollution score. For the analysis of life expectancy, air pollution scores were categorized into three tertiles. Within each tertile, the reduction in life expectancy was compared between individuals with medium and low SES versus those with high SES at the individual level. For area-level SES, life expectancy reductions were compared between areas classified as moderately deprived and severely deprived versus those classified as least deprived. Model was adjusted for age at recruitment, sex, region, body mass index, ethnicity, smoking status, alcohol assumption, physical activity, healthy diet score, sleep duration, noise, green space, inverse distance to nearest major road, and length of time at current address. APS1, air pollution score calculated by PCA; APS2, air pollution score calculated by weighted coefficients; SES, socioeconomic status

Accordingly, women showed similar patterns (Fig. 4). At age 50, for ISES, life expectancy reduced 3.2 (95% CI: 2.3–4.2), 4.1 (95% CI: 3.2–5.1), and 3.9 (95% CI: 3.0–4.9) years across APS1 tertiles, and 3.2 (95% CI: 2.3–4.1), 4.0 (95% CI: 3.1–5.0), and 4.0 (95% CI: 3.1–4.9) years across APS2 tertiles, respectively. ASES reductions were smaller: 1.0 (95% CI: 0.5–1.4), 1.9 (95% CI: 1.3–2.4), and 1.8 (95% CI: 1.3–2.3) years for APS1, and 0.9 (95% CI: 0.4–1.3), 2.0 (95% CI: 1.4–2.5), and 1.6 (95% CI: 1.1–2.2) years for APS2.

### Supplement and sensitivity analyses

Similar results were observed when comparing TDI with IMD on AMD risk (Additional file 1: Figs. S9 and S10). Significant interactions were observed between APS and AMD-PRS or healthy behaviors on AMD (Additional file 1: Figs. S11 and S12). Both APS and SES were independently associated with increased risk of AMD, OCMD, and mortality (Additional file 1: Tables S7–S12). The main findings remained robust across multiple sensitivity analyses, including additional adjustment for baseline prevalent diseases, additional adjustment for solid fuel use and passive smoking, mutual adjustment for individual- or area-level SES, using a distinct category for missing covariates, excluding events within first 2 years of follow-up, and excluding same-day events (Additional file 1: Tables S13–S24). Analyses on additive and multiplicative interactions between APS and SES with incident AMD are presented in Additional file 1: Tables S25 and S26.

## Discussion

In this large-scale prospective cohort study, we observed notable combined effects of APS and SES on incidence of AMD, even after controlling for demographic, lifestyle, genetic, and environmental factors. Among five transitions, the combined effects were significant in transitions from baseline to AMD, from AMD to OCMD, and from baseline to death. Significant disparities in life expectancy were also observed: individuals with low ISES had the shortest life expectancies across APS tertiles, with similar but less pronounced patterns for ASES. These findings are vital for guiding health policies and interventions to mitigate air pollution, reduce socioeconomic disparities, and improve life expectancy in aging population.

Previous studies have identified air pollutants and disadvantaged SES as significant risk factors for AMD [5–7, 42, 43]. However, most research focused on individual air pollutants and assessed SES in a limited manner, overlooking the complexity of real-world exposures and the multifaceted nature of SES. Specifically, multiple air pollutants, often highly correlated and simultaneously present in ambient air, may interact synergistically, amplifying adverse health effects in ways that single-pollutant analyses fail to capture [15, 16]. Additionally, the collinearity between correlated pollutants presents a challenge in single-pollutant regressions, making it difficult to isolate independent effects [44]. Similarly, SES is a complex, multidimensional concept which cannot be fully reflected by a single socioeconomic domain (e.g., education, occupation, income) [45]. It can be conceptualized as individual- or area-level, both of which were independently related to air pollution and health outcome [46, 47]. In this study, we developed composite air pollution scores and multi-level SES measures to address these limitations. And our findings are consistent with those of a recent study that employed a similar APS methodology to assess the effects of air pollution on AMD [48]. Such efforts provide basic evidence for addressing the complexity of environmental and social determinants of ocular health, paving the way for more nuanced analyses for at-risk populations.

Although it has been widely recognized that AMD may be influenced by exposure to air pollution, no studies have identified and quantified the risk of transition from incident AMD to subsequent OCMD in aging population. Our study has provided evidence that long-term exposure to air pollution may accelerate the development of OCMD among AMD patients, and unfavorable SES further exacerbated these risks, which filled a critical gap in understanding the disease trajectory. Regretfully, analyzing the transition from AMD to death and from OCMD to death was constrained because the relatively low occurrence of mortality events. We call for future studies to fully elucidate the impact of air pollution and SES on the entire disease trajectory.

Potential mechanisms have been proposed to explain how the combined effects of APS and multi-level SES influence AMD development, subsequent OCMD, and mortality. Air pollution increases oxidative stress and inflammation, processes to which the retina is particularly vulnerable due to its high oxygen consumption and susceptibility to oxidative damage [8–10]. This damage accumulates with age, leading to retinal dysfunction and cell loss, thus accelerating the transition from healthy state to incident AMD [8–10]. The progression of AMD may be further exacerbated by both individual- and area-level SES disadvantages. At the individual level, lower income, education, and occupational status are associated with higher levels of personal stress and inflammatory responses, while at the area level, residing in socioeconomically deprived neighborhoods exposes individuals to higher levels of air pollution and environmental toxins [46, 49, 50]. Such prolonged exposure increases the risk of ophthalmic conditions including glaucoma and cataracts [51], while the resulting chronic inflammation and oxidative burden lead to cardiovascular and respiratory disorders, worsening overall health and increasing mortality risk. Limited access to healthcare services among those with lower individual SES and those living in deprived areas results in delayed diagnosis and suboptimal management, leading to faster disease progression and development of ophthalmic comorbidities, which, coupled with delayed treatment of other chronic conditions, further increases mortality risk. Additionally, both individual-level (through limited financial resources and health literacy) and area-level (through poor neighborhood infrastructure) SES disadvantages lead to unhealthy lifestyles [52–54], which synergistically interact with air pollution exposure to accelerate AMD progression and increase the likelihood of developing ocular complications. These behavioral factors, combined with inadequate healthcare access, environmental exposures, and chronic stress, create a cycle of deteriorating ocular health and overall health status, ultimately leading to increased mortality risk.

To our knowledge, this study was the first to explore the combined effects of air pollutants and SES on life expectancy. Our analysis highlighted health inequalities linked to SES disparities, showing a graded association between SES and life expectancy, suggesting that even minor improvements in socioeconomic conditions could lead to significant enhancements in life expectancy. Notably, these patterns were consistent across APS tertiles. This finding stresses the necessity for interventions that not only tackle the direct impacts of air pollution but also address the socioeconomic vulnerabilities that exacerbate these effects. We found that both individual- and area-level SES played significant roles in determining health outcomes, underscoring the need for public health strategies that address not only personal economic hardships but also enhance the quality of community environments. Improving individual and community factors can more effectively reduce health disparities and promote longer life expectancy among vulnerable populations. Additionally, our study showed that even in the UK—where concentrations of various air pollutants are relatively low—relying solely on single-pollutant models may underestimate the total health risks associated with air pollution. Therefore, greater emphasis should be placed on evaluating the health effects of multipollutant exposure, and the integration of APS into future air quality standards and regulatory frameworks should be strongly considered.

Our findings carry important implications for clinical and policy-level decision-making. For clinicians, awareness of the cumulative risk posed by air pollution and socioeconomic disadvantage may prompt earlier screening for AMD and ocular comorbidities among high-risk patients. For policymakers, integrating composite exposure metrics such as APS into air quality surveillance and population health frameworks may improve risk assessment and resource allocation. Tailored public health strategies that address both environmental exposures and social determinants could contribute to more equitable and effective prevention of visual impairment in aging populations. Taken together, these implications demonstrate how epidemiological research can inform both prevention strategies and resource prioritization in real-world settings.

Some limitations should also be noted. First, the study relied solely on baseline air pollution data from the UK Biobank, with the annual average concentrations for 2010 representing long-term exposure to air pollution. However, air pollution emissions remained relatively stable from 2010 to 2019 in the UK, and there is evidence indicating that using annual mean air pollutants at baseline showed similar associations with that using time-varying air pollutants [55–57]. Second, although we controlled for potential confounders based on prior evidence, there may have been some residual and unobserved confounding. Third, the air pollution score did not include all air pollutants, such as ozone and ultrafine particles, which might also be related to the risk of total mortality. Fourth, data on occupational exposure were not available, and information regarding personal time-activity patterns, which result in individuals spending time in various microenvironments with different exposures, was also lacking. Although none of these factors was captured in our analysis, our study focused on outdoor concentrations. Fifth, as this study relied exclusively on medical records to define AMD events, cases of early or asymptomatic AMD that did not lead to a clinical diagnosis may have been missed, potentially leading to an underestimation of the true disease burden. In addition, we were unable to distinguish between different stages (early, intermediate, late) or subtypes (dry vs. neovascular) of AMD due to data availability. Given that the risks of cataract and glaucoma comorbidities may vary across AMD stages and subtypes, our findings should be interpreted with caution. Future research with detailed phenotypic information is warranted to clarify these potential differences. Sixth, although we cannot entirely rule out the possibility of undiagnosed ocular comorbidities prior to AMD diagnosis, participants with documented comorbidities before AMD were excluded from our trajectory analysis. In addition, the UK Biobank provides regularly updated medical records, which helps minimize the risk of missing long-standing ocular diseases. Nevertheless, potential misclassification of disease onset is an inherent limitation of trajectory analyses using electronic health records and warrants cautious interpretation of our findings. Finally, generalizing the results to other populations should be done cautiously, as the majority of participants in the UK Biobank were of European descent.

## Conclusions

In conclusion, the study demonstrated the significant interactions between air pollution and SES on incident AMD, OCMD, and all-cause mortality. These combined effects were associated with increased risks of progression trajectories from baseline to AMD, baseline to death, and AMD to OCMD. Higher air pollutant exposure with disadvantaged SES significantly shorter life expectancy in middle-aged and older adults. It highlighted the urgent need for targeted public health interventions that address both environmental and socioeconomic factors to promote healthy aging in vulnerable populations.

## Supplementary Information

Additional file 1: Supplement Method 1 Assessment of socioeconomic status. Supplement Method 2 Statistical method used for estimating the life expectancy. Table S1 Correlation analysis of air pollutants. Table S2 Factor loadings from principal components analysis to create APS1. Table S3 Definition of each component of a healthy diet score. Table S4 Ascertainment of outcome. Table S5 Baseline characteristics of the participants by APS1. Table S6 Baseline characteristics of the participants by APS2. Table S7 Hazard ratios and 95% confidence interval for the air pollution scores with incident AMD. Table S8 Hazard ratios and 95% confidence interval for the air pollution scores with incident OCMD after AMD diagnosis. Table S9 Hazard ratios and 95% confidence interval for the air pollution scores with all-cause mortality. Table S10 Hazard ratios and 95% confidence interval for the socioeconomic status with incident AMD. Table S11 Hazard ratios and 95% confidence interval for the socioeconomic status with incident OCMD after AMD diagnosis. Table S12 Hazard ratios and 95% confidence interval for the socioeconomic status with all-cause mortality. Table S13 Associations of APS1 and socioeconomic status with transitions. Table S14 Associations of APS2 and socioeconomic status with transitions. Table S15 Associations of APS1 and socioeconomic status with transitions. Table S16 Associations of APS2 and socioeconomic status with transitions. Table S17 Associations of APS1 and socioeconomic status with transitions. Table S18 Associations of APS2 and socioeconomic status with transitions. Table S19 Associations of APS1 and socioeconomic status with transitions. Table S20 Associations of APS2 and socioeconomic status with transitions. Table S21 Associations of APS1 and socioeconomic status with transitions. Table S22 Associations of APS2 and socioeconomic status with transitions. Table S23 Associations of APS1 and socioeconomic status with transitions. Table S24 Associations of APS2 and socioeconomic status with transitions. Table S25 Analyses on additive and multiplicative interactions between APS1 and SES with incident AMD. Table S26 Analyses on additive and multiplicative interactions between APS2 and SES with incident AMD. Fig. S1 Flowchart of participant enrollment. Fig. S2 Dose–response associations between air pollutants and incident AMD. Fig. S3 Dose–response associations between air pollutants and incident OCMD after AMD diagnosis. Fig. S4 Dose–response associations between air pollutants and all-cause mortality. Fig. S5 Joint associations of APS1 and socioeconomic status with incident OCMD after AMD diagnosis. Fig. S6 Joint associations of APS2 and socioeconomic status with incident OCMD after AMD diagnosis. Fig. S7 Joint associations of APS1 and socioeconomic status with all-cause mortality. Fig. S8 Joint associations of APS2 and socioeconomic status with all-cause mortality. Fig. S9 Joint associations of APS1 and area-level SESwith AMD. Fig. S10 Joint associations of APS2 and area-level SESwith AMD. Fig. S11 Joint associations of air pollutants and AMD-PRS with incident AMD. Fig. S12 Joint associations of air pollutants and healthy lifestyle with incident AMD.

## Data Availability

The data of this study can be requested from the UK Biobank (https://www.ukbiobank.ac.uk/).

## Abbreviations

SES: Socioeconomic status
ISES: Individual-level SES
ASES: Area-level SES
AMD: Age-related macular degeneration
OCMD: Ocular comorbidity
APS: Air pollution scores
PCA: Principal component analysis
HR: Hazard ratio
CI: Confidence interval
SD: Standard deviations
RCS: Restricted cubic splines
LUR: Land Use Regression
TDI: Townsend deprivation index
IMD: Index of Multiple Deprivation
PRS: Polygenic risk score
BMI: Body mass index
MET: Metabolic Equivalent Task
IPAQ: International Physical Activity Questionnaire
