# Supplementary material for: Long-term exposure to multiple air pollutants and multi-level socioeconomic status: joint effects on age-related macular degeneration, subsequent ocular comorbidity, and death in middle-aged and older adults
Source: BMC Med. 2025 Jul 1;23:354. doi: 10.1186/s12916-025-04224-6 (PMC12211924; doi:10.1186/s12916-025-04224-6)
Supplement: Supplementary file 1 — Additional file 1: Supplement Method 1 Assessment of socioeconomic status. Supplement Method 2 Statistical method used for estimating the life expectancy. Table S1 Correlation analysis of air pollutants. Table S2 Factor loadings from principal components analysis to create APS1. Table S3 Definition of each component of a healthy diet score. Table S4 Ascertainment of outcome. Table S5 Baseline characteristics of the participants by APS1. Table S6 Baseline characteristics of the participants by APS2. Table S7 Hazard ratios and 95% confidence interval for the air pollution scores with incident AMD. Table S8 Hazard ratios and 95% confidence interval for the air pollution scores with incident OCMD after AMD diagnosis. Table S9 Hazard ratios and 95% confidence interval for the air pollution scores with all-cause mortality. Table S10 Hazard ratios and 95% confidence interval for the socioeconomic status with incident AMD. Table S11 Hazard ratios and 95% confidence interval for the socioeconomic status with incident OCMD after AMD diagnosis. Table S12 Hazard ratios and 95% confidence interval for the socioeconomic status with all-cause mortality. Table S13 Associations of APS1 and socioeconomic status with transitions. Table S14 Associations of APS2 and socioeconomic status with transitions. Table S15 Associations of APS1 and socioeconomic status with transitions. Table S16 Associations of APS2 and socioeconomic status with transitions. Table S17 Associations of APS1 and socioeconomic status with transitions. Table S18 Associations of APS2 and socioeconomic status with transitions. Table S19 Associations of APS1 and socioeconomic status with transitions. Table S20 Associations of APS2 and socioeconomic status with transitions. Table S21 Associations of APS1 and socioeconomic status with transitions. Table S22 Associations of APS2 and socioeconomic status with transitions. Table S23 Associations of APS1 and socioeconomic status with transitions. Table S24 Associations of APS [file 12916_2025_4224_MOESM1_ESM.docx]

**Long-term exposure to multiple air pollutants and multi-level socioeconomic status: joint effects on age-related macular degeneration, subsequent ocular comorbidity and death in middle-aged and older adults**

Additional file 1

**Content**

Supplement Method 1. Assessment of socioeconomic status

Supplement Method 2. Statistical method used for estimating the life expectancy

Table S1. Correlation analysis of air pollutants

Table S2. Factor loadings from principal components analysis to create APS1

Table S3. Definition of each component of a healthy diet score

Table S4. Ascertainment of outcome

Table S5. Baseline characteristics of the participants by APS1

Table S6. Baseline characteristics of the participants by APS2

Table S7. Hazard ratios and 95% confidence interval for the air pollution scores with incident AMD

Table S8. Hazard ratios and 95% confidence interval for the air pollution scores with incident OCMD after AMD diagnosis

Table S9. Hazard ratios and 95% confidence interval for the air pollution scores with all-cause mortality

Table S10. Hazard ratios and 95% confidence interval for the socioeconomic status with incident AMD

Table S11. Hazard ratios and 95% confidence interval for the socioeconomic status with incident OCMD after AMD diagnosis

Table S12. Hazard ratios and 95% confidence interval for the socioeconomic status with all-cause mortality

Table S13. Associations of APS1 and socioeconomic status with transitions (further adjusted for prevalent diseases)

Table S14. Associations of APS2 and socioeconomic status with transitions (further adjusted for prevalent diseases)

Table S15. Associations of APS1 and socioeconomic status with transitions (further adjusted for solid fuel use and passive smoking)

Table S16. Associations of APS2 and socioeconomic status with transitions (further adjusted for solid fuel use and passive smoking)

Table S17. Associations of APS1 and socioeconomic status with transitions (mutually adjusted for SES)

Table S18. Associations of APS2 and socioeconomic status with transitions (mutually adjusted for SES)

Table S19. Associations of APS1 and socioeconomic status with transitions (missing values as a level)

Table S20. Associations of APS2 and socioeconomic status with transitions (missing values as a level)

Table S21. Associations of APS1 and socioeconomic status with transitions (remove cases and deaths within the first 2 years, N=318860)

Table S22. Associations of APS2 and socioeconomic status with transitions (remove cases and deaths within the first 2 years, N=318860)

Table S23. Associations of APS1 and socioeconomic status with transitions (remove participants who experienced different states on the same day, N=320311)

Table S24. Associations of APS2 and socioeconomic status with transitions (remove participants who experienced different states on the same day, N=320311)

Table S25. Analyses on additive and multiplicative interactions between APS1 and SES with incident AMD

Table S26. Analyses on additive and multiplicative interactions between APS2 and SES with incident AMD

Fig. S1. Flowchart of participant enrolment

Fig. S2. Dose-response associations between air pollutants and incident AMD

Fig. S3. Dose-response associations between air pollutants and incident OCMD after AMD diagnosis

Fig. S4. Dose-response associations between air pollutants and all-cause mortality

Fig. S5. Joint associations of APS1 and socioeconomic status with incident OCMD after AMD diagnosis

Fig. S6. Joint associations of APS2 and socioeconomic status with incident OCMD after AMD diagnosis

Fig. S7. Joint associations of APS1 and socioeconomic status with all-cause mortality

Fig. S8. Joint associations of APS2 and socioeconomic status with all-cause mortality

Fig. S9. Joint associations of APS1 and area-level SES (TDI vs. IMD) with AMD

Fig. S10. Joint associations of APS2 and area-level SES (TDI vs. IMD) with AMD

Fig. S11. Joint associations of air pollutants and AMD-PRS with incident AMD

Fig. S12. Joint associations of air pollutants and healthy lifestyle with incident AMD

**Supplement Method 1. Assessment of socioeconomic status**

In this study, we utilized two socioeconomic indices: area-level and individual-level socioeconomic status (SES) parameters. Latent class analysis based on three indicators (household income, education level, and employment status) was used to create individual-level SES parameter [1,2].

**Household income**

Participants reported their average total before tax household income as 'less than £18,000', '£18,000 to £30,999', '£31,000 to £51,999', '£52,000 to £100,000', 'greater than £100,000', 'do not know' and 'prefer not to answer'. Participants who selected the last two options were excluded from the main analysis.

**Education**

Participants reported their education level as 'college or university degree'; 'A levels/AS levels or equivalent'; 'O levels/GCSEs or equivalent'; 'CSEs or equivalent'; 'NVQ, HND, HNC, or equivalent'; 'other professional qualifications (for example, nursing, teaching)'; 'none of the above'; or 'prefer not to answer'. As this was a multiple-choice question, participants could select more than one option. We decided to assign each participant's highest level of education as their education level.

**Table for Supplement Method 2. Each level in UK education system** [3,4]

| **Level** | **Type of education** | **Description** |
| --- | --- | --- |
| college or university degree | academic education | This corresponds to Level 6 or above in the UK education framework, which includes bachelor's degrees, master's degrees, and doctorates. These are considered the highest levels of academic achievement. |
| A levels/AS levels or equivalent | academic education | A Levels are Level 3 qualifications, typically taken at the end of secondary school, and are essential for university entrance. |
| O levels/GCSEs or equivalent | academic education | O Levels are Level 2 qualifications, which have been largely replaced by GCSEs (General Certificate of Secondary Education). High grades in GCSEs are essential for progression to A Levels or vocational qualifications. |
| CSEs or equivalent | academic education | Certificate of Secondary Education (CSEs) were typically Level 1 qualifications, equivalent to lower grades in GCSEs. |
| NVQ, HND, HNC, or equivalent | vocational education | NVQ (National Vocational Qualifications) can vary widely in level. HNC (Higher National Certificate) is a Level 4 qualification, and HND (Higher National Diploma) is a Level 5 qualification. These are vocational qualifications that are highly regarded and often equivalent to the early years of university degrees. |
| professional qualifications (for example, nursing, teaching) | vocational education | These qualifications can vary, with nursing and teaching typically requiring an undergraduate degree. |
| none of the above | -- | equivalent to primary school [2] |
| The existing variable 'qualifications' (field id: 6138) was applied. If one selected "college or university degree," they were classified as such. If not, and they chose "NVQ or HND or HNC or equivalent/Other professional qualifications eg: nursing, teaching", they fell under "professional qualification." If neither applied but picked "A levels/AS levels or equivalent/O levels/GCSEs or equivalent/CSEs or equivalent", they were labelled "secondary school." If none of these options but selected "None of the above," they were categorized as "primary school." For "NA" or "prefer not to answer," the designation was "NA." | | |

Therefore, we classified the education levels into four ordinal categories: 'college or university degree', 'professional qualification' (including 'NVQ, HND, HNC, or equivalent', and 'other professional qualifications'), 'secondary school' (including 'A levels/AS levels or equivalent', 'O levels/GCSEs or equivalent', 'CSEs or equivalent'), and 'primary school'.

**Employment**

Participants reported their employment status as 'in paid employment or self-employed', 'retired', 'looking after home and/or family', 'unable to work because of sickness or disability', 'unemployed', 'doing unpaid or voluntary work', 'full or part-time student', 'none of the above', and 'prefer not to answer'. As this was a multiple-choice question, participants could select more than one option.

**Table for Supplement Method 2. Assignments for employment status for participants**

| **variable** | **assignments** |
| --- | --- |
| employment status | We defined employment based on 'Current employment status' (field id: 6142), and 'Current employment status-corrected' (field id: 20119). We reclassified participants into two categories, reflecting a focus on distinguishing active workforce engagement. If selected "retired," they were categorized as "retired." If not, and they chose "in paid employment or self-employed/doing unpaid or voluntary work", they were classified as "active." If none of the prior options were chosen but one of "looking after home and/or family, unable to work because of sickness or disability, unemployed, full or part-time student, none of the above" was selected, they were categorized as "inactive." For "NA" or "prefer not to answer," the designation was "NA." |

Therefore, employment status was classified into two categories: 'active' (comprising those in paid employment, self-employed, or doing unpaid or voluntary work) and 'inactive' (encompassing those who chose 'looking after home and/or family', 'unable to work because of sickness or disability', 'unemployed', 'retired', 'full or part-time student', or 'none of the above').

**Individual-level SES**

We used "household income", "education", and "employment" to create an individual-level SES parameter using latent class analysis. The method has been described in previous study [1,2]. Different numbers of latent classes were conducted and Akaike information criterion (AIC) and Bayesian information criterion (BIC) were used for the model selection. The mean posterior probability, which reflected the uncertainty of posterior classification, was also used for the model selection, and a value of 0.7 or more indicated an acceptable uncertainty. Item-response probability was a posterior probability and was used for defining latent classes.

Since BICs were similar with classes from three to five (failed to converge for 6 classes), interpretability and practicality should also be considered [5,6]. Three-latent class solution were identified and mean posterior probabilities of all latent classes were above 0.8.

**Table for Supplement Method 2.**  **Item-response probabilities in models with three latent classes**

| Item | Latent class 1 | Latent class 2 | Latent class 3 |
| --- | --- | --- | --- |
| college or university degree | 0.592 | 0.125 | 0.283 |
| professional qualification | 0.236 | 0.229 | 0.338 |
| secondary school | 0.152 | 0.226 | 0.267 |
| primary school | 0.020 | 0.420 | 0.112 |
| active | 0.841 | 0.179 | 0.597 |
| inactive | 0.159 | 0.821 | 0.403 |
| greater than 100000 | 0.171 | 0.002 | <0.001 |
| 52000 to 100000 | 0.578 | <0.001 | 0.054 |
| 31000 to 51999 | 0.251 | <0.001 | 0.395 |
| 18000 to 30999 | <0.001 | 0.220 | 0.451 |
| less than 18000 | <0.001 | 0.778 | 0.100 |
| prevalence of latent classes | 0.305 | 0.227 | 0.468 |
| mean posterior probabilities | 0.831 | 0.834 | 0.806 |

Latent class analysis was used to create individual-level SES. Latent class 1 was regarded as 'high' in individual-level SES; latent class 2 was categorized as 'low' and latent class 3 as 'medium'. SES, socioeconomic status

Latent class 1 was characterized by high-level average household income, high education qualification, and active workforce engagement, which could be defined as "high SES" ; latent class 2 was characterized by low-level average household income, low education qualification, and inactive workforce engagement, which could be defined as "low SES" ; latent class 3 was characterized by medium-level average household income, medium education qualification, and active and inactive workforce engagement, which could be defined as "medium SES".

**Area-level SES**

Townsend deprivation index was utilized as a measure of area-level SES, which was derived from national census data according to postcodes of residence, incorporating car ownership, household overcrowding, owner occupation, and unemployment [7]. We categorised Townsend deprivation index into tertiles, with the lowest score indicating the least socioeconomic deprivation.

**Sensitivity analysis**

Regarding area-level SES, we replaced the Townsend deprivation index with the Index of Multiple Deprivation (IMD) as the indicator of area-level SES. IMD scores were used to classify the relative deprivation (a measure of poverty) in British local councils published by UK government (https://www.gov.uk/government/collections/english-indices-of-deprivation). IMD scores were calculated separately in England (EIMD), Scotland (SIMD) and Wales (WIMD) because of multiple different components. The EIMD score consists of seven domain indices, including: income deprivation (income subdomain, income deprivation affecting children index and older people index); employment deprivation; health deprivation and disability; education score; barriers to housing and services (wider and geographical barriers subdomain); living environment deprivation (indoors and outdoors subdomain); and crime score [8]. The SIMD score consisted of seven domain indices, including: crime (only from 2006), current income, education, skills and training, employment, geographical access, health and housing [8]. The WIMD score is composed of eight domain indices for income, employment, health, education, access to services, community safety, physical environment and housing [8]. The categorization of IMD into tertiles was applied for each area, with the lowest score indicating the least socioeconomic deprivation.

**Reference**

1. Li R, Li R, Xie J, Chen J, Liu S, Pan A, Liu G. Associations of socioeconomic status and healthy lifestyle with incident early-onset and late-onset dementia: a prospective cohort study. Lancet Healthy Longev. 2023 Dec;4(12):e693-e702. doi: 10.1016/S2666-7568(23)00211-8. PMID: 38042162.

2. Zhang YB, Chen C, Pan XF, Guo J, Li Y, Franco OH, Liu G, Pan A. Associations of healthy lifestyle and socioeconomic status with mortality and incident cardiovascular disease: two prospective cohort studies. BMJ. 2021 Apr 14;373:n604. doi: 10.1136/bmj.n604. PMID: 33853828; PMCID: PMC8044922.

3.GOV.UK - What Qualification Levels Mean. <https://www.gov.uk/what-different-qualification-levels-mean/list-of-qualification-levels>

4.Office for National Statistics (ONS) - Education, England and Wales. <https://www.ons.gov.uk/peoplepopulationandcommunity/educationandchildcare>

5. Sinha P, Calfee CS, Delucchi KL. Practitioner's Guide to Latent Class Analysis: Methodological Considerations and Common Pitfalls. Crit Care Med. 2021 Jan 1;49(1):e63-e79. doi: 10.1097/CCM.0000000000004710. PMID: 33165028; PMCID: PMC7746621.

6. Nylund, K. L., Asparouhov, T. and Muthén, B. O. (2007) ‘Deciding on the Number of Classes in Latent Class Analysis and Growth Mixture Modeling: A Monte Carlo Simulation Study’, Structural Equation Modeling: A Multidisciplinary Journal, 14(4), pp. 535–569. doi: 10.1080/10705510701575396.

7. Foster HME, Celis-Morales CA, Nicholl BI, Petermann-Rocha F, Pell JP, Gill JMR, O'Donnell CA, Mair FS. The effect of socioeconomic deprivation on the association between an extended measurement of unhealthy lifestyle factors and health outcomes: a prospective analysis of the UK Biobank cohort. Lancet Public Health. 2018 Dec;3(12):e576-e585. doi: 10.1016/S2468-2667(18)30200-7. Epub 2018 Nov 20. PMID: 30467019.

8. Xu J, Liu N, Polemiti E, Garcia-Mondragon L, Tang J, Liu X, Lett T, Yu L, Nöthen MM, Feng J, Yu C, Marquand A, Schumann G; the environMENTAL Consortium. Effects of urban living environments on mental health in adults. Nat Med. 2023 Jun;29(6):1456-1467. doi: 10.1038/s41591-023-02365-w. Epub 2023 Jun 15. PMID: 37322117; PMCID: PMC10287556.

**Supplement Method 2. Statistical method used for estimating the life expectancy**

We combined information from three sources within the same population to estimate lower survival time associated with different levels of interactive combinations of APS and SES (“first tertile” in APS and “high” in individual-level SES as the reference; “first tertile” in APS and “least deprived” in area-level SES as the reference).

(1) Sex- and age- specific population mortality rate from the Office for National Statistics [1];

(2) Sex-specific HRs of all-cause mortality in each exposure group versus the reference in UK biobank;

(2) Sex-specific prevalence of each frequency of different levels in UK biobank.

The sex-specific lifetables for 8 exposure groups in their combinations were built on the above-mentioned three estimates. Population all-cause mortality rates per 100,000 per sex and per single-year age group were obtained from the Office for National Statistics. We used sex-specific Cox regression models to calculate adjusted hazard ratios for all-cause mortality by exposure groups. We made adjustments for age, sex, region, ethnicity, BMI, smoke status, frequency of alcohol intake, healthy diet score, physical activity, sleep duration, noise, proximity to major roads, greenspace percentage, and length of time at current address. Then we applied the sex- specific HRs to estimate the life expectancy at different age of women and men, separately.

We built the life table starting at age 50 years and ending at 100 years by single-year age intervals. Survival probability was set of 1 at age 50 years and probability of survival between ages x and x + 1 was calculated based on probability of dying (mortality rate) between ages x and x+1 assuming that survivor function declines linearly between ages x and x + 1 [2,3]. The life expectancy at any given age was derived by dividing the total person-years that would be lived beyond age x by the number of persons who survived to that age interval [2].

We inferred the age-specific mortality rates appropriate for our reference group 𝐼𝑅_𝑎0_ as [4]:

$$\boldsymbol{IR}_{\boldsymbol{a}\boldsymbol{0}}\boldsymbol{=}\frac{\boldsymbol{IR}_{\boldsymbol{a}}}{\boldsymbol{(}\boldsymbol{P}_{\boldsymbol{a}\boldsymbol{0}}\boldsymbol{+}\sum_{\boldsymbol{j=1}}^{\boldsymbol{n}} \boldsymbol{P}_{\boldsymbol{aj}}\boldsymbol{\times}{\boldsymbol{H}\boldsymbol{R}}_{\boldsymbol{aj}}\boldsymbol{)}}$$

Where 𝐼𝑅_𝑎_ is the population mortality rate for age group 𝑎, *P* _𝑎𝑗_ is the prevalence of exposure group 𝑗, and *H*𝑅_𝑎𝑗_ is the hazard ratio in comparison of exposure group 𝑗 versus reference group (𝑗 = 0). The age-specific mortality rates in each of the non-reference exposure groups were then inferred in turn by multiplying the age-specific mortality rate for the reference group 𝐼𝑅_𝑎0_ by the hazard ratios *H*𝑅_𝑎𝑗_.

Finally, life table was built for each exposure group and the reference group.

**References**

1. Single-year life tables, UK:1980-2020.

Available: [https://www.ons.gov.uk/peoplepopulationandcommunity/birthsdeathsandmarriages/lifeexpectancies/datasets/singloculararlifetablesuk1980to2018/singloculararlifetablesuk](https://www.ons.gov.uk/peoplepopulationandcommunity/birthsdeathsandmarriages/lifeexpectancies/datasets/singleyearlifetablesuk1980to2018/singleyearlifetablesuk) [Accessed 12 Mar 2024].

2. Arias E. United States life tables, 2008. Natl Vital Stat Rep. 2012 Sep 24;61(3):1-63.

3.Chiang CL, World Health Organization. Life table and mortality analysis. 1979. Publisher: Geneva: World Health Organization.

4. Woloshin S, Schwartz LM, Welch HG. The risk of death by age, sex, and smoking status in the United States: putting health risks in context. J Natl Cancer Inst 2008;100(12):845-5

**Table S1. Correlation analysis of air pollutants**

| Items | NO_x_ | PM_10_ | PM_2.5_ | PM_2.5-10_ |
| --- | --- | --- | --- | --- |
| NO_2_ | 0.92* | 0.50* | 0.87* | 0.19* |
| NO_x_ | - | 0.51* | 0.85* | 0.23* |
| PM_10_ | - | - | 0.53* | 0.81* |
| PM_2.5_ | - | - | - | 0.21* |

*P-value< 0.05.

**Table S2. Factor loadings from principal components analysis to create APS1**

| Items | PC1 |
| --- | --- |
| NO_2_ | 0.9048817 |
| NO_x_ | 0.9088218 |
| PM_10_ | 0.7811027 |
| PM_2.5_ | 0.8945513 |
| PM_2.5-10_ | 0.5191564 |

APS: air pollution score. Principal component analysis (PCA) with varimax rotation was performed, and the first PC was regarded as APS.

**Table S3. Definition of each component of a healthy diet score**

| Components | Goal (1 point) | Amount per serving | Field IDs |
| --- | --- | --- | --- |
| fruits | ≥ 3 servings/day | 1 piece of fresh fruit  5 pieces of dried fruit | 1309, 1319 |
| vegetables | ≥ 3 servings/day | 3 heaped tablespoons | 1289, 1299 |
| whole grains | ≥ 3 servings/day | 1 slice of whole-grain bread  1 cup of whole-grain cereal | 1438, 1448, 1458, 1468 |
| vegetable oil | ≥ 2 servings/day | 1 serving/day if in combination with eating at least 2 slices of bread (ID 1438) | 1428 (Flora Pro-Active/Benecol spread), 2654 (Flora Pro-Active/Benecol, soft margarine, olive oil based, polyunsaturated/sunflower oil based, other low/reduced fat spread), 1438 (bread slices/week) |
| (shell) fish | ≥ 2 servings/week | Once/week | 1329, 1339 |
| dairy | ≥ 2 servings/day | 1 glass/day if consumption any type of milk  1 piece of cheese | 1408, 1418 |
| refined grains | ≤ 2 servings/day | 1 slice of bread  1 bowl of cereal | 1438, 1448 (white, brown, other bread slices/week)  1458, 1468 (biscuit, other cereals/week) |
| unprocessed meats | ≤ 2 servings/week | once/week (including poultry, beef, lamb, and pork)  0 pieces/day if indicated having never eaten meat | 1359, 1369, 1379, 1389, 3680 |
| processed meats | ≤ 1 servings/week | 1 piece/day  0 pieces/day if indicated having never eaten meat | 1349, 3680 |
| sugar-sweetened beverages | don’t drink | 0 serving | 6144 |

Data on food consumption were derived from the baseline questionnaire. If participants achieved the intake goal of each diet component, they were considered to have an adequate intake and get one point. The points were then accumulated to calculate the final healthy diet score. A higher score indicated a healthier diet pattern.

**Table S4. Ascertainment of outcome**

|  | **Impatient-ICD10 (41270)** | ***First occurrence-ICD10** | **Impatient-ICD9 (41271)** |
| --- | --- | --- | --- |
| age-related macular degeneration | H353 | - | 3625 |
| glaucoma | H40 | 131186 | 365 |
| cataract | H25; H26; H280-H282 | 131164; 131166 | 74332; 366 |

| ICD: international classification disease. |
| --- |
| * A set of ‘first occurrence’ data-fields have been generated that map the clinical codes from primary care, hospital  inpatient admissions, death records and self-reported medical conditions to 3-character ICD-10 codes and provide,  for each participant, the date that code first occurred in any source. |

**Table S5. Baseline characteristics of the participants by APS1**

|  | Overall | First tertile | Second tertile | Third tertile | P value |
| --- | --- | --- | --- | --- | --- |
| Number | 320565 | 106856 | 106854 | 106855 |  |
| Age at recruitment, mean (SD) | 57.56 (6.80) | 57.97 (6.71) | 57.68 (6.80) | 57.03 (6.87) | <0.001 |
| Sex, n (%) |  |  |  |  | 0.004 |
| female | 167665 (52.3) | 55465 (51.9) | 55981 (52.4) | 56219 (52.6) |  |
| male | 152900 (47.7) | 51391 (48.1) | 50873 (47.6) | 50636 (47.4) |  |
| Body mass index (BMI), kg/m^2^, n (%) |  |  |  |  | <0.001 |
| 18.5-24.9 | 102609 (32.0) | 35443 (33.2) | 33255 (31.1) | 33911 (31.7) |  |
| <18.5 | 1549 (0.5) | 425 (0.4) | 474 (0.4) | 650 (0.6) |  |
| 25.0-29.9 | 138494 (43.2) | 47424 (44.4) | 46124 (43.2) | 44946 (42.1) |  |
| >=30.0 | 77913 (24.3) | 23564 (22.1) | 27001 (25.3) | 27348 (25.6) |  |
| Ethnicity, n (%) |  |  |  |  | <0.001 |
| white | 306959 (95.8) | 105151 (98.4) | 103085 (96.5) | 98723 (92.4) |  |
| other | 13606 (4.2) | 1705 (1.6) | 3769 (3.5) | 8132 (7.6) |  |
| Region, n (%) |  |  |  |  | <0.001 |
| urban | 296389 (92.5) | 85210 (79.7) | 104948 (98.2) | 106231 (99.4) |  |
| rural | 24176 (7.5) | 21646 (20.3) | 1906 (1.8) | 624 (0.6) |  |
| Household income, £/year, n (%) |  |  |  |  | <0.001 |
| greater than 100000 | 16487 (5.1) | 6747 (6.3) | 4233 (4.0) | 5507 (5.2) |  |
| 52000 to 100000 | 62949 (19.6) | 24279 (22.7) | 19774 (18.5) | 18896 (17.7) |  |
| 31000 to 51999 | 82804 (25.8) | 29365 (27.5) | 27970 (26.2) | 25469 (23.8) |  |
| 18000 to 30999 | 84129 (26.2) | 27560 (25.8) | 29085 (27.2) | 27484 (25.7) |  |
| less than 18000 | 74196 (23.1) | 18905 (17.7) | 25792 (24.1) | 29499 (27.6) |  |
| Education, n (%) |  |  |  |  |  |
| college or university degree | 108054 (33.7) | 37648 (35.2) | 32765 (30.7) | 37641 (35.2) | <0.001 |
| professional qualification | 90323 (28.2) | 31693 (29.7) | 31254 (29.2) | 27376 (25.6) |  |
| secondary school | 71563 (22.3) | 23636 (22.1) | 24661 (23.1) | 23266 (21.8) |  |
| primary school | 50625 (15.8) | 13879 (13.0) | 18174 (17.0) | 18572 (17.4) |  |
| Employment, n (%) |  |  |  |  | <0.001 |
| active | 182887 (57.1) | 58692 (54.9) | 60244 (56.4) | 63951 (59.8) |  |
| inactive | 137678 (42.9) | 48164 (45.1) | 46610 (43.6) | 42904 (40.2) |  |
| Individual-level SES, n (%) |  |  |  |  | <0.001 |
| high | 98014 (30.6) | 36595 (34.2) | 29872 (28.0) | 31547 (29.5) |  |
| medium | 150049 (46.8) | 50970 (47.7) | 51271 (48.0) | 47808 (44.7) |  |
| low | 72502 (22.6) | 19291 (18.1) | 25711 (24.1) | 27500 (25.7) |  |
| Area-level SES, n (%) |  |  |  |  | <0.001 |
| least deprived | 106857 (33.3) | 52925 (49.5) | 36255 (33.9) | 17677 (16.5) |  |
| moderately deprived | 106854 (33.3) | 37573 (35.2) | 39108 (36.6) | 30173 (28.2) |  |
| severely deprived | 106854 (33.3) | 16358 (15.3) | 31491 (29.5) | 59005 (55.2) |  |
| Smoking status, n (%) |  |  |  |  | <0.001 |
| never | 172607 (53.8) | 60607 (56.7) | 57904 (54.2) | 54096 (50.6) |  |
| previous | 115879 (36.1) | 38171 (35.7) | 38579 (36.1) | 39129 (36.6) |  |
| current | 32079 (10.0) | 8078 (7.6) | 10371 (9.7) | 13630 (12.8) |  |
| Alcohol drinking frequency, n (%) |  |  |  |  | <0.001 |
| >= 3 times/week | 147906 (46.1) | 53495 (50.1) | 47305 (44.3) | 47106 (44.1) |  |
| < 3 times/week | 149902 (46.8) | 47390 (44.3) | 51952 (48.6) | 50560 (47.3) |  |
| never | 22757 (7.1) | 5971 (5.6) | 7597 (7.1) | 9189 (8.6) |  |
| Sleep duration, n (%) |  |  |  |  | <0.001 |
| 7-8 h/day | 218176 (68.1) | 75236 (70.4) | 72402 (67.8) | 70538 (66.0) |  |
| < 7h/day | 78689 (24.5) | 23773 (22.2) | 26505 (24.8) | 28411 (26.6) |  |
| > 8h/day | 23700 (7.4) | 7847 (7.3) | 7947 (7.4) | 7906 (7.4) |  |
| Physical activity, n (%) |  |  |  |  | <0.001 |
| high | 128154 (40.0) | 43519 (40.7) | 42505 (39.8) | 42130 (39.4) |  |
| moderate | 131275 (41.0) | 43370 (40.6) | 43697 (40.9) | 44208 (41.4) |  |
| low | 61136 (19.1) | 19967 (18.7) | 20652 (19.3) | 20517 (19.2) |  |
| Healthy diet score, n (%) |  |  |  |  | <0.001 |
| 0-2 | 118913 (37.1) | 39190 (36.7) | 40389 (37.8) | 39334 (36.8) |  |
| 3-5 | 190161 (59.3) | 63956 (59.9) | 62870 (58.8) | 63335 (59.3) |  |
| >=6 | 11491 (3.6) | 3710 (3.5) | 3595 (3.4) | 4186 (3.9) |  |
| AMD PRS, n (%) |  |  |  |  | 0.193 |
| low | 106856 (33.3) | 35823 (33.5) | 35678 (33.4) | 35355 (33.1) |  |
| medium | 106854 (33.3) | 35579 (33.3) | 35647 (33.4) | 35628 (33.3) |  |
| high | 106855 (33.3) | 35454 (33.2) | 35529 (33.3) | 35872 (33.6) |  |
| Noise, mean (SD) | 56.0 (4.3) | 55.0 (3.0) | 55.1 (3.0) | 58.0 (5.5) | <0.001 |
| Inverse distance to nearest major road, mean (SD) | 0.01 (0.02) | 0.00 (0.00) | 0.00 (0.01) | 0.01 (0.03) | <0.001 |
| Greenspace, n (%) |  |  |  |  |  |
| first quartile | 80152 (25.0) | 6025 (5.6) | 27569 (25.8) | 46558 (43.6) | <0.001 |
| second quartile | 80131 (25.0) | 12190 (11.4) | 35136 (32.9) | 32805 (30.7) |  |
| third quartile | 80145 (25.0) | 29220 (27.3) | 30710 (28.7) | 20215 (18.9) |  |
| fourth quartile | 80137 (25.0) | 59421 (55.6) | 13439 (12.6) | 7277 (6.8) |  |
| Length of time at current address, n (%) |  |  |  |  | <0.001 |
| <=10 years | 106488 (33.2) | 36754 (34.4) | 33447 (31.3) | 36287 (34.0) |  |
| 10-20 years | 84644 (26.4) | 28651 (26.8) | 27594 (25.8) | 28399 (26.6) |  |
| >20 years | 129433 (40.4) | 41451 (38.8) | 45813 (42.9) | 42169 (39.5) |  |
| NO_2_, mean (SD) | 26.38 (7.57) | 19.26 (3.61) | 26.26 (3.40) | 33.62 (6.66) | <0.001 |
| NO_x_, mean (SD) | 43.55 (15.46) | 30.42 (6.10) | 42.30 (5.60) | 57.94 (16.34) | <0.001 |
| PM_10_, mean (SD) | 16.20 (1.90) | 14.62 (1.29) | 16.20 (0.99) | 17.77 (1.80) | <0.001 |
| PM_2.5_, mean (SD) | 9.96 (1.05) | 8.97 (0.52) | 9.94 (0.46) | 10.98 (0.92) | <0.001 |
| PM_2.5-10_, mean (SD) | 6.42 (0.90) | 6.03 (0.55) | 6.24 (0.69) | 6.99 (1.06) | <0.001 |

SES, socioeconomic status; APS1: air pollution score calculated by PCA; APS2: air pollution score calculated by weighted coefficients; NO_2_, nitrogen dioxide; NO_x_, nitrogen oxides; PM_2.5_, particular matter with aerodynamic diameter ≤2.5mm; PM_10_, particular matter with an aerodynamic diameter ≤10mm; PM_2.5-10_, particular matter with aerodynamic diameter 2.5-10 mm. AMD, age-related macular degeneration; PRS, polygenic risk score. Health diet score was calculated based on self-reported servings of fruits, vegetables, whole grains, vegetable oil, fish, dairy, refined grains, unprocessed meats, processed meats and sugar- sweetened beverages. Individual-level SES was constructed by employment, education and household income and calculated by latent class analysis. Townsend Index (including measures of unemployment, non-car ownership, non- home ownership and household overcrowding), derived from respondents’ postcode was used as an indicator of area- level SES.

**Table S6. Baseline characteristics of the participants by APS2**

|  | Overall | First tertile | Second tertile | Third tertile | P value |
| --- | --- | --- | --- | --- | --- |
| Number | 320565 | 106855 | 106856 | 106854 |  |
| Age at recruitment, mean (SD) | 57.56 (6.80) | 57.94 (6.71) | 57.64 (6.82) | 57.12 (6.85) | <0.001 |
| Sex, n (%) |  |  |  |  | 0.008 |
| female | 167665 (52.3) | 55475 (51.9) | 56055 (52.5) | 56135 (52.5) |  |
| male | 152900 (47.7) | 51380 (48.1) | 50801 (47.5) | 50719 (47.5) |  |
| Body mass index (BMI), kg/m2, n (%) |  | |  |  | <0.001 |
| 18.5-24.9 | 102609 (32.0) | 35206 (32.9) | 33626 (31.5) | 33777 (31.6) |  |
| <18.5 | 1549 (0.5) | 434 (0.4) | 474 (0.4) | 641 (0.6) |  |
| 25.0-29.9 | 138494 (43.2) | 47349 (44.3) | 46055 (43.1) | 45090 (42.2) |  |
| >=30.0 | 77913 (24.3) | 23866 (22.3) | 26701 (25.0) | 27346 (25.6) |  |
| Ethnicity, n (%) |  |  |  |  | <0.001 |
| white | 306959 (95.8) | 104926 (98.2) | 102821 (96.2) | 99212 (92.8) |  |
| other | 13606 (4.2) | 1929 (1.8) | 4035 (3.8) | 7642 (7.2) |  |
| Region, n (%) |  |  |  |  | <0.001 |
| urban | 296389 (92.5) | 87071 (81.5) | 103856 (97.2) | 105462 (98.7) |  |
| rural | 24176 (7.5) | 19784 (18.5) | 3000 (2.8) | 1392 (1.3) |  |
| Household income, £/year, n (%) |  |  |  |  | <0.001 |
| greater than 100000 | 16487 (5.1) | 6575 (6.2) | 4455 (4.2) | 5457 (5.1) |  |
| 52000 to 100000 | 62949 (19.6) | 24023 (22.5) | 19919 (18.6) | 19007 (17.8) |  |
| 31000 to 51999 | 82804 (25.8) | 29210 (27.3) | 27956 (26.2) | 25638 (24.0) |  |
| 18000 to 30999 | 84129 (26.2) | 27613 (25.8) | 28842 (27.0) | 27674 (25.9) |  |
| less than 18000 | 74196 (23.1) | 19434 (18.2) | 25684 (24.0) | 29078 (27.2) |  |
| Education, n (%) |  |  |  |  | <0.001 |
| college or university degree | 108054 (33.7) | 37183 (34.8) | 33675 (31.5) | 37196 (34.8) |  |
| professional qualification | 90323 (28.2) | 31681 (29.6) | 30917 (28.9) | 27725 (25.9) |  |
| secondary school | 71563 (22.3) | 23734 (22.2) | 24450 (22.9) | 23379 (21.9) |  |
| primary school | 50625 (15.8) | 14257 (13.3) | 17814 (16.7) | 18554 (17.4) |  |
| Employment, n (%) |  |  |  |  | <0.001 |
| active | 182887 (57.1) | 58791 (55.0) | 60542 (56.7) | 63554 (59.5) |  |
| inactive | 137678 (42.9) | 48064 (45.0) | 46314 (43.3) | 43300 (40.5) |  |
| Individual-level SES, n (%) |  |  |  |  | <0.001 |
| high | 98014 (30.6) | 36169 (33.8) | 30418 (28.5) | 31427 (29.4) |  |
| medium | 150049 (46.8) | 50911 (47.6) | 51004 (47.7) | 48134 (45.0) |  |
| low | 72502 (22.6) | 19775 (18.5) | 25434 (23.8) | 27293 (25.5) |  |
| Area-level SES, n (%) |  |  |  |  | <0.001 |
| least deprived | 106857 (33.3) | 52027 (48.7) | 34888 (32.6) | 19942 (18.7) |  |
| moderately deprived | 106854 (33.3) | 37317 (34.9) | 39247 (36.7) | 30290 (28.3) |  |
| severely deprived | 106854 (33.3) | 17511 (16.4) | 32721 (30.6) | 56622 (53.0) |  |
| Smoking status, n (%) |  |  |  |  | <0.001 |
| never | 172607 (53.8) | 60427 (56.6) | 57937 (54.2) | 54243 (50.8) |  |
| previous | 115879 (36.1) | 38163 (35.7) | 38489 (36.0) | 39227 (36.7) |  |
| current | 32079 (10.0) | 8265 (7.7) | 10430 (9.8) | 13384 (12.5) |  |
| Alcohol drinking frequency, n (%) |  | |  |  | <0.001 |
| >= 3 times/week | 147906 (46.1) | 53047 (49.6) | 47569 (44.5) | 47290 (44.3) |  |
| < 3 times/week | 149902 (46.8) | 47736 (44.7) | 51609 (48.3) | 50557 (47.3) |  |
| never | 22757 (7.1) | 6072 (5.7) | 7678 (7.2) | 9007 (8.4) |  |
| Sleep duration, n (%) |  |  |  |  | <0.001 |
| 7-8 h/day | 218176 (68.1) | 75031 (70.2) | 72449 (67.8) | 70696 (66.2) |  |
| < 7h/day | 78689 (24.5) | 23937 (22.4) | 26502 (24.8) | 28250 (26.4) |  |
| > 8h/day | 23700 (7.4) | 7887 (7.4) | 7905 (7.4) | 7908 (7.4) |  |
| Physical activity, n (%) |  |  |  |  | <0.001 |
| high | 128154 (40.0) | 43366 (40.6) | 42536 (39.8) | 42252 (39.5) |  |
| moderate | 131275 (41.0) | 43417 (40.6) | 43807 (41.0) | 44051 (41.2) |  |
| low | 61136 (19.1) | 20072 (18.8) | 20513 (19.2) | 20551 (19.2) |  |
| Healthy diet score, n (%) |  |  |  |  | <0.001 |
| 0-2 | 118913 (37.1) | 39197 (36.7) | 40395 (37.8) | 39321 (36.8) |  |
| 3-5 | 190161 (59.3) | 63959 (59.9) | 62788 (58.8) | 63414 (59.3) |  |
| >=6 | 11491 (3.6) | 3699 (3.5) | 3673 (3.4) | 4119 (3.9) |  |
| AMD PRS, n (%) |  |  |  |  | 0.293 |
| low | 106856 (33.3) | 35810 (33.5) | 35664 (33.4) | 35382 (33.1) |  |
| medium | 106854 (33.3) | 35628 (33.3) | 35558 (33.3) | 35668 (33.4) |  |
| high | 106855 (33.3) | 35417 (33.1) | 35634 (33.3) | 35804 (33.5) |  |
| Noise, mean (SD) | 56.03 (4.26) | 54.95 (2.99) | 55.12 (2.94) | 58.02 (5.56) | <0.001 |
| Inverse distance to nearest major road, mean (SD) | 0.01 (0.02) | 0.00 (0.00) | 0.00 (0.01) | 0.01 (0.03) | <0.001 |
| Greenspace, n (%) |  |  |  |  |  |
| first quartile | 80152 (25.0) | 6472 (6.1) | 30114 (28.2) | 43566 (40.8) | <0.001 |
| second quartile | 80131 (25.0) | 13030 (12.2) | 35196 (32.9) | 31905 (29.9) |  |
| third quartile | 80145 (25.0) | 30197 (28.3) | 28842 (27.0) | 21106 (19.8) |  |
| fourth quartile | 80137 (25.0) | 57156 (53.5) | 12704 (11.9) | 10277 (9.6) |  |
| Length of time at current address, n (%) |  | |  |  |  |
| <=10 years | 106488 (33.2) | 36428 (34.1) | 33707 (31.5) | 36353 (34.0) | <0.001 |
| 10-20 years | 84644 (26.4) | 28589 (26.8) | 27729 (25.9) | 28326 (26.5) |  |
| >20 years | 129433 (40.4) | 41838 (39.2) | 45420 (42.5) | 42175 (39.5) |  |
| NO_2_, mean (SD) | 26.38 (7.57) | 19.78 (3.97) | 26.53 (3.98) | 32.83 (7.44) | <0.001 |
| NO_x_, mean (SD) | 43.55 (15.46) | 31.29 (6.70) | 42.84 (6.71) | 56.54 (17.53) | <0.001 |
| PM_10_, mean (SD) | 16.20 (1.90) | 14.51 (1.16) | 16.12 (0.68) | 17.96 (1.75) | <0.001 |
| PM_2.5_, mean (SD) | 9.96 (1.05) | 9.00 (0.52) | 9.97 (0.49) | 10.91 (1.00) | <0.001 |
| PM_2.5-10_, mean (SD) | 6.42 (0.90) | 5.94 (0.33) | 6.18 (0.59) | 7.13 (1.08) | <0.001 |

SES, socioeconomic status; APS1: air pollution score calculated by PCA; APS2: air pollution score calculated by weighted coefficients; NO_2_, nitrogen dioxide; NO_x_, nitrogen oxides; PM_2.5_, particular matter with aerodynamic diameter ≤2.5mm; PM_10_, particular matter with an aerodynamic diameter ≤10mm; PM_2.5-10_, particular matter with aerodynamic diameter 2.5-10 mm. AMD, age-related macular degeneration; PRS, polygenic risk score. Health diet score was calculated based on self-reported servings of fruits, vegetables, whole grains, vegetable oil, fish, dairy, refined grains, unprocessed meats, processed meats and sugar- sweetened beverages. Individual-level SES was constructed by employment, education and household income and calculated by latent class analysis. Townsend Index (including measures of unemployment, non-car ownership, non- home ownership and household overcrowding), derived from respondents’ postcode was used as an indicator of area- level SES.

**Table S7. Hazard ratios and 95% confidence interval for the air pollution scores with incident AMD**

|  | Events | Person years | Model 1 | Model 2 |
| --- | --- | --- | --- | --- |
|  |  |  | HR (95%CI) | HR (95%CI) |
| APS1 |  |  |  |  |
| First tertile | 1211 | 1308913 | Ref. | Ref. |
| Second tertile | 1350 | 1299801 | 1.16 (1.06 - 1.26) | 1.15 (1.06 - 1.26) |
| Third tertile | 1298 | 1298014 | 1.23 (1.11 - 1.36) | 1.22 (1.11 - 1.35) |
| HR per one tertile increase |  |  | 1.11 (1.05-1.16) | 1.10 (1.05-1.16) |
| P for trend |  |  | <0.001 | <0.001 |
|  |  |  |  |  |
| APS2 |  |  |  |  |
| First tertile | 1207 | 1307603 | Ref. | Ref. |
| Second tertile | 1336 | 1300130 | 1.15 (1.06-1.26) | 1.15 (1.05-1.25) |
| Third tertile | 1316 | 1298994 | 1.23 (1.12-1.35) | 1.22 (1.11-1.34) |
| HR per one tertile increase |  |  | 1.11 (1.05-1.16) | 1.10 (1.05-1.16) |
| P for trend |  |  | <0.001 | <0.001 |

CI, confidence interval; HR, hazard ratio. APS1: air pollution score calculated by PCA; APS2: air pollution score calculated by weighted coefficients. AMD, age-related macular degeneration. Model 1 adjusted for age at recruitment, sex, region, body mass index, ethnicity, smoking status, alcohol assumption, physical activity, healthy diet score, sleep duration, noise, green space, inverse distance to nearest major road, and length of time at current address. Model 2 further adjusted for AMD genetic risk score, genotyping array, and the first 10 principal components of ancestry.

**Table S8. Hazard ratios and 95% confidence interval for the air pollution scores with incident OCMD after AMD diagnosis**

|  | Events | Person years | Model 1 | Model 2 |
| --- | --- | --- | --- | --- |
|  |  |  | HR (95%CI) | HR (95%CI) |
| APS1 |  |  |  |  |
| First tertile | 910 | 1310409 | Ref. | Ref. |
| Second tertile | 1018 | 1301375 | 1.18 (1.06 - 1.30) | 1.17 (1.06 - 1.30) |
| Third tertile | 979 | 1299634 | 1.26 (1.13 - 1.42) | 1.26 (1.12 - 1.41) |
| HR per one tertile increase |  |  | 1.12 (1.06-1.19) | 1.12 (1.06-1.19) |
| P for trend |  |  | <0.001 | <0.001 |
|  |  |  |  |  |
| APS2 |  |  |  |  |
| First tertile | 911 | 1309079 | Ref. | Ref. |
| Second tertile | 1003 | 1301719 | 1.15 (1.04 - 1.28) | 1.15 (1.04 - 1.27) |
| Third tertile | 993 | 1300620 | 1.25 (1.12 - 1.39) | 1.25 (1.12 - 1.39) |
| HR per one tertile increase |  |  | 1.11 (1.06-1.18) | 1.11 (1.05-1.18) |
| P for trend |  |  | <0.001 | <0.001 |

CI, confidence interval; HR, hazard ratio. APS1: air pollution score calculated by PCA; APS2: air pollution score calculated by weighted coefficients; AMD, age-related macular degeneration; OCMD, ocular comorbidity. Model 1 adjusted for age at recruitment, sex, region, body mass index, ethnicity, smoking status, alcohol assumption, physical activity, healthy diet score, sleep duration, noise, green space, inverse distance to nearest major road, and length of time at current address. Model 2 further adjusted for AMD genetic risk score, genotyping array, and the first 10 principal components of ancestry.

**Table S9. Hazard ratios and 95% confidence interval for the air pollution scores with all-cause mortality**

|  | Events | Person years | Model 1 | Model 2 |
| --- | --- | --- | --- | --- |
|  |  |  | HR (95%CI) | HR (95%CI) |
| APS1 |  |  |  |  |
| First tertile | 7220 | 1313683 | Ref. | Ref. |
| Second tertile | 7847 | 1305217 | 1.06 (1.03 - 1.11) | 1.06 (1.03 - 1.10) |
| Third tertile | 8296 | 1303248 | 1.16 (1.11 - 1.21) | 1.15 (1.10 - 1.20) |
| HR per one tertile increase |  |  | 1.08 (1.06-1.10) | 1.07 (1.05-1.09) |
| P for trend |  |  | <0.001 | <0.001 |
|  |  |  |  |  |
| APS2 |  |  |  |  |
| First tertile | 7277 | 1312385 | Ref. | Ref. |
| Second tertile | 7796 | 1305465 | 1.05 (1.02 - 1.09) | 1.05 (1.02 - 1.09) |
| Third tertile | 8290 | 1304298 | 1.13 (1.08 - 1.17) | 1.12 (1.08 - 1.16) |
| HR per one tertile increase |  |  | 1.06 (1.04-1.08) | 1.06 (1.04-1.08) |
| P for trend |  |  | <0.001 | <0.001 |

CI, confidence interval; HR, hazard ratio. APS1: air pollution score calculated by PCA; APS2: air pollution score calculated by weighted coefficients. Model 1 adjusted for age at recruitment, sex, region, body mass index, ethnicity, smoking status, alcohol assumption, physical activity, healthy diet score, sleep duration, noise, green space, inverse distance to nearest major road, and length of time at current address. Model 2 further adjusted for AMD genetic risk score, genotyping array, and the first 10 principal components of ancestry.

**Table S10. Hazard ratios and 95% confidence interval for the socioeconomic status with incident AMD**

|  | Events | Person years | Model 1 | Model 2 |
| --- | --- | --- | --- | --- |
|  |  |  | HR (95%CI) | HR (95%CI) |
| Individual-level SES |  |  |  |  |
| high SES | 647 | 1210353 | Ref. | Ref. |
| medium SES | 1793 | 1834022 | 1.09 (1.00 - 1.20) | 1.09 (0.99 - 1.19) |
| low SES | 1419 | 862352 | 1.18 (1.06 - 1.31) | 1.17 (1.05 - 1.30) |
| HR per one tertile increase |  |  | 1.09 (1.03-1.14) | 1.08 (1.03-1.14) |
| P for trend |  |  | 0.001 | 0.003 |
|  |  |  |  |  |
| Area-level SES |  |  |  |  |
| least deprived | 1254 | 1314007 | Ref. | Ref. |
| moderately deprived | 1328 | 1304568 | 1.07 (0.99-1.16) | 1.07 (0.99-1.15) |
| severely deprived | 1277 | 1288152 | 1.09 (1.01-1.18) | 1.08 (1.00-1.17) |
| HR per one tertile increase |  |  | 1.04 (1.00-1.09) | 1.04 (1.00-1.08) |
| P for trend |  |  | 0.035 | 0.041 |

CI, confidence interval; HR, hazard ratio; SES, socioeconomic status; AMD, age-related macular degeneration. Model 1 adjusted for age at recruitment, sex, region, body mass index, ethnicity, smoking status, alcohol assumption, physical activity, healthy diet score, sleep duration. Model 2 further adjusted for AMD genetic risk score, genotyping array, and the first 10 principal components of ancestry.

**Table S11. Hazard ratios and 95% confidence interval for the socioeconomic status with incident OCMD after AMD diagnosis**

|  | Events | Person years | Model 1 | Model 2 |
| --- | --- | --- | --- | --- |
|  |  |  | HR (95%CI) | HR (95%CI) |
| Individual-level SES |  |  |  |  |
| high SES | 432 | 1211413 | Ref. | Ref. |
| medium SES | 1363 | 1836092 | 1.20 (1.07 - 1.34) | 1.19 (1.06 - 1.33) |
| low SES | 1112 | 863913 | 1.30 (1.15 - 1.47) | 1.29 (1.14 - 1.46) |
| HR per one tertile increase |  |  | 1.13 (1.06-1.20) | 1.12 (1.06-1.19) |
| P for trend |  |  | <0.001 | <0.001 |
|  |  |  |  |  |
| Area-level SES |  |  |  |  |
| least deprived | 954 | 1315489 | Ref. | Ref. |
| moderately deprived | 984 | 1306205 | 1.04 (0.96-1.14) | 1.04 (0.95-1.14) |
| severely deprived | 969 | 1289724 | 1.10 (1.00-1.20) | 1.09 (1.00-1.20) |
| HR per one tertile increase |  |  | 1.05 (1.00-1.10) | 1.04 (1.00-1.09) |
| P for trend |  |  | 0.053 | 0.062 |

CI, confidence interval; HR, hazard ratio; AMD, age-related macular degeneration; OCMD, ocular comorbidity. Model 1 adjusted for age at recruitment, sex, region, body mass index, ethnicity, smoking status, alcohol assumption, physical activity, healthy diet score, sleep duration. Model 2 further adjusted for AMD genetic risk score, genotyping array, and the first 10 principal components of ancestry.

**Table S12. Hazard ratios and 95% confidence interval for the socioeconomic status with all-cause mortality**

|  | Events | Person years | Model 1 | Model 2 |
| --- | --- | --- | --- | --- |
|  |  |  | HR (95%CI) | HR (95%CI) |
| Individual-level SES |  |  |  |  |
| high SES | 3612 | 1213026 | Ref. | Ref. |
| medium SES | 10103 | 1841012 | 1.31 (1.26 - 1.36) | 1.30 (1.25 - 1.36) |
| low SES | 9648 | 868111 | 1.84 (1.76 - 1.92) | 1.83 (1.76 - 1.91) |
| HR per one tertile increase |  |  | 1.37 (1.34-1.40) | 1.37 (1.34-1.39) |
| P for trend |  |  | <0.001 | <0.001 |
|  |  |  |  |  |
| Area-level SES |  |  |  |  |
| least deprived | 6757 | 1319066 | Ref. | Ref. |
| moderately deprived | 7342 | 1309861 | 1.08 (1.05-1.12) | 1.08 (1.04-1.12) |
| severely deprived | 9264 | 1293222 | 1.34 (1.30-1.39) | 1.33 (1.29-1.38) |
| HR per one tertile increase |  |  | 1.16 (1.14-1.18) | 1.16 (1.14-1.18) |
| P for trend |  |  | <0.001 | <0.001 |

CI, confidence interval; HR, hazard ratio; SES, socioeconomic status. Model 1 adjusted for age at recruitment, sex, region, body mass index, ethnicity, smoking status, alcohol assumption, physical activity, healthy diet score, sleep duration. Model 2 further adjusted for AMD genetic risk score, genotyping array, and the first 10 principal components of ancestry.

**Table S13. Associations of APS1 and socioeconomic status with transitions (further adjusted for prevalent diseases)**

|  |  | **Baseline → AMD** | | **Baseline → Death** | | **AMD → OCMD** | |
| --- | --- | --- | --- | --- | --- | --- | --- |
| **APS1** | **Individual-level SES** | **Model 2** | **Model 2a** | **Model 2** | **Model 2a** | **Model 2** | **Model 2a** |
|  |  | **HR (95% CI)** | **HR (95% CI)** | **HR (95% CI)** | **HR (95% CI)** | **HR (95% CI)** | **HR (95% CI)** |
| First tertile | High | Ref. | Ref. | Ref. | Ref. | Ref. | Ref. |
|  | Medium | 1.11 (0.93-1.31) | 1.09 (0.90-1.32) | **1.24 (1.17-1.33)** | **1.22 (1.14-1.30)** | **1.62 (1.32-1.98)** | **1.62 (1.32-1.99)** |
|  | Low | 1.12 (0.96-1.32) | 1.11 (0.95-1.32) | **1.71 (1.60-1.84)** | **1.61 (1.50-1.73)** | **1.77 (1.43-2.18)** | **1.77 (1.43-2.18)** |
| Second tertile | High | 1.14 (0.94-1.39) | 1.14 (0.94-1.39) | 0.99 (0.91-1.08) | 0.99 (0.91-1.07) | **1.29 (1.02-1.64)** | **1.29 (1.01-1.64)** |
|  | Medium | **1.23 (1.05-1.45)** | **1.22 (1.04-1.43)** | **1.29 (1.21-1.38)** | **1.25 (1.17-1.34)** | **1.67 (1.39-2.02)** | **1.69 (1.40-2.04)** |
|  | Low | **1.35 (1.14-1.60)** | **1.31 (1.10-1.55)** | **1.78 (1.66-1.91)** | **1.65 (1.54-1.77)** | **1.83 (1.51-2.23)** | **1.84 (1.52-2.24)** |
| Third tertile | High | **1.25 (1.03-1.53)** | **1.25 (1.03-1.52)** | 0.99 (0.91-1.08) | 0.99 (0.91-1.07) | **1.40 (1.10-1.79)** | **1.39 (1.09-1.78)** |
|  | Medium | **1.30 (1.10-1.54)** | **1.28 (1.09-1.52)** | **1.37 (1.28-1.47)** | **1.34 (1.25-1.43)** | **1.78 (1.46-2.19)** | **1.81 (1.47-2.23)** |
|  | Low | **1.41 (1.18-1.67)** | **1.35 (1.13-1.60)** | **1.93 (1.80-2.07)** | **1.76 (1.64-1.89)** | **1.91 (1.55-2.36)** | **1.87 (1.52-2.32)** |
| **APS1** | **Area-level SES** | **Model 2** | **Model 2a** | **Model 2** | **Model 2a** | **Model 2** | **Model 2a** |
|  |  | **HR (95% CI)** | **HR (95% CI)** | **HR (95% CI)** | **HR (95% CI)** | **HR (95% CI)** | **HR (95% CI)** |
| First tertile | least deprived | Ref. | Ref. | Ref. | Ref. | Ref. | Ref. |
|  | moderately deprived | 1.10 (0.95-1.25) | 1.09 (0.97-1.24) | **1.06 (1.00-1.11)** | **1.05 (1.00-1.11)** | 1.01 (0.83-1.24) | 1.01 (0.83-1.24) |
|  | severely deprived | 1.12 (0.97-1.33) | 1.12 (0.95-1.32) | **1.25 (1.18-1.34)** | **1.20 (1.12-1.28)** | 1.04 (0.88-1.23) | 1.04 (0.88-1.23) |
| Second tertile | least deprived | **1.20 (1.05-1.38)** | **1.21 (1.06-1.38)** | 1.00 (0.95-1.06) | 0.99 (0.94-1.05) | 0.96 (0.81-1.13) | 0.96 (0.81-1.13) |
|  | moderately deprived | **1.22 (1.07-1.39)** | **1.22 (1.08-1.39)** | **1.08 (1.02-1.14)** | **1.06 (1.01-1.13)** | 1.13 (0.97-1.31) | 1.13 (0.97-1.31) |
|  | severely deprived | **1.23 (1.09-1.40)** | **1.23 (1.09-1.40)** | **1.38 (1.31-1.46)** | **1.31 (1.24-1.38)** | **1.19 (1.02-1.39)** | **1.19 (1.01-1.40)** |
| Third tertile | least deprived | **1.24 (1.05-1.46)** | **1.24 (1.05-1.46)** | 1.03 (0.96-1.11) | 1.02 (0.95-1.10) | 0.97 (0.84-1.12) | 0.97 (0.84-1.12) |
|  | moderately deprived | **1.30 (1.12-1.50)** | **1.30 (1.12-1.50)** | **1.15 (1.08-1.22)** | **1.13 (1.06-1.20)** | **1.19 (1.01-1.40)** | 1.18 (0.99-1.43) |
|  | severely deprived | **1.31 (1.15-1.49)** | **1.31 (1.15-1.49)** | **1.38 (1.32-1.46)** | **1.32 (1.25-1.40)** | **1.21 (1.03-1.47)** | **1.20 (1.02-1.45)** |

CI, confidence interval; HR, hazard ratio. APS1: air pollution score calculated by PCA; APS2: air pollution score calculated by weighted coefficients; SES, socioeconomic status; AMD, age-related macular degeneration; OCMD, ocular comorbidity. Model 2 adjusted for age at recruitment, sex, region, body mass index, ethnicity, smoking status, alcohol assumption, physical activity, healthy diet score, sleep duration, noise, green space, inverse distance to nearest major road, and length of time at current address, AMD genetic risk score, genotyping array, and the first 10 principal components of ancestry. Model 2a further adjusted for prevalent hypertension, diabetes, cardiovascular disease, asthma, chronic obstructive pulmonary disease, depression and anxiety.

**Table S14. Associations of APS2 and socioeconomic status with transitions (further adjusted for prevalent diseases)**

|  |  | **Baseline → AMD** | | **Baseline → Death** | | **AMD → OCMD** | |
| --- | --- | --- | --- | --- | --- | --- | --- |
| **APS2** | **Individual-level SES** | **Model 2** | **Model 2a** | **Model 2** | **Model 2a** | **Model 2** | **Model 2a** |
|  |  | **HR (95% CI)** | **HR (95% CI)** | **HR (95% CI)** | **HR (95% CI)** | **HR (95% CI)** | **HR (95% CI)** |
| First tertile | High | Ref. | Ref. | Ref. | Ref. | Ref. | Ref. |
|  | Medium | **1.17 (1.00-1.38)** | 1.16 (0.99-1.36) | **1.24 (1.17-1.33)** | **1.22 (1.14-1.30)** | **1.64 (1.33-2.02)** | **1.65 (1.34-2.04)** |
|  | Low | **1.23 (1.03-1.46)** | **1.19 (1.00-1.42)** | **1.71 (1.59-1.83)** | **1.60 (1.49-1.72)** | **1.69 (1.39-2.05)** | **1.71 (1.41-2.08)** |
| Second tertile | High | **1.30 (1.07-1.58)** | **1.31 (1.08-1.58)** | 0.96 (0.89-1.05) | 0.96 (0.89-1.04) | **1.32 (1.04-1.69)** | **1.31 (1.03-1.68)** |
|  | Medium | **1.32 (1.12-1.55)** | **1.31 (1.10-1.55)** | **1.27 (1.19-1.36)** | **1.24 (1.16-1.33)** | **1.81 (1.46-2.24)** | **1.83 (1.47-2.27)** |
|  | Low | **1.35 (1.13-1.60)** | **1.33 (1.12-1.58)** | **1.76 (1.65-1.89)** | **1.64 (1.53-1.75)** | **1.92 (1.57-2.34)** | **1.93 (1.58-2.36)** |
| Third tertile | High | **1.30 (1.07-1.58)** | **1.30 (1.06-1.58)** | 0.98 (0.90-1.06) | 0.98 (0.90-1.06) | **1.51 (1.19-1.93)** | **1.52 (1.19-1.94)** |
|  | Medium | **1.36 (1.15-1.61)** | **1.34 (1.14-1.59)** | **1.34 (1.25-1.43)** | **1.30 (1.22-1.39)** | **1.85 (1.55-2.30)** | **1.92 (1.56-2.37)** |
|  | Low | **1.51 (1.27-1.80)** | **1.45 (1.22-1.73)** | **1.89 (1.77-2.03)** | **1.74 (1.62-1.86)** | **2.06 (1.67-2.55)** | **2.03 (1.64-2.51)** |
| **APS2** | **Area-level SES** | **Model 2** | **Model 2a** | **Model 2** | **Model 2a** | **Model 2** | **Model 2a** |
|  |  | **HR (95% CI)** | **HR (95% CI)** | **HR (95% CI)** | **HR (95% CI)** | **HR (95% CI)** | **HR (95% CI)** |
| First tertile | least deprived | Ref. | Ref. | Ref. | Ref. | Ref. | Ref. |
|  | moderately deprived | 1.10 (0.93-1.29) | 1.08 (0.91-1.27) | **1.05 (1.00-1.11)** | 1.04 (0.99-1.10) | 1.02 (0.86-1.24) | 1.02 (0.86-1.24) |
|  | severely deprived | 1.12 (0.99-1.27) | 1.12 (0.99-1.27) | **1.27 (1.20-1.36)** | **1.21 (1.14-1.29)** | 1.04 (0.88-1.25) | 1.03 (0.87-1.25) |
| Second tertile | least deprived | **1.19 (1.04-1.36)** | **1.19 (1.04-1.35)** | 0.99 (0.93-1.05) | 0.98 (0.93-1.04) | 0.95 (0.82-1.10) | 0.94 (0.81-1.10) |
|  | moderately deprived | **1.22 (1.07-1.40)** | **1.23 (1.08-1.39)** | **1.08 (1.02-1.14)** | **1.06 (1.00-1.12)** | 1.13 (0.97-1.31) | 1.13 (0.97-1.31) |
|  | severely deprived | **1.23 (1.09-1.40)** | **1.23 (1.09-1.40)** | **1.35 (1.28-1.43)** | **1.29 (1.22-1.36)** | **1.19 (1.02-1.39)** | **1.18 (1.02-1.38)** |
| Third tertile | least deprived | **1.28 (1.11-1.47)** | **1.26 (1.10-1.45)** | 1.02 (0.95-1.09) | 1.01 (0.94-1.08) | 0.97 (0.83-1.13) | 0.96 (0.83-1.12) |
|  | moderately deprived | **1.29 (1.11-1.51)** | **1.28 (1.12-1.46)** | **1.13 (1.06-1.19)** | **1.10 (1.04-1.17)** | **1.19 (1.01-1.40)** | 1.18 (0.99-1.37) |
|  | severely deprived | **1.31 (1.15-1.49)** | **1.29 (1.12-1.50)** | **1.35 (1.28-1.43)** | **1.30 (1.24-1.40)** | **1.21 (1.03-1.47)** | **1.20 (1.02-1.46)** |

CI, confidence interval; HR, hazard ratio. APS1: air pollution score calculated by PCA; APS2: air pollution score calculated by weighted coefficients; AMD, age-related macular degeneration; OCMD, ocular comorbidity. Model 2 adjusted for age at recruitment, sex, region, body mass index, ethnicity, smoking status, alcohol assumption, physical activity, healthy diet score, sleep duration, noise, green space, inverse distance to nearest major road, and length of time at current address, AMD genetic risk score, genotyping array, and the first 10 principal components of ancestry. Model 2a further adjusted for prevalent hypertension, diabetes, cardiovascular disease, asthma, chronic obstructive pulmonary disease, depression and anxiety.

**Table S15. Associations of APS1 and socioeconomic status with transitions (further adjusted for solid fuel use and passive smoking)**

|  |  | **Baseline → AMD** | | **Baseline → Death** | | **AMD → OCMD** | |
| --- | --- | --- | --- | --- | --- | --- | --- |
| **APS1** | **Individual-level SES** | **Model 2** | **Model 2b** | **Model 2** | **Model 2b** | **Model 2** | **Model 2b** |
|  |  | **HR (95% CI)** | **HR (95% CI)** | **HR (95% CI)** | **HR (95% CI)** | **HR (95% CI)** | **HR (95% CI)** |
| First tertile | High | Ref. | Ref. | Ref. | Ref. | Ref. | Ref. |
|  | Medium | 1.11 (0.93-1.31) | 1.11 (0.93-1.31) | **1.24 (1.17-1.33)** | **1.24 (1.17-1.33)** | **1.62 (1.32-1.98)** | **1.62 (1.32-1.97)** |
|  | Low | 1.12 (0.96-1.32) | 1.12 (0.96-1.32) | **1.71 (1.60-1.84)** | **1.70 (1.60-1.83)** | **1.77 (1.43-2.18)** | **1.77 (1.43-2.17)** |
| Second tertile | High | 1.14 (0.94-1.39) | 1.14 (0.94-1.39) | 0.99 (0.91-1.08) | 0.99 (0.91-1.08) | **1.29 (1.02-1.64)** | **1.29 (1.02-1.64)** |
|  | Medium | **1.23 (1.05-1.45)** | **1.22 (1.04-1.44)** | **1.29 (1.21-1.38)** | **1.29 (1.21-1.37)** | **1.67 (1.39-2.02)** | **1.67 (1.39-2.02)** |
|  | Low | **1.35 (1.14-1.60)** | **1.35 (1.14-1.60)** | **1.78 (1.66-1.91)** | **1.77 (1.66-1.90)** | **1.83 (1.51-2.23)** | **1.82 (1.51-2.22)** |
| Third tertile | High | **1.25 (1.03-1.53)** | **1.24 (1.03-1.52)** | 0.99 (0.91-1.08) | 0.99 (0.90-1.07) | **1.40 (1.10-1.79)** | **1.40 (1.10-1.78)** |
|  | Medium | **1.30 (1.10-1.54)** | **1.30 (1.10-1.54)** | **1.37 (1.28-1.47)** | **1.37 (1.28-1.47)** | **1.78 (1.46-2.19)** | **1.77 (1.45-2.18)** |
|  | Low | **1.41 (1.18-1.67)** | **1.40 (1.18-1.66)** | **1.93 (1.80-2.07)** | **1.93 (1.80-2.06)** | **1.91 (1.55-2.36)** | **1.90 (1.54-2.35)** |
| **APS1** | **Area-level SES** | **Model 2** | **Model 2b** | **Model 2** | **Model 2b** | **Model 2** | **Model 2b** |
|  |  | **HR (95% CI)** | **HR (95% CI)** | **HR (95% CI)** | **HR (95% CI)** | **HR (95% CI)** | **HR (95% CI)** |
| First tertile | least deprived | Ref. | Ref. | Ref. | Ref. | Ref. | Ref. |
|  | moderately deprived | 1.10 (0.95-1.25) | 1.10 (0.95-1.25) | **1.06 (1.00-1.11)** | **1.06 (1.00-1.11)** | 1.01 (0.83-1.24) | 1.01 (0.83-1.23) |
|  | severely deprived | 1.12 (0.97-1.33) | 1.12 (0.97-1.33) | **1.25 (1.18-1.34)** | **1.25 (1.18-1.34)** | 1.04 (0.88-1.23) | 1.03 (0.87-1.23) |
| Second tertile | least deprived | **1.20 (1.05-1.38)** | **1.20 (1.04-1.38)** | 1.00 (0.95-1.06) | 1.00 (0.95-1.06) | 0.96 (0.81-1.13) | 0.96 (0.81-1.13) |
|  | moderately deprived | **1.22 (1.07-1.39)** | **1.22 (1.06-1.39)** | **1.08 (1.02-1.14)** | **1.08 (1.02-1.14)** | 1.13 (0.97-1.31) | 1.12 (0.96-1.30) |
|  | severely deprived | **1.23 (1.09-1.40)** | **1.23 (1.09-1.40)** | **1.38 (1.31-1.46)** | **1.38 (1.31-1.46)** | **1.19 (1.02-1.39)** | **1.19 (1.02-1.38)** |
| Third tertile | least deprived | **1.24 (1.05-1.46)** | **1.24 (1.05-1.45)** | 1.03 (0.96-1.11) | 1.03 (0.96-1.11) | 0.97 (0.84-1.12) | 0.97 (0.84-1.12) |
|  | moderately deprived | **1.30 (1.12-1.50)** | **1.30 (1.12-1.50)** | **1.15 (1.08-1.22)** | **1.15 (1.08-1.22)** | **1.19 (1.01-1.40)** | **1.19 (1.01-1.40)** |
|  | severely deprived | **1.31 (1.15-1.49)** | **1.31 (1.15-1.49)** | **1.38 (1.32-1.46)** | **1.38 (1.32-1.46)** | **1.21 (1.03-1.47)** | **1.21 (1.03-1.46)** |

CI, confidence interval; HR, hazard ratio. APS1: air pollution score calculated by PCA; APS2: air pollution score calculated by weighted coefficients; SES, socioeconomic status; AMD, age-related macular degeneration; OCMD, ocular comorbidity. Model 2 adjusted for age at recruitment, sex, region, body mass index, ethnicity, smoking status, alcohol assumption, physical activity, healthy diet score, sleep duration, noise, green space, inverse distance to nearest major road, and length of time at current address, AMD genetic risk score, genotyping array, and the first 10 principal components of ancestry. Model 2b further adjusted for solid fuel use and passive smoking.

**Table S16. Associations of APS2 and socioeconomic status with transitions (further adjusted for solid fuel use and passive smoking)**

|  |  | **Baseline → AMD** | | **Baseline → Death** | | **AMD → OCMD** | |
| --- | --- | --- | --- | --- | --- | --- | --- |
| **APS2** | **Individual-level SES** | **Model 2** | **Model 2b** | **Model 2** | **Model 2b** | **Model 2** | **Model 2b** |
|  |  | **HR (95% CI)** | **HR (95% CI)** | **HR (95% CI)** | **HR (95% CI)** | **HR (95% CI)** | **HR (95% CI)** |
| First tertile | High | Ref. | Ref. | Ref. | Ref. | Ref. | Ref. |
|  | Medium | **1.17 (1.00-1.38)** | **1.16 (1.00-1.37)** | **1.24 (1.17-1.33)** | **1.23 (1.16-1.32)** | **1.64 (1.33-2.02)** | **1.63 (1.32-2.01)** |
|  | Low | **1.23 (1.03-1.46)** | **1.22 (1.03-1.45)** | **1.71 (1.59-1.83)** | **1.71 (1.58-1.83)** | **1.69 (1.39-2.05)** | **1.68 (1.38-2.04)** |
| Second tertile | High | **1.30 (1.07-1.58)** | **1.30 (1.07-1.58)** | 0.96 (0.89-1.05) | 0.95 (0.89-1.04) | **1.32 (1.04-1.69)** | **1.31 (1.03-1.68)** |
|  | Medium | **1.32 (1.12-1.55)** | **1.32 (1.12-1.55)** | **1.27 (1.19-1.36)** | **1.27 (1.19-1.35)** | **1.81 (1.46-2.24)** | **1.80 (1.45-2.23)** |
|  | Low | **1.35 (1.13-1.60)** | **1.35 (1.13-1.60)** | **1.76 (1.65-1.89)** | **1.77 (1.66-1.89)** | **1.92 (1.57-2.34)** | **1.91 (1.56-2.33)** |
| Third tertile | High | **1.30 (1.07-1.58)** | **1.30 (1.07-1.58)** | 0.98 (0.90-1.06) | 0.98 (0.90-1.06) | **1.51 (1.19-1.93)** | **1.50 (1.18-1.92)** |
|  | Medium | **1.36 (1.15-1.61)** | **1.35 (1.14-1.60)** | **1.34 (1.25-1.43)** | **1.33 (1.24-1.42)** | **1.85 (1.55-2.30)** | **1.84 (1.54-2.29)** |
|  | Low | **1.51 (1.27-1.80)** | **1.51 (1.27-1.80)** | **1.89 (1.77-2.03)** | **1.89 (1.77-2.03)** | **2.06 (1.67-2.55)** | **2.05 (1.66-2.54)** |
| **APS2** | **Area-level SES** | **Model 2** | **Model 2b** | **Model 2** | **Model 2b** | **Model 2** | **Model 2b** |
|  |  | **HR (95% CI)** | **HR (95% CI)** | **HR (95% CI)** | **HR (95% CI)** | **HR (95% CI)** | **HR (95% CI)** |
| First tertile | least deprived | Ref. | Ref. | Ref. | Ref. | Ref. | Ref. |
|  | moderately deprived | 1.10 (0.93-1.29) | 1.10 (0.93-1.29) | **1.05 (1.00-1.11)** | **1.05 (1.00-1.10)** | 1.02 (0.86-1.24) | 1.02 (0.86-1.24) |
|  | severely deprived | 1.12 (0.99-1.27) | 1.12 (0.99-1.27) | **1.27 (1.20-1.36)** | **1.26 (1.20-1.35)** | 1.04 (0.88-1.25) | 1.04 (0.88-1.25) |
| Second tertile | least deprived | **1.19 (1.04-1.36)** | **1.19 (1.04-1.36)** | 0.99 (0.93-1.05) | 0.99 (0.93-1.05) | 0.95 (0.82-1.10) | 0.95 (0.82-1.10) |
|  | moderately deprived | **1.22 (1.07-1.40)** | **1.22 (1.07-1.40)** | **1.08 (1.02-1.14)** | **1.08 (1.02-1.14)** | 1.13 (0.97-1.31) | 1.13 (0.97-1.30) |
|  | severely deprived | **1.23 (1.09-1.40)** | **1.23 (1.09-1.40)** | **1.35 (1.28-1.43)** | **1.34 (1.27-1.42)** | **1.19 (1.02-1.39)** | **1.19 (1.02-1.38)** |
| Third tertile | least deprived | **1.28 (1.11-1.47)** | **1.28 (1.11-1.47)** | 1.02 (0.95-1.09) | 1.02 (0.95-1.09) | 0.97 (0.83-1.13) | 0.97 (0.83-1.13) |
|  | moderately deprived | **1.29 (1.11-1.51)** | **1.29 (1.11-1.51)** | **1.13 (1.06-1.19)** | **1.13 (1.06-1.19)** | **1.19 (1.01-1.40)** | **1.19 (1.01-1.40)** |
|  | severely deprived | **1.31 (1.15-1.49)** | **1.31 (1.15-1.49)** | **1.35 (1.28-1.43)** | **1.35 (1.28-1.43)** | **1.21 (1.03-1.47)** | **1.21 (1.03-1.46)** |

CI, confidence interval; HR, hazard ratio. APS1: air pollution score calculated by PCA; APS2: air pollution score calculated by weighted coefficients; AMD, age-related macular degeneration; OCMD, ocular comorbidity. Model 2 adjusted for age at recruitment, sex, region, body mass index, ethnicity, smoking status, alcohol assumption, physical activity, healthy diet score, sleep duration, noise, green space, inverse distance to nearest major road, and length of time at current address, AMD genetic risk score, genotyping array, and the first 10 principal components of ancestry. Model 2b further adjusted for solid fuel use and passive smoking.

**Table S17. Associations of APS1 and socioeconomic status with transitions (mutually adjusted for SES)**

|  |  | **Baseline → AMD** | | **Baseline → Death** | | **AMD → OCMD** | |
| --- | --- | --- | --- | --- | --- | --- | --- |
| **APS1** | **Individual-level SES** | **Model 2** | **Model 2c** | **Model 2** | **Model 2c** | **Model 2** | **Model 2c** |
|  |  | **HR (95% CI)** | **HR (95% CI)** | **HR (95% CI)** | **HR (95% CI)** | **HR (95% CI)** | **HR (95% CI)** |
| First tertile | High | Ref. | Ref. | Ref. | Ref. | Ref. | Ref. |
|  | Medium | 1.11 (0.93-1.31) | 1.10 (0.92-1.31) | **1.24 (1.17-1.33)** | **1.23 (1.15-1.31)** | **1.62 (1.32-1.98)** | **1.61 (1.32-1.98)** |
|  | Low | 1.12 (0.96-1.32) | 1.12 (0.96-1.33) | **1.71 (1.60-1.84)** | **1.65 (1.54-1.77)** | **1.77 (1.43-2.18)** | **1.77 (1.43-2.18)** |
| Second tertile | High | 1.14 (0.94-1.39) | 1.14 (0.93-1.38) | 0.99 (0.91-1.08) | 0.98 (0.90-1.06) | **1.29 (1.02-1.64)** | **1.29 (1.01-1.64)** |
|  | Medium | **1.23 (1.05-1.45)** | **1.23 (1.04-1.44)** | **1.29 (1.21-1.38)** | **1.25 (1.17-1.34)** | **1.67 (1.39-2.02)** | **1.67 (1.38-2.02)** |
|  | Low | **1.35 (1.14-1.60)** | **1.34 (1.13-1.58)** | **1.78 (1.66-1.91)** | **1.68 (1.57-1.80)** | **1.83 (1.51-2.23)** | **1.83 (1.51-2.23)** |
| Third tertile | High | **1.25 (1.03-1.53)** | **1.24 (1.02-1.51)** | 0.99 (0.91-1.08) | 0.99 (0.91-1.08) | **1.40 (1.10-1.79)** | **1.40 (1.10-1.79)** |
|  | Medium | **1.30 (1.10-1.54)** | **1.29 (1.09-1.53)** | **1.37 (1.28-1.47)** | **1.29 (1.20-1.38)** | **1.78 (1.46-2.19)** | **1.78 (1.45-2.19)** |
|  | Low | **1.41 (1.18-1.67)** | **1.39 (1.16-1.65)** | **1.93 (1.80-2.07)** | **1.76 (1.64-1.89)** | **1.91 (1.55-2.36)** | **1.91 (1.55-2.35)** |
| **APS1** | **Area-level SES** | **Model 2** | **Model 2c** | **Model 2** | **Model 2c** | **Model 2** | **Model 2c** |
|  |  | **HR (95% CI)** | **HR (95% CI)** | **HR (95% CI)** | **HR (95% CI)** | **HR (95% CI)** | **HR (95% CI)** |
| First tertile | least deprived | Ref. | Ref. | Ref. | Ref. | Ref. | Ref. |
|  | moderately deprived | 1.10 (0.95-1.25) | 1.09 (0.97-1.24) | **1.06 (1.00-1.11)** | 1.03 (0.98-1.08) | 1.01 (0.83-1.24) | 1.01 (0.83-1.24) |
|  | severely deprived | 1.12 (0.97-1.33) | 1.11 (0.94-1.31) | **1.25 (1.18-1.34)** | **1.15 (1.08-1.23)** | 1.04 (0.88-1.23) | 1.04 (0.88-1.23) |
| Second tertile | least deprived | **1.20 (1.05-1.38)** | **1.21 (1.07-1.38)** | 1.00 (0.95-1.06) | 0.98 (0.92-1.03) | 0.96 (0.81-1.13) | 0.96 (0.81-1.13) |
|  | moderately deprived | **1.22 (1.07-1.39)** | **1.22 (1.07-1.39)** | **1.08 (1.02-1.14)** | **1.06 (1.01-1.13)** | 1.13 (0.97-1.31) | 1.11 (0.95-1.27) |
|  | severely deprived | **1.23 (1.09-1.40)** | **1.23 (1.09-1.40)** | **1.38 (1.31-1.46)** | **1.25 (1.18-1.32)** | **1.19 (1.02-1.39)** | **1.18 (1.01-1.39)** |
| Third tertile | least deprived | **1.24 (1.05-1.46)** | **1.23 (1.04-1.45)** | 1.03 (0.96-1.11) | 1.00 (0.94-1.08) | 0.97 (0.84-1.12) | 0.97 (0.84-1.12) |
|  | moderately deprived | **1.30 (1.12-1.50)** | **1.28 (1.11-1.48)** | **1.15 (1.08-1.22)** | **1.08 (1.02-1.15)** | **1.19 (1.01-1.40)** | 1.18 (0.99-1.43) |
|  | severely deprived | **1.31 (1.15-1.49)** | **1.30 (1.14-1.49)** | **1.38 (1.32-1.46)** | **1.24 (1.18-1.31)** | **1.21 (1.03-1.47)** | **1.20 (1.02-1.45)** |

CI, confidence interval; HR, hazard ratio. APS1: air pollution score calculated by PCA; APS2: air pollution score calculated by weighted coefficients; AMD, age-related macular degeneration; OCMD, ocular comorbidity. Model 2 adjusted for age at recruitment, sex, region, body mass index, ethnicity, smoking status, alcohol assumption, physical activity, healthy diet score, sleep duration, noise, green space, inverse distance to nearest major road, and length of time at current address, AMD genetic risk score, genotyping array, and the first 10 principal components of ancestry. Model 2c further mutually adjusted for individual-level SES and area-level SES.

**Table S18. Associations of APS2 and socioeconomic status with transitions (mutually adjusted for SES)**

|  |  | **Baseline → AMD** | | **Baseline → Death** | | **AMD → OCMD** | |
| --- | --- | --- | --- | --- | --- | --- | --- |
| **APS2** | **Individual-level SES** | **Model 2** | **Model 2c** | **Model 2** | **Model 2c** | **Model 2** | **Model 2c** |
|  |  | **HR (95% CI)** | **HR (95% CI)** | **HR (95% CI)** | **HR (95% CI)** | **HR (95% CI)** | **HR (95% CI)** |
| First tertile | High | Ref. | Ref. | Ref. | Ref. | Ref. | Ref. |
|  | Medium | **1.17 (1.00-1.38)** | **1.17 (1.00-1.37)** | **1.24 (1.17-1.33)** | **1.23 (1.15-1.31)** | **1.64 (1.33-2.02)** | **1.63 (1.32-2.01)** |
|  | Low | **1.23 (1.03-1.46)** | **1.22 (1.02-1.45)** | **1.71 (1.59-1.83)** | **1.64 (1.53-1.75)** | **1.69 (1.39-2.05)** | **1.68 (1.38-2.04)** |
| Second tertile | High | **1.30 (1.07-1.58)** | **1.29 (1.07-1.57)** | 0.96 (0.89-1.05) | 0.95 (0.88-1.03) | **1.32 (1.04-1.69)** | **1.31 (1.03-1.69)** |
|  | Medium | **1.32 (1.12-1.55)** | **1.31 (1.11-1.54)** | **1.27 (1.19-1.36)** | **1.24 (1.16-1.32)** | **1.81 (1.46-2.24)** | **1.81 (1.46-2.24)** |
|  | Low | **1.35 (1.13-1.60)** | **1.33 (1.12-1.58)** | **1.76 (1.65-1.89)** | **1.67 (1.55-1.78)** | **1.92 (1.57-2.34)** | **1.91 (1.57-2.34)** |
| Third tertile | High | **1.30 (1.07-1.58)** | **1.30 (1.06-1.58)** | 0.98 (0.90-1.06) | 0.98 (0.90-1.06) | **1.51 (1.19-1.93)** | **1.51 (1.19-1.92)** |
|  | Medium | **1.36 (1.15-1.61)** | **1.35 (1.14-1.60)** | **1.34 (1.25-1.43)** | **1.26 (1.18-1.35)** | **1.85 (1.55-2.30)** | **1.89 (1.54-2.33)** |
|  | Low | **1.51 (1.27-1.80)** | **1.49 (1.25-1.78)** | **1.89 (1.77-2.03)** | **1.74 (1.62-1.86)** | **2.06 (1.67-2.55)** | **2.06 (1.66-2.55)** |
| **APS2** | **Area-level SES** | **Model 2** | **Model 2c** | **Model 2** | **Model 2c** | **Model 2** | **Model 2c** |
|  |  | **HR (95% CI)** | **HR (95% CI)** | **HR (95% CI)** | **HR (95% CI)** | **HR (95% CI)** | **HR (95% CI)** |
| First tertile | least deprived | Ref. | Ref. | Ref. | Ref. | Ref. | Ref. |
|  | moderately deprived | 1.10 (0.93-1.29) | 1.08 (0.92-1.27) | **1.05 (1.00-1.11)** | 1.02 (0.97-1.08) | 1.02 (0.86-1.24) | 1.01 (0.85-1.22) |
|  | severely deprived | 1.12 (0.99-1.27) | 1.12 (0.99-1.26) | **1.27 (1.20-1.36)** | **1.17 (1.10-1.24)** | 1.04 (0.88-1.25) | 1.03 (0.87-1.25) |
| Second tertile | least deprived | **1.19 (1.04-1.36)** | **1.18 (1.04-1.35)** | 0.99 (0.93-1.05) | 0.97 (0.91-1.02) | 0.95 (0.82-1.10) | 0.94 (0.81-1.10) |
|  | moderately deprived | **1.22 (1.07-1.40)** | **1.20 (1.05-1.38)** | **1.08 (1.02-1.14)** | 1.02 (0.97-1.08) | 1.13 (0.97-1.31) | 1.13 (0.97-1.31) |
|  | severely deprived | **1.23 (1.09-1.40)** | **1.22 (1.07-1.39)** | **1.35 (1.28-1.43)** | **1.23 (1.16-1.30)** | **1.19 (1.02-1.39)** | **1.18 (1.02-1.38)** |
| Third tertile | least deprived | **1.28 (1.11-1.47)** | **1.26 (1.09-1.45)** | 1.02 (0.95-1.09) | 0.99 (0.93-1.06) | 0.97 (0.83-1.13) | 0.96 (0.83-1.12) |
|  | moderately deprived | **1.29 (1.11-1.51)** | **1.29 (1.10-1.50)** | **1.13 (1.06-1.19)** | **1.06 (1.00-1.13)** | **1.19 (1.01-1.40)** | 1.18 (0.99-1.37) |
|  | severely deprived | **1.31 (1.15-1.49)** | **1.30 (1.12-1.50)** | **1.35 (1.28-1.43)** | **1.23 (1.17-1.30)** | **1.21 (1.03-1.47)** | **1.20 (1.02-1.46)** |

CI, confidence interval; HR, hazard ratio. APS1: air pollution score calculated by PCA; APS2: air pollution score calculated by weighted coefficients; AMD, age-related macular degeneration; OCMD, ocular comorbidity. Model 2 adjusted for age at recruitment, sex, region, body mass index, ethnicity, smoking status, alcohol assumption, physical activity, healthy diet score, sleep duration, noise, green space, inverse distance to nearest major road, and length of time at current address, AMD genetic risk score, genotyping array, and the first 10 principal components of ancestry. Model 2c further mutually adjusted for individual-level SES and area-level SES.

**Table S19. Associations of APS1 and socioeconomic status with transitions (missing values as a level)**

|  |  | **Baseline → AMD** | | **Baseline → Death** | | **AMD→OCMD** | |
| --- | --- | --- | --- | --- | --- | --- | --- |
| **APS1** | **Individual-level SES** | **Model 1** | **Model 2** | **Model 1** | **Model 2** | **Model 1** | **Model 2** |
|  |  | **HR (95% CI)** | **HR (95% CI)** | **HR (95% CI)** | **HR (95% CI)** | **HR (95% CI)** | **HR (95% CI)** |
| First tertile | High | Ref. | Ref. | Ref. | Ref. | Ref. | Ref. |
|  | Medium | 1.12 (0.95-1.33) | 1.11 (0.93-1.32) | **1.25 (1.17-1.33)** | **1.24 (1.17-1.33)** | **1.63 (1.33-1.99)** | **1.62 (1.32-1.98)** |
|  | Low | 1.13 (0.98-1.36) | 1.12 (0.96-1.34) | **1.72 (1.60-1.84)** | **1.71 (1.60-1.84)** | **1.75 (1.42-2.15)** | **1.77 (1.43-2.18)** |
| Second tertile | High | 1.15 (0.94-1.40) | 1.14 (0.94-1.39) | 0.99 (0.91-1.08) | 0.99 (0.91-1.08) | **1.33 (1.05-1.69)** | **1.29 (1.02-1.64)** |
|  | Medium | **1.24 (1.06-1.46)** | **1.23 (1.05-1.45)** | **1.29 (1.21-1.38)** | **1.29 (1.21-1.38)** | **1.71 (1.42-2.06)** | **1.67 (1.39-2.02)** |
|  | Low | **1.37 (1.16-1.62)** | **1.35 (1.14-1.60)** | **1.79 (1.67-1.92)** | **1.78 (1.66-1.91)** | **1.82 (1.50-2.20)** | **1.83 (1.51-2.23)** |
| Third tertile | High | **1.26 (1.04-1.53)** | **1.25 (1.03-1.53)** | 1.00 (0.92-1.08) | 0.99 (0.91-1.08) | **1.43 (1.13-1.82)** | **1.40 (1.10-1.79)** |
|  | Medium | **1.32 (1.11-1.56)** | **1.30 (1.10-1.54)** | **1.38 (1.29-1.48)** | **1.37 (1.28-1.47)** | **1.73 (1.41-2.11)** | **1.78 (1.46-2.19)** |
|  | Low | **1.43 (1.21-1.70)** | **1.41 (1.18-1.67)** | **1.95 (1.82-2.09)** | **1.93 (1.80-2.07)** | **1.92 (1.56-2.36)** | **1.91 (1.55-2.36)** |
| **APS1** | **Area-level SES** | **Model 1** | **Model 2** | **Model 1** | **Model 2** | **Model 1** | **Model 2** |
|  |  | **HR (95% CI)** | **HR (95% CI)** | **HR (95% CI)** | **HR (95% CI)** | **HR (95% CI)** | **HR (95% CI)** |
| First tertile | least deprived | Ref. | Ref. | Ref. | Ref. | Ref. | Ref. |
|  | moderately deprived | 1.10 (0.97-1.25) | 1.10 (0.97-1.25) | **1.06 (1.00-1.11)** | **1.06 (1.00-1.11)** | 1.02 (0.87-1.21) | 1.01 (0.83-1.24) |
|  | severely deprived | 1.13 (0.96-1.33) | 1.12 (0.95-1.32) | **1.26 (1.18-1.34)** | **1.25 (1.18-1.34)** | 1.03 (0.85-1.25) | 1.04 (0.88-1.23) |
| Second tertile | least deprived | **1.21 (1.05-1.39)** | **1.20 (1.05-1.38)** | 1.00 (0.95-1.06) | 1.00 (0.95-1.06) | 0.95 (0.80-1.11) | 0.96 (0.81-1.13) |
|  | moderately deprived | **1.22 (1.07-1.39)** | **1.22 (1.07-1.39)** | **1.08 (1.02-1.14)** | **1.08 (1.02-1.14)** | 1.13 (0.97-1.31) | 1.13 (0.97-1.31) |
|  | severely deprived | **1.24 (1.09-1.41)** | **1.23 (1.09-1.40)** | **1.39 (1.32-1.47)** | **1.38 (1.31-1.46)** | **1.20 (1.03-1.40)** | **1.19 (1.02-1.39)** |
| Third tertile | least deprived | **1.24 (1.05-1.46)** | **1.24 (1.05-1.46)** | 1.04 (0.97-1.11) | 1.03 (0.96-1.11) | 0.98 (0.84-1.13) | 0.97 (0.84-1.12) |
|  | moderately deprived | **1.31 (1.13-1.51)** | **1.30 (1.12-1.50)** | **1.15 (1.09-1.22)** | **1.15 (1.08-1.22)** | 1.16 (0.99-1.36) | **1.19 (1.01-1.40)** |
|  | severely deprived | **1.33 (1.17-1.51)** | **1.31 (1.15-1.49)** | **1.39 (1.32-1.48)** | **1.38 (1.32-1.46)** | **1.25 (1.04-1.52)** | **1.21 (1.03-1.47)** |

CI, confidence interval; HR, hazard ratio. APS1: air pollution score calculated by PCA; APS2: air pollution score calculated by weighted coefficients; AMD, age-related macular degeneration; OCMD, ocular comorbidity. Model 1 adjusted for age at recruitment, sex, region, body mass index, ethnicity, smoking status, alcohol assumption, physical activity, healthy diet score, sleep duration, noise, green space, inverse distance to nearest major road, and length of time at current address. Model 2 further adjusted for AMD genetic risk score, genotyping array, and the first 10 principal components of ancestry.

**Table S20. Associations of APS2 and socioeconomic status with transitions (missing values as a level)**

|  |  | **Baseline → AMD** | | **Baseline → Death** | | **AMD → Comorbidity** | |
| --- | --- | --- | --- | --- | --- | --- | --- |
| **APS2** | **Individual-level SES** | **Model 1** | **Model 2** | **Model 1** | **Model 2** | **Model 1** | **Model 2** |
|  |  | **HR (95% CI)** | **HR (95% CI)** | **HR (95% CI)** | **HR (95% CI)** | **HR (95% CI)** | **HR (95% CI)** |
| First tertile | High | Ref. | Ref. | Ref. | Ref. | Ref. | Ref. |
|  | Medium | **1.18 (1.01-1.39)** | **1.17 (1.00-1.38)** | **1.25 (1.17-1.33)** | **1.24 (1.17-1.33)** | **1.63 (1.32-2.00)** | **1.64 (1.33-2.02)** |
|  | Low | **1.24 (1.04-1.47)** | **1.23 (1.03-1.46)** | **1.71 (1.60-1.83)** | **1.71 (1.59-1.83)** | **1.72 (1.42-2.09)** | **1.69 (1.39-2.05)** |
| Second tertile | High | **1.31 (1.08-1.59)** | **1.30 (1.07-1.58)** | 0.97 (0.89-1.05) | 0.96 (0.89-1.05) | **1.35 (1.06-1.73)** | **1.32 (1.04-1.69)** |
|  | Medium | **1.33 (1.13-1.56)** | **1.32 (1.12-1.55)** | **1.28 (1.19-1.36)** | **1.27 (1.19-1.36)** | **1.79 (1.45-2.21)** | **1.81 (1.46-2.24)** |
|  | Low | **1.37 (1.15-1.62)** | **1.35 (1.13-1.60)** | **1.77 (1.66-1.90)** | **1.76 (1.65-1.89)** | **1.89 (1.55-2.31)** | **1.92 (1.57-2.34)** |
| Third tertile | High | **1.30 (1.07-1.59)** | **1.30 (1.07-1.58)** | 0.98 (0.90-1.07) | 0.98 (0.90-1.06) | **1.52 (1.20-1.93)** | **1.51 (1.19-1.93)** |
|  | Medium | **1.37 (1.16-1.63)** | **1.36 (1.15-1.61)** | **1.35 (1.26-1.44)** | **1.34 (1.25-1.43)** | **1.82 (1.48-2.23)** | **1.85 (1.55-2.30)** |
|  | Low | **1.54 (1.30-1.83)** | **1.51 (1.27-1.80)** | **1.91 (1.78-2.04)** | **1.89 (1.77-2.03)** | **2.09 (1.69-2.58)** | **2.06 (1.67-2.55)** |
| **APS2** | **Area-level SES** | **Model 1** | **Model 2** | **Model 1** | **Model 2** | **Model 1** | **Model 2** |
|  |  | **HR (95% CI)** | **HR (95% CI)** | **HR (95% CI)** | **HR (95% CI)** | **HR (95% CI)** | **HR (95% CI)** |
| First tertile | least deprived | Ref. | Ref. | Ref. | Ref. | Ref. | Ref. |
|  | moderately deprived | 1.10 (0.94-1.30) | 1.10 (0.93-1.29) | **1.05 (1.00-1.11)** | **1.05 (1.00-1.11)** | 1.03 (0.86-1.26) | 1.02 (0.86-1.24) |
|  | severely deprived | 1.13 (0.99-1.27) | 1.12 (0.99-1.27) | **1.28 (1.20-1.36)** | **1.27 (1.20-1.36)** | 1.05 (0.92-1.29) | 1.04 (0.88-1.25) |
| Second tertile | least deprived | **1.19 (1.05-1.36)** | **1.19 (1.04-1.36)** | 0.99 (0.93-1.05) | 0.99 (0.93-1.05) | 0.96 (0.82-1.13) | 0.95 (0.82-1.10) |
|  | moderately deprived | **1.23 (1.08-1.41)** | **1.22 (1.07-1.40)** | **1.08 (1.02-1.14)** | **1.08 (1.02-1.14)** | 1.14 (0.97-1.33) | 1.13 (0.97-1.31) |
|  | severely deprived | **1.24 (1.09-1.41)** | **1.23 (1.09-1.40)** | **1.36 (1.29-1.44)** | **1.35 (1.28-1.43)** | **1.19 (1.02-1.38)** | **1.19 (1.02-1.39)** |
| Third tertile | least deprived | **1.28 (1.11-1.47)** | **1.28 (1.11-1.47)** | 1.02 (0.95-1.09) | 1.02 (0.95-1.09) | 0.97 (0.84-1.12) | 0.97 (0.83-1.13) |
|  | moderately deprived | **1.29 (1.11-1.51)** | **1.29 (1.11-1.51)** | **1.13 (1.07-1.20)** | **1.13 (1.06-1.19)** | **1.18 (1.01-1.38)** | **1.19 (1.01-1.40)** |
|  | severely deprived | **1.33 (1.17-1.51)** | **1.31 (1.15-1.49)** | **1.36 (1.30-1.44)** | **1.35 (1.28-1.43)** | **1.22 (1.03-1.48)** | **1.21 (1.03-1.47)** |

CI, confidence interval; HR, hazard ratio. APS1: air pollution score calculated by PCA; APS2: air pollution score calculated by weighted coefficients; AMD, age-related macular degeneration; OCMD, ocular comorbidity. Model 1 adjusted for age at recruitment, sex, region, body mass index, ethnicity, smoking status, alcohol assumption, physical activity, healthy diet score, sleep duration, noise, green space, inverse distance to nearest major road, and length of time at current address. Model 2 further adjusted for AMD genetic risk score, genotyping array, and the first 10 principal components of ancestry.

**Table S21. Associations of APS1 and socioeconomic status with transitions (remove cases and deaths within the first 2 years, N=318860)**

|  |  | **Baseline → AMD** | | **Baseline → Death** | | **AMD→OCMD** | |
| --- | --- | --- | --- | --- | --- | --- | --- |
| **APS1** | **Individual-level SES** | **Model 1** | **Model 2** | **Model 1** | **Model 2** | **Model 1** | **Model 2** |
|  |  | **HR (95% CI)** | **HR (95% CI)** | **HR (95% CI)** | **HR (95% CI)** | **HR (95% CI)** | **HR (95% CI)** |
| First tertile | High | Ref. | Ref. | Ref. | Ref. | Ref. | Ref. |
|  | Medium | 1.11 (0.93-1.32) | 1.10 (0.93-1.32) | **1.25 (1.17-1.34)** | **1.25 (1.17-1.34)** | **1.73 (1.41-2.13)** | **1.72 (1.40-2.13)** |
|  | Low | 1.12 (0.96-1.34) | 1.11 (0.96-1.31) | **1.72 (1.60-1.85)** | **1.72 (1.60-1.85)** | **1.77 (1.44-2.18)** | **1.76 (1.43-2.18)** |
| Second tertile | High | 1.13 (0.94-1.36) | 1.12 (0.92-1.37) | 1.01 (0.93-1.10) | 1.01 (0.93-1.10) | **1.39 (1.09-1.78)** | **1.34 (1.05-1.72)** |
|  | Medium | **1.23 (1.05-1.45)** | **1.22 (1.04-1.44)** | **1.30 (1.21-1.39)** | **1.30 (1.21-1.39)** | **1.86 (1.54-2.24)** | **1.83 (1.51-2.21)** |
|  | Low | **1.36 (1.14-1.61)** | **1.34 (1.13-1.59)** | **1.78 (1.66-1.91)** | **1.77 (1.65-1.90)** | **1.94 (1.59-2.36)** | **1.95 (1.60-2.37)** |
| Third tertile | High | **1.24 (1.02-1.52)** | **1.23 (1.01-1.51)** | 0.99 (0.91-1.08) | 0.99 (0.91-1.08) | **1.51 (1.18-1.92)** | **1.48 (1.16-1.89)** |
|  | Medium | **1.31 (1.11-1.52)** | **1.30 (1.10-1.54)** | **1.38 (1.29-1.49)** | **1.37 (1.28-1.48)** | **1.92 (1.56-2.37)** | **1.93 (1.56-2.39)** |
|  | Low | **1.39 (1.17-1.66)** | **1.36 (1.14-1.63)** | **1.95 (1.81-2.09)** | **1.93 (1.79-2.07)** | **1.96 (1.59-2.43)** | **1.97 (1.59-2.45)** |
| **APS1** | **Area-level SES** | **Model 1** | **Model 2** | **Model 1** | **Model 2** | **Model 1** | **Model 2** |
|  |  | **HR (95% CI)** | **HR (95% CI)** | **HR (95% CI)** | **HR (95% CI)** | **HR (95% CI)** | **HR (95% CI)** |
| First tertile | least deprived | Ref. | Ref. | Ref. | Ref. | Ref. | Ref. |
|  | moderately deprived | 1.09 (0.96-1.24) | 1.09 (0.96-1.24) | **1.06 (1.00-1.11)** | **1.06 (1.00-1.12)** | 1.01 (0.86-1.20) | 1.01 (0.86-1.20) |
|  | severely deprived | 1.11 (0.93-1.31) | 1.10 (0.93-1.30) | **1.25 (1.17-1.34)** | **1.25 (1.17-1.34)** | 1.02 (0.84-1.24) | 1.02 (0.84-1.24) |
| Second tertile | least deprived | **1.18 (1.02-1.36)** | **1.18 (1.04-1.35)** | 1.01 (0.95-1.07) | 1.00 (0.95-1.06) | 0.95 (0.80-1.11) | 0.95 (0.80-1.11) |
|  | moderately deprived | **1.20 (1.05-1.38)** | **1.20 (1.04-1.38)** | **1.08 (1.03-1.15)** | **1.08 (1.02-1.14)** | 1.10 (0.94-1.29) | 1.10 (0.94-1.29) |
|  | severely deprived | **1.23 (1.08-1.40)** | **1.22 (1.07-1.39)** | **1.38 (1.30-1.46)** | **1.37 (1.29-1.45)** | 1.17 (0.99-1.37) | 1.16 (0.98-1.37) |
| Third tertile | least deprived | **1.19 (1.03-1.41)** | **1.19 (1.01-1.41)** | 1.04 (0.97-1.12) | 1.04 (0.96-1.11) | 0.98 (0.84-1.13) | 0.98 (0.84-1.13) |
|  | moderately deprived | **1.29 (1.11-1.49)** | **1.27 (1.10-1.48)** | **1.14 (1.07-1.21)** | **1.13 (1.06-1.20)** | 1.13 (0.93-1.38) | 1.12 (0.92-1.37) |
|  | severely deprived | **1.30 (1.14-1.49)** | **1.28 (1.12-1.48)** | **1.38 (1.30-1.46)** | **1.36 (1.29-1.44)** | **1.23 (1.06-1.44)** | **1.22 (1.05-1.43)** |

CI, confidence interval; HR, hazard ratio. APS1: air pollution score calculated by PCA; APS2: air pollution score calculated by weighted coefficients; AMD, age-related macular degeneration; OCMD, ocular comorbidity. Model 1 adjusted for age at recruitment, sex, region, body mass index, ethnicity, smoking status, alcohol assumption, physical activity, healthy diet score, sleep duration, noise, green space, inverse distance to nearest major road, and length of time at current address. Model 2 further adjusted for AMD genetic risk score, genotyping array, and the first 10 principal components of ancestry.

**Table S22. Associations of APS2 and socioeconomic status with transitions (remove cases and deaths within the first 2 years, N=318860)**

|  |  | **Baseline → AMD** | | **Baseline → Death** | | **AMD→OCMD** | |
| --- | --- | --- | --- | --- | --- | --- | --- |
| **APS2** | **Individual-level SES** | **Model 1** | **Model 2** | **Model 1** | **Model 2** | **Model 1** | **Model 2** |
|  |  | **HR (95% CI)** | **HR (95% CI)** | **HR (95% CI)** | **HR (95% CI)** | **HR (95% CI)** | **HR (95% CI)** |
| First tertile | High | Ref. | Ref. | Ref. | Ref. | Ref. | Ref. |
|  | Medium | **1.19 (1.02-1.40)** | **1.18 (1.01-1.39)** | **1.25 (1.17-1.34)** | **1.25 (1.17-1.34)** | **1.62 (1.31-2.00)** | **1.61 (1.30-2.00)** |
|  | Low | **1.23 (1.03-1.48)** | **1.23 (1.03-1.47)** | **1.72 (1.60-1.84)** | **1.71 (1.59-1.84)** | **1.71 (1.41-2.09)** | **1.70 (1.40-2.08)** |
| Second tertile | High | **1.30 (1.07-1.59)** | **1.30 (1.06-1.58)** | 0.98 (0.90-1.07) | 0.98 (0.90-1.07) | **1.42 (1.11-1.82)** | **1.40 (1.08-1.84)** |
|  | Medium | **1.32 (1.12-1.56)** | **1.31 (1.11-1.55)** | **1.29 (1.20-1.38)** | **1.28 (1.20-1.36)** | **1.78 (1.44-2.21)** | **1.80 (1.46-2.27)** |
|  | Low | **1.38 (1.16-1.64)** | **1.36 (1.14-1.62)** | **1.77 (1.65-1.90)** | **1.76 (1.64-1.89)** | **1.85 (1.52-2.25)** | **1.86 (1.53-2.29)** |
| Third tertile | High | **1.30 (1.06-1.59)** | **1.29 (1.06-1.58)** | 0.98 (0.90-1.07) | 0.98 (0.90-1.07) | **1.57 (1.23-2.01)** | **1.58 (1.24-2.01)** |
|  | Medium | **1.39 (1.17-1.65)** | **1.38 (1.16-1.64)** | **1.35 (1.26-1.45)** | **1.34 (1.25-1.44)** | **1.81 (1.46-2.23)** | **1.81 (1.46-2.23)** |
|  | Low | **1.50 (1.26-1.79)** | **1.47 (1.23-1.76)** | **1.91 (1.78-2.05)** | **1.89 (1.76-2.03)** | **2.08 (1.68-2.58)** | **2.06 (1.66-2.56)** |
| **APS2** | **Area-level SES** | **Model 1** | **Model 2** | **Model 1** | **Model 2** | **Model 1** | **Model 2** |
|  |  | **HR (95% CI)** | **HR (95% CI)** | **HR (95% CI)** | **HR (95% CI)** | **HR (95% CI)** | **HR (95% CI)** |
| First tertile | least deprived | Ref. | Ref. | Ref. | Ref. | Ref. | Ref. |
|  | moderately deprived | 1.08 (0.92-1.28) | 1.08 (0.91-1.28) | **1.06 (1.00-1.11)** | **1.06 (1.00-1.11)** | 1.03 (0.86-1.26) | 1.02 (0.85-1.25) |
|  | severely deprived | 1.12 (0.99-1.27) | 1.12 (0.98-1.27) | **1.27 (1.19-1.35)** | **1.27 (1.19-1.35)** | 1.05 (0.92-1.29) | 1.04 (0.91-1.29) |
| Second tertile | least deprived | **1.17 (1.02-1.34)** | **1.17 (1.02-1.33)** | 0.99 (0.94-1.05) | 0.99 (0.94-1.05) | 0.96 (0.82-1.13) | 0.96 (0.82-1.13) |
|  | moderately deprived | **1.23 (1.08-1.40)** | **1.23 (1.08-1.40)** | **1.08 (1.02-1.14)** | **1.08 (1.02-1.14)** | 1.12 (0.95-1.30) | 1.11 (0.94-1.30) |
|  | severely deprived | **1.24 (1.08-1.42)** | **1.23 (1.08-1.41)** | **1.35 (1.28-1.43)** | **1.35 (1.27-1.42)** | 1.17 (0.99-1.32) | 1.16 (0.98-1.32) |
| Third tertile | least deprived | **1.26 (1.08-1.47)** | **1.26 (1.08-1.47)** | 1.03 (0.96-1.10) | 1.02 (0.96-1.10) | 0.97 (0.83-1.12) | 0.97 (0.83-1.13) |
|  | moderately deprived | **1.27 (1.10-1.47)** | **1.27 (1.10-1.47)** | **1.12 (1.05-1.19)** | **1.11 (1.05-1.18)** | **1.17 (1.00-1.36)** | 1.16 (0.99-1.35) |
|  | severely deprived | **1.31 (1.14-1.49)** | **1.31 (1.14-1.49)** | **1.37 (1.29-1.44)** | **1.35 (1.28-1.43)** | **1.22 (1.02-1.48)** | **1.21 (1.01-1.47)** |

CI, confidence interval; HR, hazard ratio. APS1: air pollution score calculated by PCA; APS2: air pollution score calculated by weighted coefficients; AMD, age-related macular degeneration; OCMD, ocular comorbidity. Model 1 adjusted for age at recruitment, sex, region, body mass index, ethnicity, smoking status, alcohol assumption, physical activity, healthy diet score, sleep duration, noise, green space, inverse distance to nearest major road, and length of time at current address. Model 2 further adjusted for AMD genetic risk score, genotyping array, and the first 10 principal components of ancestry.

**Table S23. Associations of APS1 and socioeconomic status with transitions (remove participants who experienced different states on the same day, N=320311)**

|  |  | **Baseline → AMD** | | **Baseline → Death** | | **AMD→OCMD** | |
| --- | --- | --- | --- | --- | --- | --- | --- |
| **APS1** | **Individual-level SES** | **Model 1** | **Model 2** | **Model 1** | **Model 2** | **Model 1** | **Model 2** |
|  |  | **HR (95% CI)** | **HR (95% CI)** | **HR (95% CI)** | **HR (95% CI)** | **HR (95% CI)** | **HR (95% CI)** |
| First tertile | High | Ref. | Ref. | Ref. | Ref. | Ref. | Ref. |
|  | Medium | 1.11 (0.93-1.32) | 1.10 (0.93-1.32) | **1.25 (1.17-1.34)** | **1.25 (1.17-1.34)** | **1.73 (1.41-2.13)** | **1.72 (1.40-2.13)** |
|  | Low | 1.12 (0.96-1.34) | 1.12 (0.96-1.31) | **1.72 (1.60-1.85)** | **1.72 (1.60-1.85)** | **1.77 (1.44-2.18)** | **1.76 (1.43-2.18)** |
| Second tertile | High | 1.13 (0.96-1.36) | 1.12 (0.92-1.37) | 1.01 (0.93-1.10) | 1.01 (0.93-1.10) | **1.39 (1.09-1.78)** | **1.34 (1.05-1.72)** |
|  | Medium | **1.23 (1.05-1.45)** | **1.22 (1.04-1.44)** | **1.30 (1.21-1.39)** | **1.30 (1.21-1.39)** | **1.86 (1.54-2.24)** | **1.83 (1.51-2.21)** |
|  | Low | **1.36 (1.14-1.61)** | **1.34 (1.13-1.59)** | **1.78 (1.66-1.91)** | **1.77 (1.65-1.90)** | **1.94 (1.59-2.36)** | **1.95 (1.60-2.37)** |
| Third tertile | High | **1.24 (1.02-1.52)** | **1.23 (1.01-1.51)** | 0.99 (0.91-1.08) | 0.99 (0.91-1.08) | **1.51 (1.18-1.92)** | **1.48 (1.16-1.89)** |
|  | Medium | **1.31 (1.11-1.52)** | **1.30 (1.10-1.54)** | **1.38 (1.29-1.49)** | **1.37 (1.28-1.48)** | **1.92 (1.56-2.37)** | **1.93 (1.56-2.39)** |
|  | Low | **1.39 (1.17-1.66)** | **1.36 (1.14-1.63)** | **1.95 (1.81-2.09)** | **1.93 (1.79-2.07)** | **1.96 (1.59-2.43)** | **1.97 (1.59-2.45)** |
| **APS1** | **Area-level SES** | **Model 1** | **Model 2** | **Model 1** | **Model 2** | **Model 1** | **Model 2** |
|  |  | **HR (95% CI)** | **HR (95% CI)** | **HR (95% CI)** | **HR (95% CI)** | **HR (95% CI)** | **HR (95% CI)** |
| First tertile | least deprived | Ref. | Ref. | Ref. | Ref. | Ref. | Ref. |
|  | moderately deprived | 1.09 (0.96-1.24) | 1.09 (0.96-1.24) | **1.06 (1.00-1.11)** | **1.06 (1.00-1.12)** | 1.01 (0.86-1.20) | 1.01 (0.86-1.20) |
|  | severely deprived | 1.11 (0.93-1.31) | 1.10 (0.93-1.30) | **1.25 (1.17-1.34)** | **1.25 (1.17-1.34)** | 1.02 (0.84-1.24) | 1.02 (0.84-1.24) |
| Second tertile | least deprived | **1.18 (1.02-1.36)** | **1.18 (1.04-1.35)** | 1.01 (0.95-1.07) | 1.00 (0.95-1.06) | 0.95 (0.80-1.11) | 0.95 (0.80-1.11) |
|  | moderately deprived | **1.20 (1.05-1.38)** | **1.20 (1.04-1.38)** | **1.08 (1.03-1.15)** | **1.08 (1.02-1.14)** | 1.10 (0.94-1.29) | 1.10 (0.94-1.29) |
|  | severely deprived | **1.23 (1.08-1.40)** | **1.22 (1.07-1.39)** | **1.38 (1.30-1.46)** | **1.37 (1.29-1.45)** | 1.17 (0.99-1.37) | 1.16 (0.98-1.37) |
| Third tertile | least deprived | **1.19 (1.03-1.41)** | **1.19 (1.01-1.41)** | 1.04 (0.97-1.12) | 1.04 (0.96-1.11) | 0.98 (0.84-1.13) | 0.98 (0.84-1.13) |
|  | moderately deprived | **1.29 (1.11-1.49)** | **1.27 (1.10-1.48)** | **1.14 (1.07-1.21)** | **1.13 (1.06-1.20)** | 1.13 (0.93-1.38) | 1.12 (0.92-1.37) |
|  | severely deprived | **1.30 (1.14-1.49)** | **1.28 (1.12-1.48)** | **1.38 (1.30-1.46)** | **1.36 (1.29-1.44)** | **1.23 (1.06-1.44)** | **1.22 (1.05-1.43)** |

CI, confidence interval; HR, hazard ratio. APS1: air pollution score calculated by PCA; APS2: air pollution score calculated by weighted coefficients; AMD, age-related macular degeneration; OCMD, ocular comorbidity. Model 1 adjusted for age at recruitment, sex, region, body mass index, ethnicity, smoking status, alcohol assumption, physical activity, healthy diet score, sleep duration, noise, green space, inverse distance to nearest major road, and length of time at current address. Model 2 further adjusted for AMD genetic risk score, genotyping array, and the first 10 principal components of ancestry.

**Table S24. Associations of APS2 and socioeconomic status with transitions (remove participants who experienced different states on the same day, N=320311)**

|  |  | **Baseline → AMD** | | **Baseline → Death** | | **AMD→OCMD** | |
| --- | --- | --- | --- | --- | --- | --- | --- |
| **APS2** | **Individual-level SES** | **Model 1** | **Model 2** | **Model 1** | **Model 2** | **Model 1** | **Model 2** |
|  |  | **HR (95% CI)** | **HR (95% CI)** | **HR (95% CI)** | **HR (95% CI)** | **HR (95% CI)** | **HR (95% CI)** |
| First tertile | High | Ref. | Ref. | Ref. | Ref. | Ref. | Ref. |
|  | Medium | **1.19 (1.02-1.40)** | **1.18 (1.01-1.39)** | **1.25 (1.17-1.34)** | **1.25 (1.17-1.34)** | **1.62 (1.31-2.00)** | **1.61 (1.30-2.00)** |
|  | Low | **1.23 (1.03-1.48)** | **1.23 (1.03-1.47)** | **1.72 (1.60-1.84)** | **1.71 (1.59-1.84)** | **1.71 (1.41-2.09)** | **1.70 (1.40-2.08)** |
| Second tertile | High | **1.30 (1.07-1.59)** | **1.30 (1.06-1.58)** | 0.98 (0.90-1.07) | 0.98 (0.90-1.07) | **1.42 (1.11-1.82)** | **1.40 (1.08-1.84)** |
|  | Medium | **1.32 (1.12-1.56)** | **1.31 (1.11-1.55)** | **1.29 (1.20-1.38)** | **1.28 (1.20-1.36)** | **1.78 (1.44-2.21)** | **1.80 (1.46-2.27)** |
|  | Low | **1.38 (1.16-1.64)** | **1.36 (1.14-1.62)** | **1.77 (1.65-1.90)** | **1.76 (1.64-1.89)** | **1.85 (1.52-2.25)** | **1.86 (1.53-2.29)** |
| Third tertile | High | **1.30 (1.06-1.59)** | **1.29 (1.06-1.58)** | 0.98 (0.90-1.07) | 0.98 (0.90-1.07) | **1.57 (1.23-2.01)** | **1.58 (1.24-2.01)** |
|  | Medium | **1.39 (1.17-1.65)** | **1.38 (1.16-1.64)** | **1.35 (1.26-1.45)** | **1.34 (1.25-1.44)** | **1.81 (1.46-2.23)** | **1.81 (1.46-2.23)** |
|  | Low | **1.50 (1.26-1.79)** | **1.47 (1.23-1.76)** | **1.91 (1.78-2.05)** | **1.89 (1.76-2.03)** | **2.08 (1.68-2.58)** | **2.06 (1.66-2.56)** |
| **APS2** | **Area-level SES** | **Model 1** | **Model 2** | **Model 1** | **Model 2** | **Model 1** | **Model 2** |
|  |  | **HR (95% CI)** | **HR (95% CI)** | **HR (95% CI)** | **HR (95% CI)** | **HR (95% CI)** | **HR (95% CI)** |
| First tertile | least deprived | Ref. | Ref. | Ref. | Ref. | Ref. | Ref. |
|  | moderately deprived | 1.08 (0.92-1.28) | 1.08 (0.91-1.28) | **1.06 (1.00-1.11)** | **1.06 (1.00-1.11)** | 1.03 (0.86-1.26) | 1.02 (0.85-1.25) |
|  | severely deprived | 1.12 (0.99-1.27) | 1.12 (0.98-1.27) | **1.27 (1.19-1.35)** | **1.27 (1.19-1.35)** | 1.05 (0.92-1.29) | 1.04 (0.91-1.29) |
| Second tertile | least deprived | **1.17 (1.02-1.34)** | **1.17 (1.02-1.33)** | 0.99 (0.94-1.05) | 0.99 (0.94-1.05) | 0.96 (0.82-1.13) | 0.96 (0.82-1.13) |
|  | moderately deprived | **1.23 (1.08-1.40)** | **1.23 (1.08-1.40)** | **1.08 (1.02-1.14)** | **1.08 (1.02-1.14)** | 1.12 (0.95-1.30) | 1.11 (0.94-1.30) |
|  | severely deprived | **1.24 (1.08-1.42)** | **1.23 (1.08-1.41)** | **1.35 (1.28-1.43)** | **1.35 (1.27-1.42)** | 1.17 (0.99-1.32) | 1.16 (0.98-1.32) |
| Third tertile | least deprived | **1.26 (1.08-1.47)** | **1.26 (1.08-1.47)** | 1.03 (0.96-1.10) | 1.02 (0.96-1.10) | 0.97 (0.83-1.12) | 0.97 (0.83-1.13) |
|  | moderately deprived | **1.27 (1.10-1.47)** | **1.27 (1.10-1.47)** | **1.12 (1.05-1.19)** | **1.11 (1.05-1.18)** | **1.17 (1.00-1.36)** | 1.16 (0.99-1.35) |
|  | severely deprived | **1.31 (1.14-1.49)** | **1.31 (1.14-1.49)** | **1.37 (1.29-1.44)** | **1.35 (1.28-1.43)** | **1.22 (1.02-1.48)** | **1.21 (1.01-1.47)** |

CI, confidence interval; HR, hazard ratio. APS1: air pollution score calculated by PCA; APS2: air pollution score calculated by weighted coefficients; AMD, age-related macular degeneration; OCMD, ocular comorbidity. Model 1 adjusted for age at recruitment, sex, region, body mass index, ethnicity, smoking status, alcohol assumption, physical activity, healthy diet score, sleep duration, noise, green space, inverse distance to nearest major road, and length of time at current address. Model 2 further adjusted for AMD genetic risk score, genotyping array, and the first 10 principal components of ancestry.

**Table S25. Analyses on additive and multiplicative interactions between APS1 and SES with incident AMD**

|  | **First tertile** | **Second/third tertile** | **Multiplicative interaction** | **Additive interaction** | | |
| --- | --- | --- | --- | --- | --- | --- |
|  | **HR (95%CI)** | **HR (95%CI)** | **HR (95%CI)** | **RERI** | **AP** | **S** |
| **Individual-level SES** |  | | | | | |
| high | Ref. | 1.19 (1.01, 1.41) | 0.98 (0.82, 1.17) | 0.00 (-0.20, 0.19) | 0.00 (-0.15, 0.15) | 0.99 (0.52, 1.90) |
| medium /low | 1.11 (0.96, 1.28) | 1.30 (1.12, 1.50) |  |  |  |  |
| **Area-level SES** |  | | | | | |
| least deprived | Ref. | 1.22 (1.08, 1.37) | 0.93 (0.81, 1.07) | -0.07 (-0.24, 0.09) | -0.06 (-0.19, 0.07) | 0.78 (0.48, 1.27) |
| moderately /severely deprived | 1.11 (0.99, 1.24) | 1.25 (1.13, 1.39) |  |  |  |  |

SES, socioeconomic status; APS1: air pollution score calculated by PCA; APS2: air pollution score calculated by weighted coefficients; RERI, relative excess risk due to interaction; AP, attributable proportion due to interaction; S, the synergy index; HR, hazard ratio; CI, confidence interval. Model adjusted for sex, region, body mass index, ethnicity, smoking status, alcohol assumption, physical activity, healthy diet score, sleep duration, noise, green space, inverse distance to nearest major road, length of time at current address, AMD genetic risk score, genotyping array, and the first 10 principal components of ancestry.

**Table S26. Analyses on additive and multiplicative interactions between APS2 and SES with incident AMD**

|  | **First tertile** | **Second/third tertile** | **Multiplicative interaction** | **Additive interaction** | | |
| --- | --- | --- | --- | --- | --- | --- |
|  | **HR (95%CI)** | **HR (95%CI)** | **HR (95%CI)** | **RERI** | **AP** | **S** |
| **Individual-level SES** |  | | | | | |
| high | Ref. | 1.29 (1.09, 1.53) | 0.89 (0.74, 1.07) | -0.11 (-0.33, 0.10) | -0.08 (-0.24, 0.07) | 0.76 (0.51, 1.13) |
| medium /low | 1.18 (1.02, 1.38) | 1.36 (1.17, 1.58) |  |  |  |  |
| **Area-level SES** |  | | | | | |
| least deprived | Ref. | 1.22 (1.09, 1.37) | 0.92 (0.80, 1.06) | -0.08 (-0.24, 0.08) | -0.06 (-0.19, 0.07) | 0.76 (0.48, 1.22) |
| moderately /severely deprived | 1.11 (0.99, 1.25) | 1.26 (1.13, 1.40) |  |  |  |  |

SES, socioeconomic status; APS1: air pollution score calculated by PCA; APS2: air pollution score calculated by weighted coefficients; RERI, relative excess risk due to interaction; AP, attributable proportion due to interaction; S, the synergy index; HR, hazard ratio; CI, confidence interval. Model adjusted for sex, region, body mass index, ethnicity, smoking status, alcohol assumption, physical activity, healthy diet score, sleep duration, noise, green space, inverse distance to nearest major road, length of time at current address, AMD genetic risk score, genotyping array, and the first 10 principal components of ancestry.

**Fig. S1. Flowchart of participant enrolment**

502,193 participants recruited to the UK Biobank at the baseline

51,773 participants with age <45 years were excluded

450,420 participants with age ≥45 years

105,552 participants were excluded:

- 36,828 without complete data of air pollutants
- 68,724 without complete data of socioeconomic variables

344,868 participants with complete exposure and outcome data

320,565 participants included in the main analysis

24,303 participants were excluded:

- 23,029 with prevalent AMD, glaucoma, cataract at baseline
- 1,274 with incorrect temporal sequencing

**Fig. S2. Dose-response associations between air pollutants and incident AMD**

**
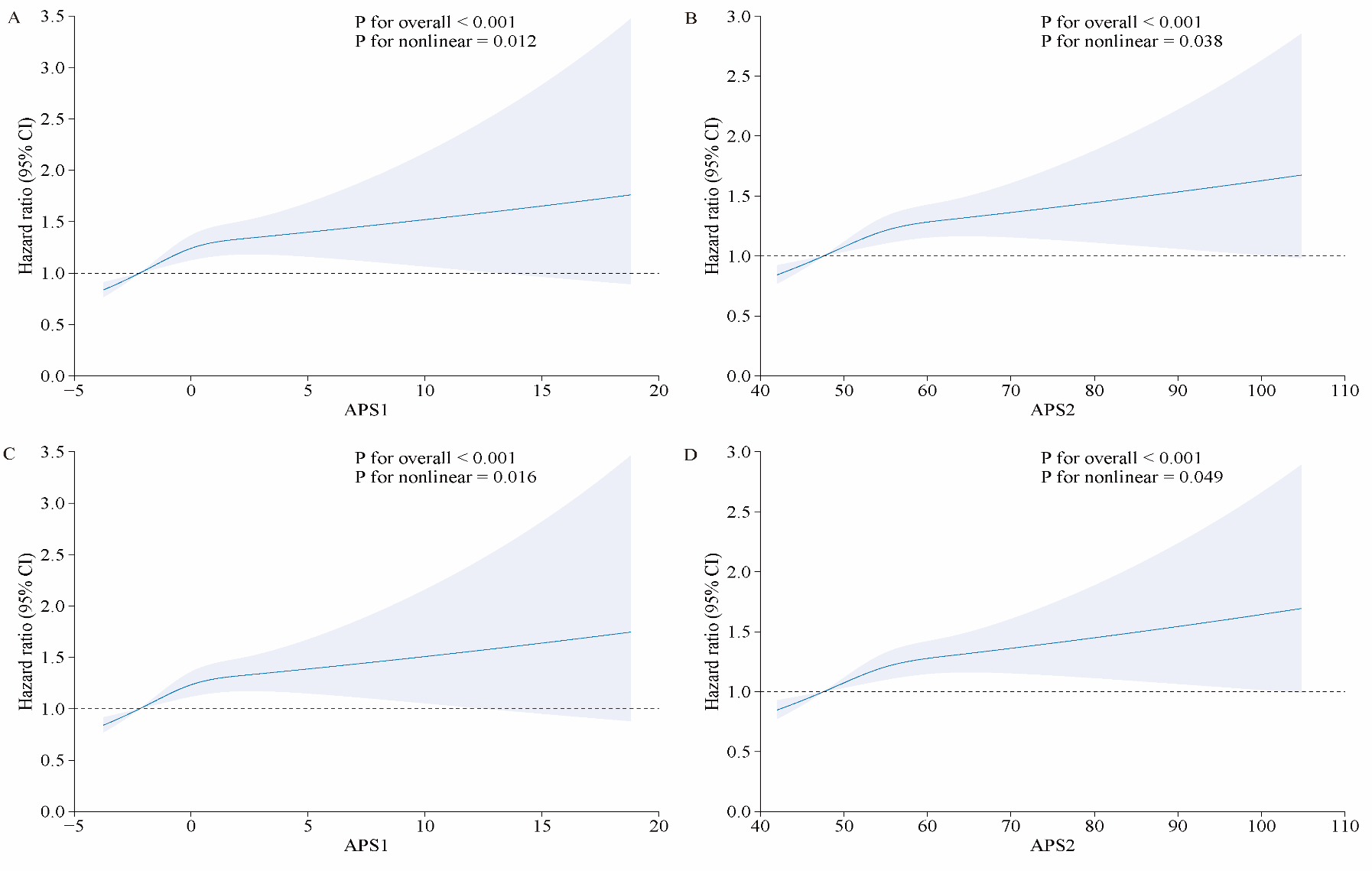
**

APS1: air pollution score calculated by PCA; APS2: air pollution score calculated by weighted coefficients; RCS: restricted cubic splines; HR, hazard ratio; CI, confidence interval. Plots A and B adjusted for age at recruitment, sex, region, body mass index, ethnicity, smoking status, alcohol assumption, physical activity, healthy diet score, sleep duration, noise, green space, inverse distance to nearest major road, and length of time at current address. Plots C and D further adjusted for AMD genetic risk score, genotyping array, and the first 10 principal components of ancestry. Solid lines indicate HRs, and shadow shape indicate 95% CIs. The RCS model was conducted with 4 knots at the 5th, 35th, 65th, 95th percentiles of each APS (reference is the 5th percentile).

**Fig. S3. Dose-response associations between air pollutants and incident OCMD after AMD diagnosis**

**
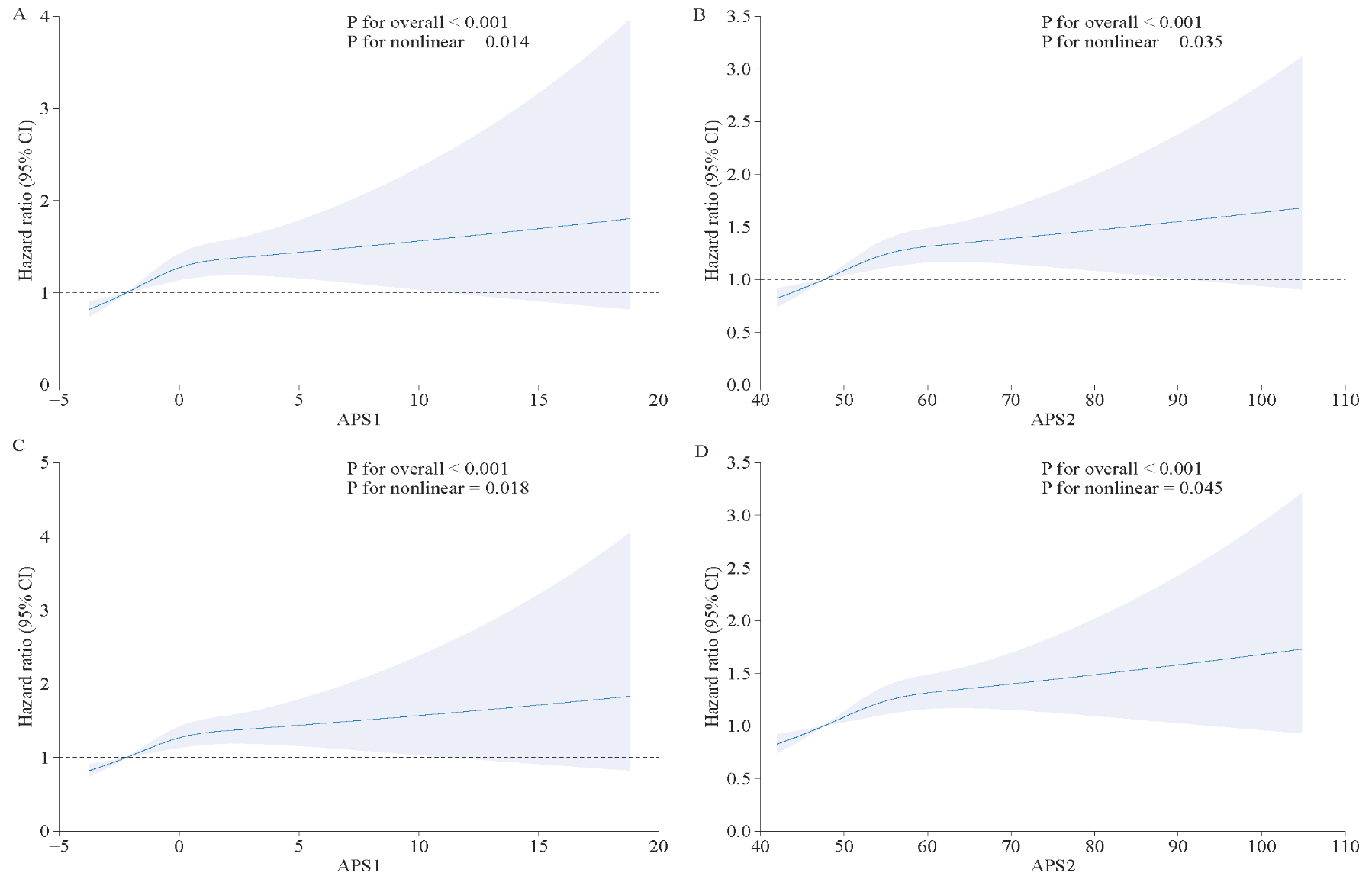
**

APS1: air pollution score calculated by PCA; APS2: air pollution score calculated by weighted coefficients; RCS: restricted cubic splines; HR, hazard ratio; CI, confidence interval. Plots A and B adjusted for age at recruitment, sex, region, body mass index, ethnicity, smoking status, alcohol assumption, physical activity, healthy diet score, sleep duration, noise, green space, inverse distance to nearest major road, and length of time at current address. Plots C and D further adjusted for AMD genetic risk score, genotyping array, and the first 10 principal components of ancestry. Solid lines indicate HRs, and shadow shape indicate 95% CIs. The RCS model was conducted with 4 knots at the 5th, 35th, 65th, 95th percentiles of each APS (reference is the 5th percentile).

**Fig. S4. Dose-response associations between air pollutants and all-cause mortality**

**
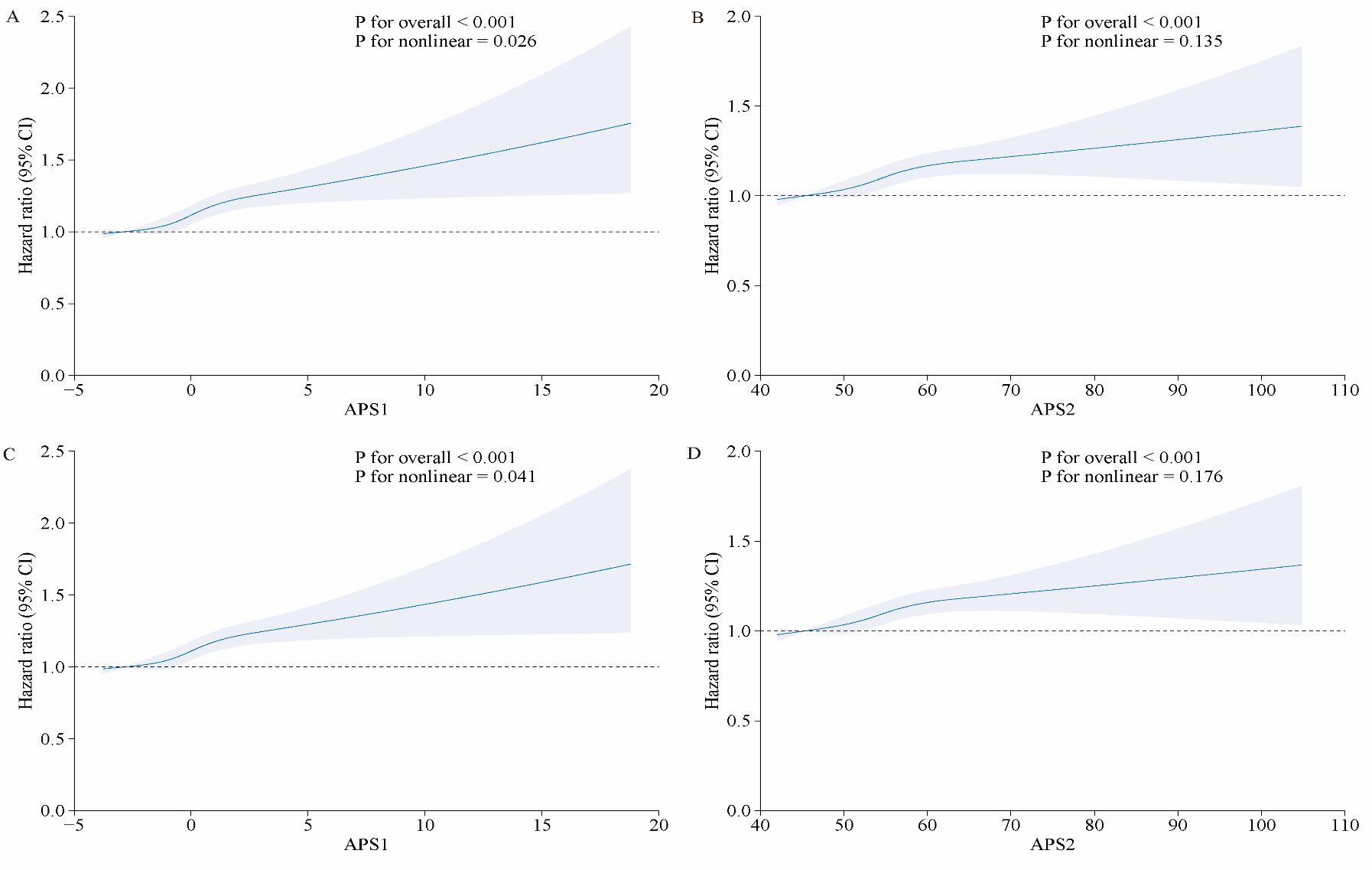
**

APS1: air pollution score calculated by PCA; APS2: air pollution score calculated by weighted coefficients; RCS: restricted cubic splines; HR, hazard ratio; CI, confidence interval. Plots A and B adjusted for age at recruitment, sex, region, body mass index, ethnicity, smoking status, alcohol assumption, physical activity, healthy diet score, sleep duration, noise, green space, inverse distance to nearest major road, and length of time at current address. Plots C and D further adjusted for AMD genetic risk score, genotyping array, and the first 10 principal components of ancestry. Solid lines indicate HRs, and shadow shape indicate 95% CIs. The RCS model was conducted with 4 knots at the 5th, 35th, 65th, 95th percentiles of each APS (reference is the 5th percentile).

**Fig. S5. Joint associations of APS1 and socioeconomic status with incident OCMD after AMD diagnosis**

**
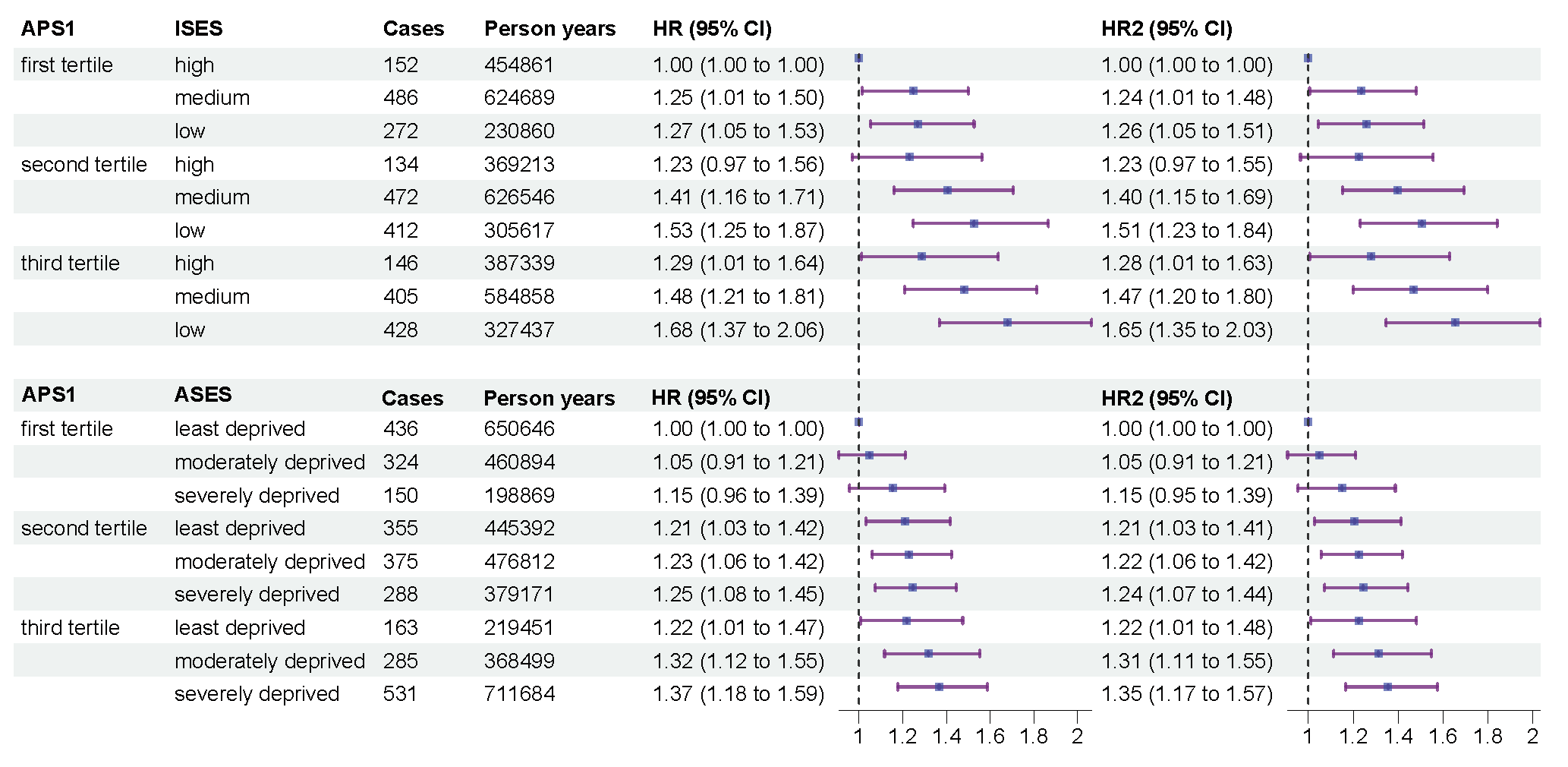
**

CI, confidence interval; HR, hazard ratio. APS1: air pollution score calculated by PCA; APS2: air pollution score calculated by weighted coefficients; ISES, individual-level SES; ASES, area-level SES; AMD, age-related macular degeneration. Model 1 adjusted for age at recruitment, sex, region, body mass index, ethnicity, smoking status, alcohol assumption, physical activity, healthy diet score, sleep duration, noise, green space, inverse distance to nearest major road, and length of time at current address. Model 2 further adjusted for AMD genetic risk score, genotyping array, and the first 10 principal components of ancestry.

**Fig. S6. Joint associations of APS2 and socioeconomic status with incident OCMD after AMD diagnosis**


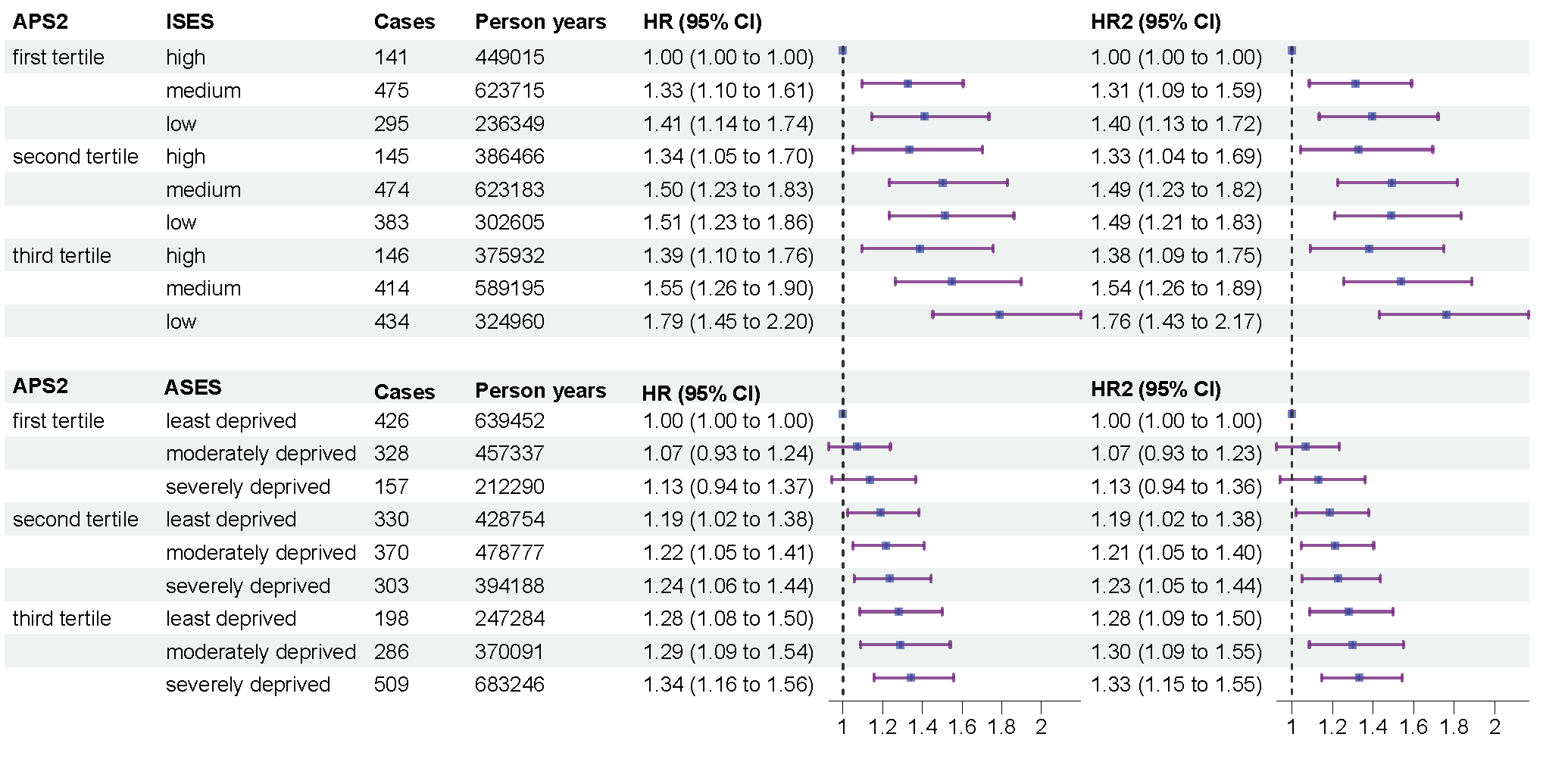
CI, confidence interval; HR, hazard ratio. APS1: air pollution score calculated by PCA; APS2: air pollution score calculated by weighted coefficients; ISES, individual-level SES; ASES, area-level SES; AMD, age-related macular degeneration. Model 1 adjusted for age at recruitment, sex, region, body mass index, ethnicity, smoking status, alcohol assumption, physical activity, healthy diet score, sleep duration, noise, green space, inverse distance to nearest major road, and length of time at current address. Model 2 further adjusted for AMD genetic risk score, genotyping array, and the first 10 principal components of ancestry.

**Fig. S7. Joint associations of APS1 and socioeconomic status with all-cause mortality**

**
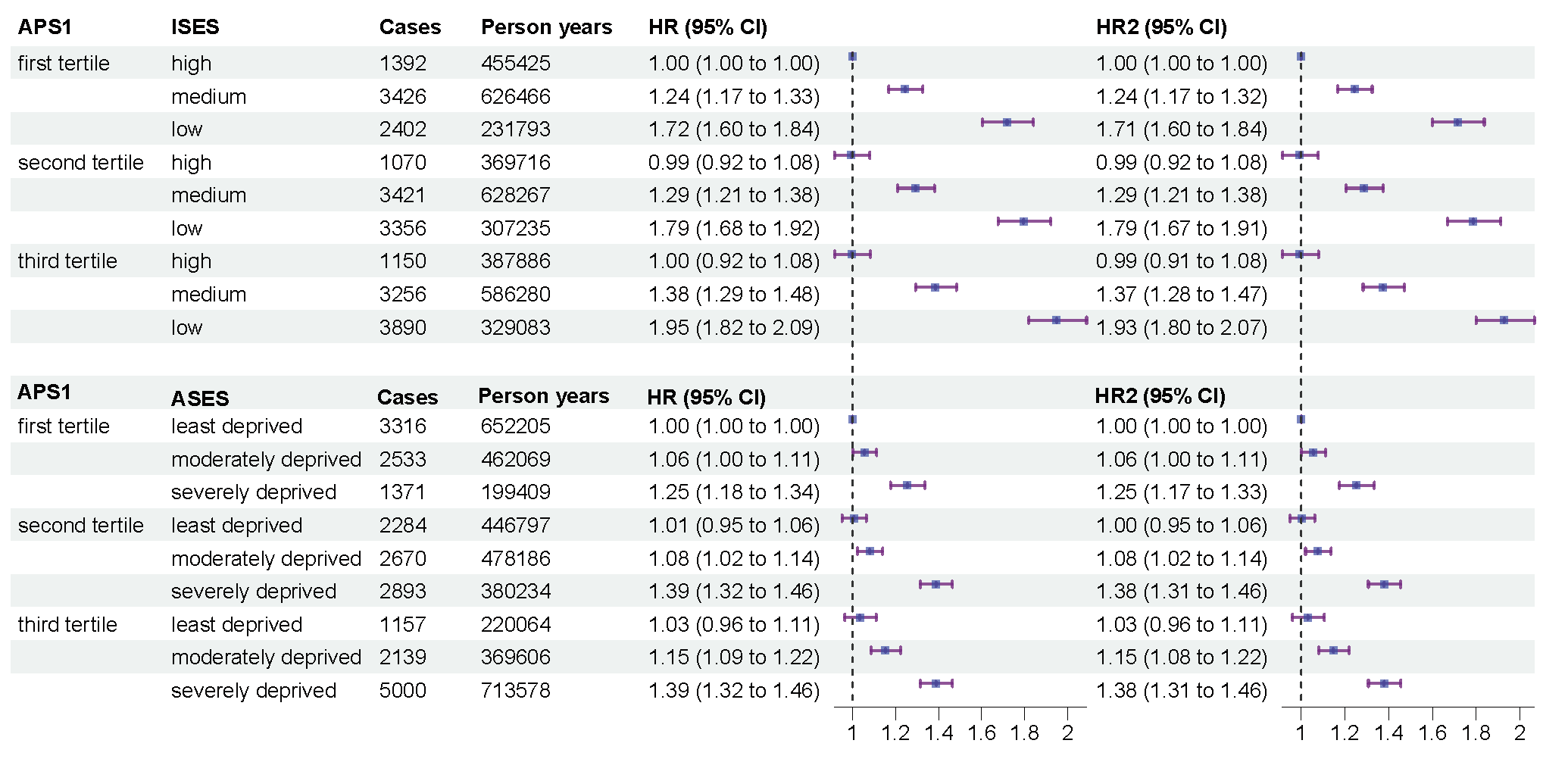
**

CI, confidence interval; HR, hazard ratio. APS1: air pollution score calculated by PCA; APS2: air pollution score calculated by weighted coefficients; ISES, individual-level SES; ASES, area-level SES; AMD, age-related macular degeneration. Model 1 adjusted for age at recruitment, sex, region, body mass index, ethnicity, smoking status, alcohol assumption, physical activity, healthy diet score, sleep duration, noise, green space, inverse distance to nearest major road, and length of time at current address. Model 2 further adjusted for AMD genetic risk score, genotyping array, and the first 10 principal components of ancestry.

**Fig. S8. Joint associations of APS2 and socioeconomic status with all-cause mortality**


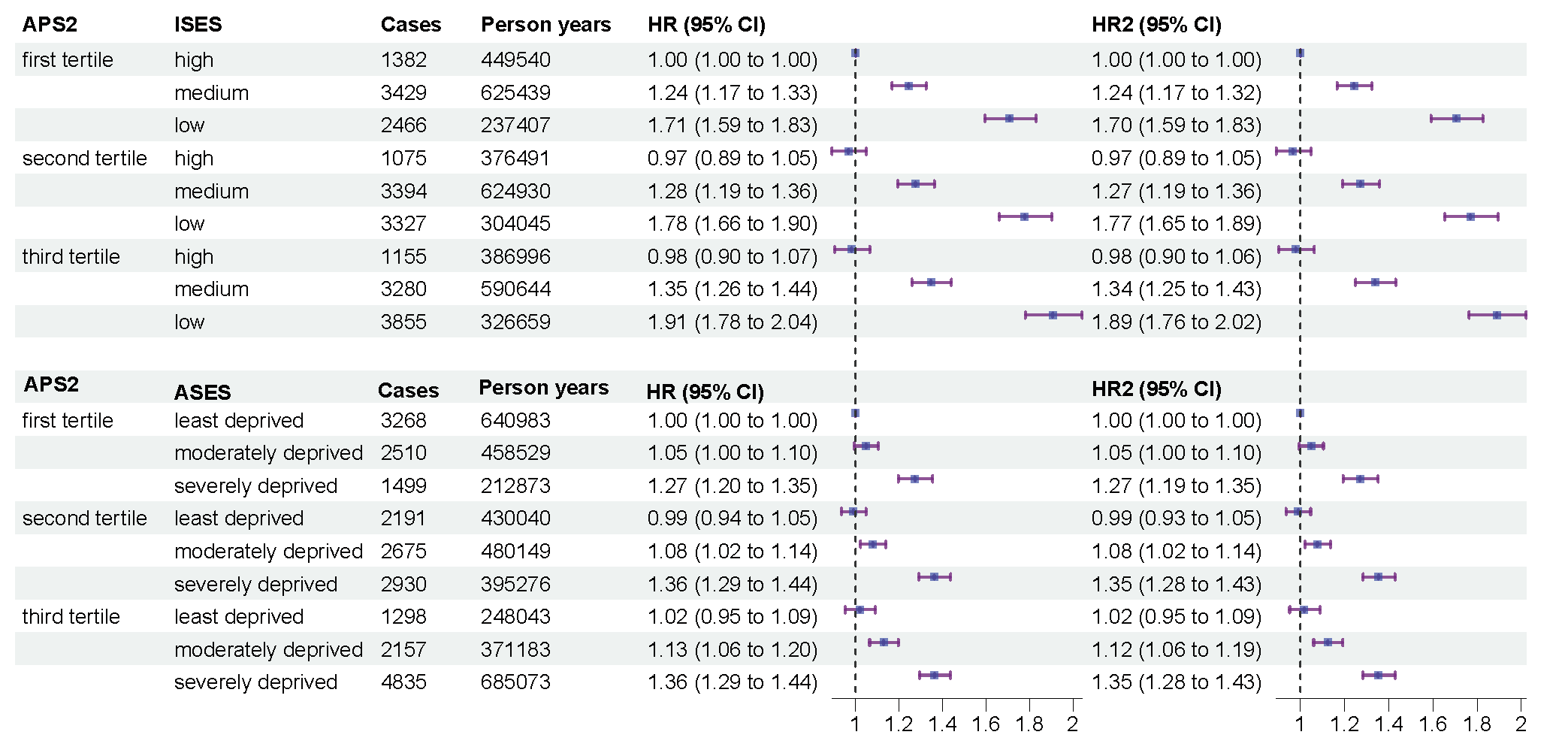
CI, confidence interval; HR, hazard ratio. APS1: air pollution score calculated by PCA; APS2: air pollution score calculated by weighted coefficients; ISES, individual-level SES; ASES, area-level SES; AMD, age-related macular degeneration. Model 1 adjusted for age at recruitment, sex, region, body mass index, ethnicity, smoking status, alcohol assumption, physical activity, healthy diet score, sleep duration, noise, green space, inverse distance to nearest major road, and length of time at current address. Model 2 further adjusted for AMD genetic risk score, genotyping array, and the first 10 principal components of ancestry.

**Fig. S9. Joint associations of APS1 and area-level SES (TDI vs. IMD) with AMD**

**
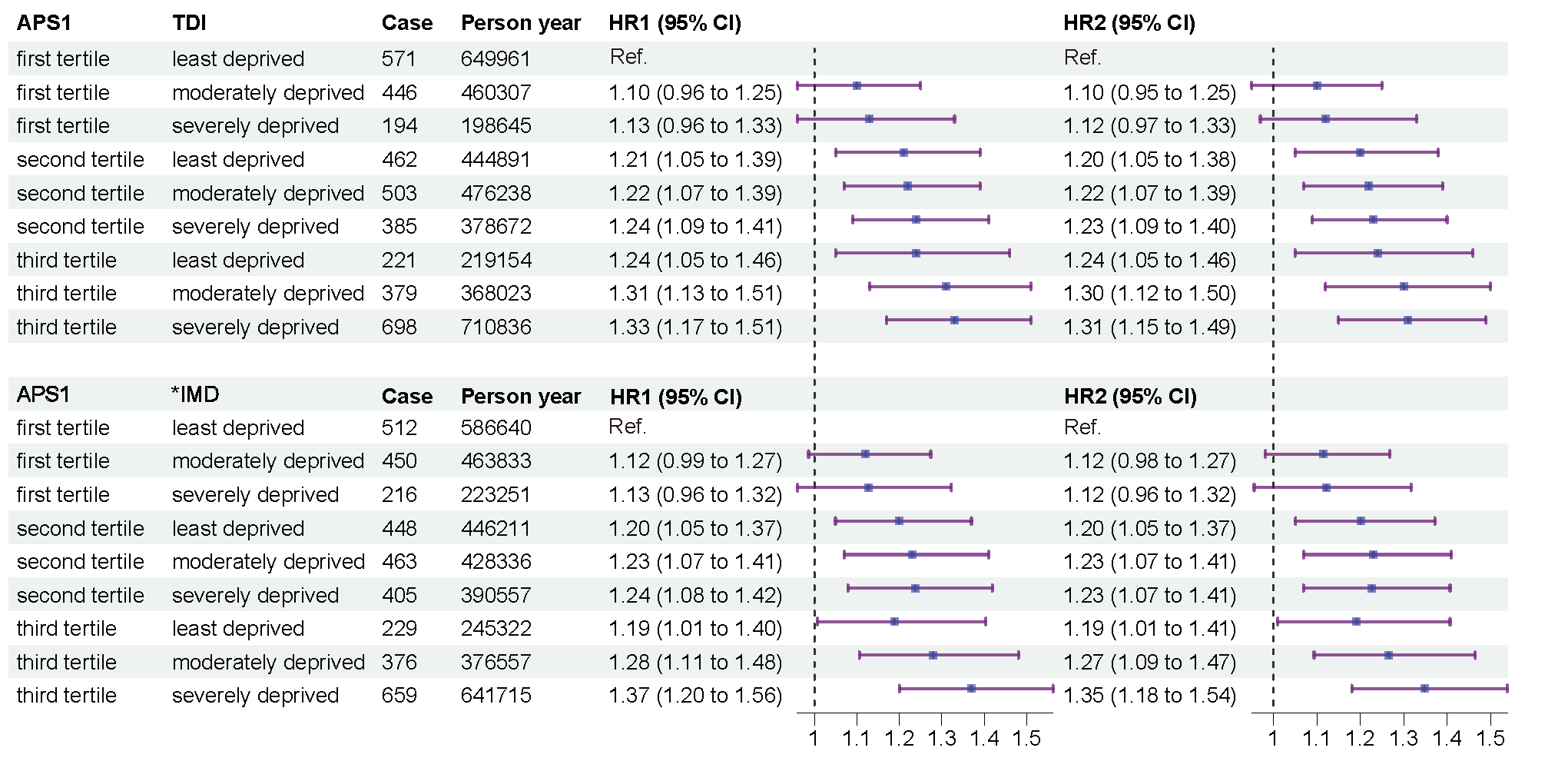
**

*N=312031. CI, confidence interval; HR, hazard ratio; APS1: air pollution score calculated by PCA; TDI, Townsend Deprivation Index; IMD, Index of Multiple Deprivation; AMD, age-related macular degeneration. Model 1 adjusted for age at recruitment, sex, region, body mass index, ethnicity, smoking status, alcohol assumption, physical activity, healthy diet score, sleep duration, noise, green space, inverse distance to nearest major road, and length of time at current address. Model 2 further adjusted for AMD genetic risk score, genotyping array, and the first 10 principal components of ancestry.

**Fig. S10. Joint associations of APS2 and area-level SES (TDI vs. IMD) with AMD**

**
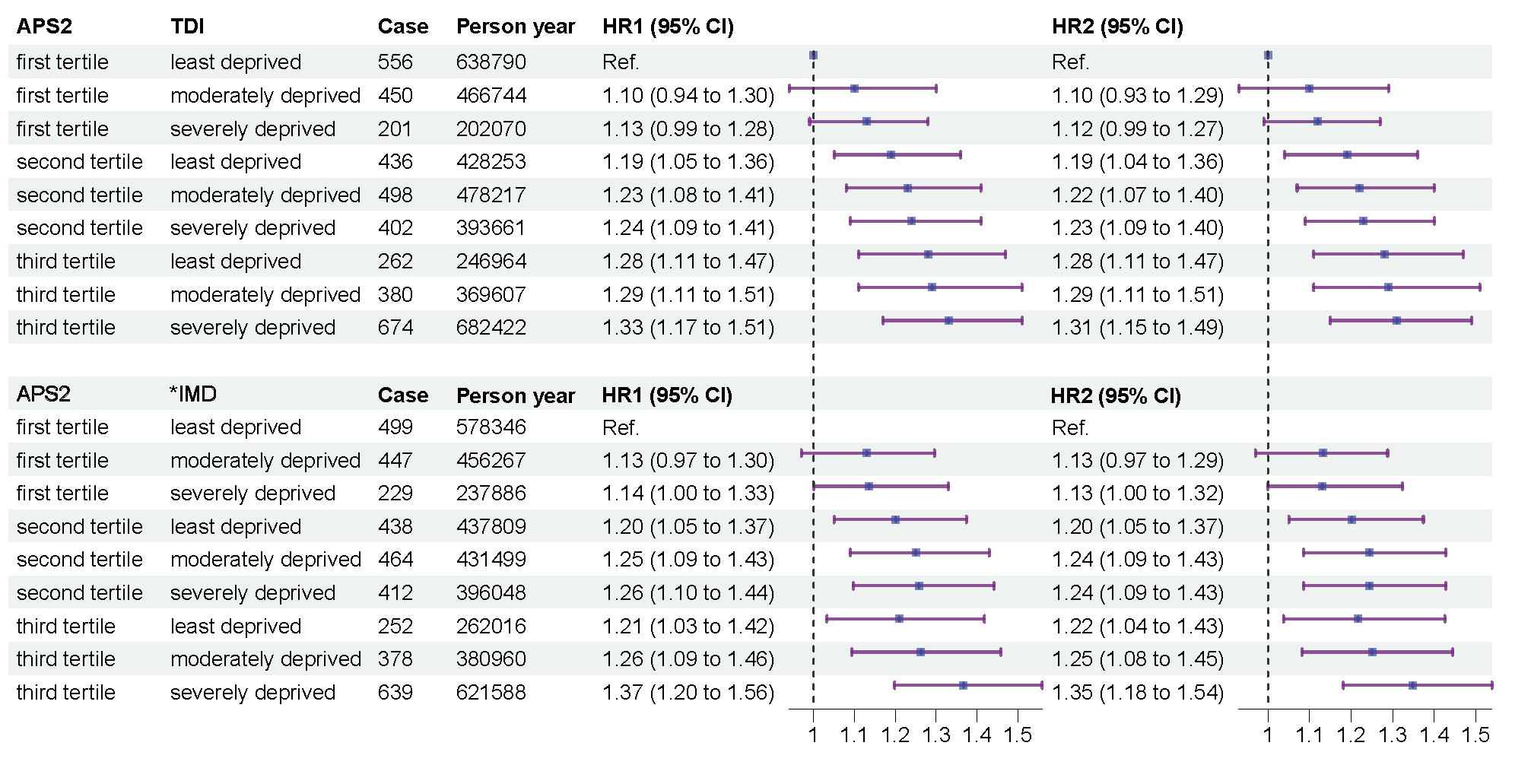
**

*N=312031. CI, confidence interval; HR, hazard ratio; APS2: air pollution score calculated by weighted coefficients; TDI, Townsend Deprivation Index; IMD, Index of Multiple Deprivation; AMD, age-related macular degeneration. Model 1 adjusted for age at recruitment, sex, region, body mass index, ethnicity, smoking status, alcohol assumption, physical activity, healthy diet score, sleep duration, noise, green space, inverse distance to nearest major road, and length of time at current address. Model 2 further adjusted for AMD genetic risk score, genotyping array, and the first 10 principal components of ancestry.

**Fig. S11. Joint associations of air pollutants and AMD-PRS with incident AMD**

**
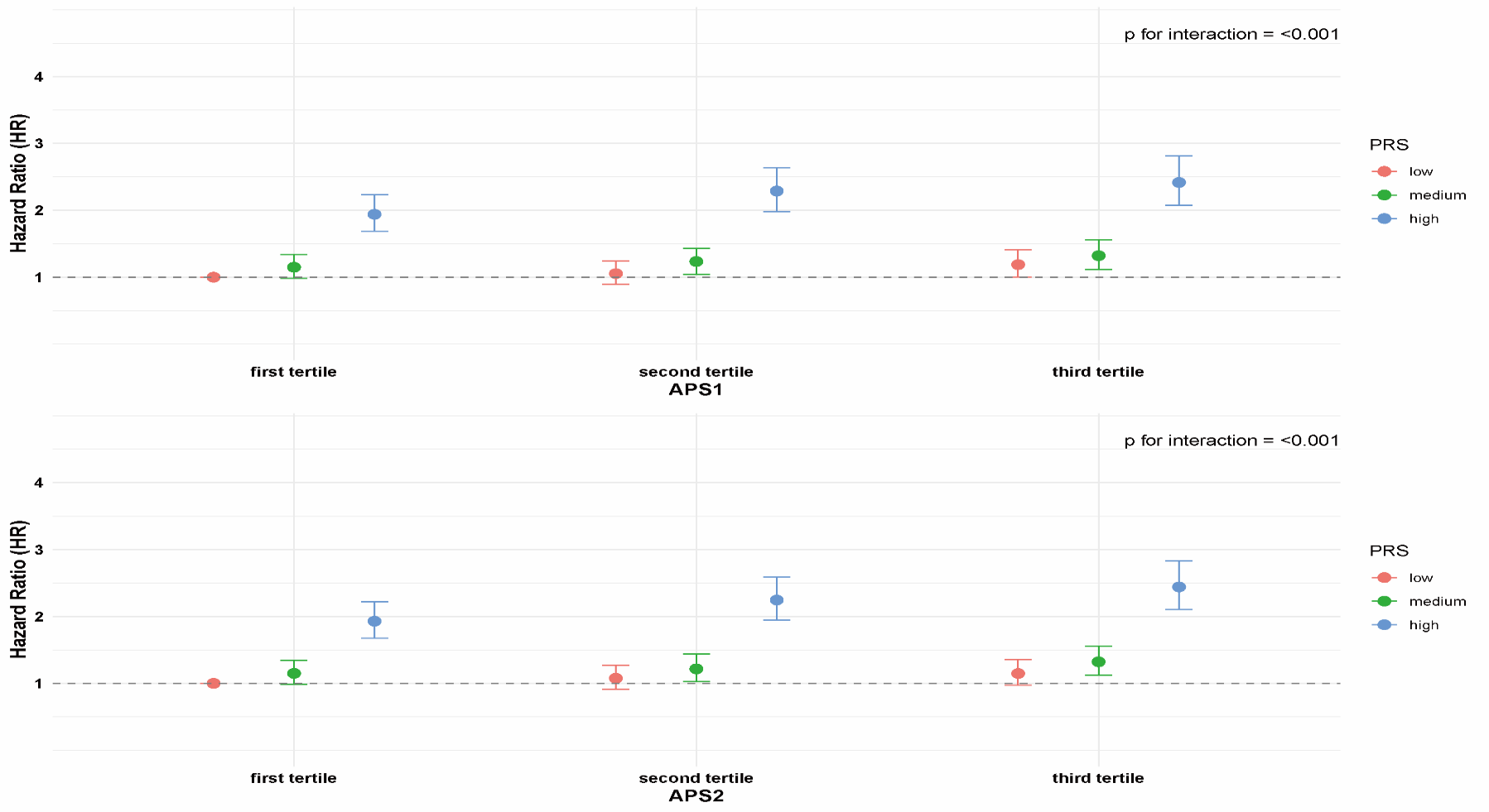
**

APS1: air pollution score calculated by PCA; APS2: air pollution score calculated by weighted coefficients; AMD, age-related macular degeneration; PRS, polygenic risk score. Models adjusted for age at recruitment, sex, region, body mass index, ethnicity, smoking status, alcohol assumption, physical activity, healthy diet score, sleep duration, noise, green space, inverse distance to nearest major road, and length of time at current address, individual-level SES and area-level SES.

**Fig. S12. Joint associations of air pollutants and healthy lifestyle with incident AMD**

**
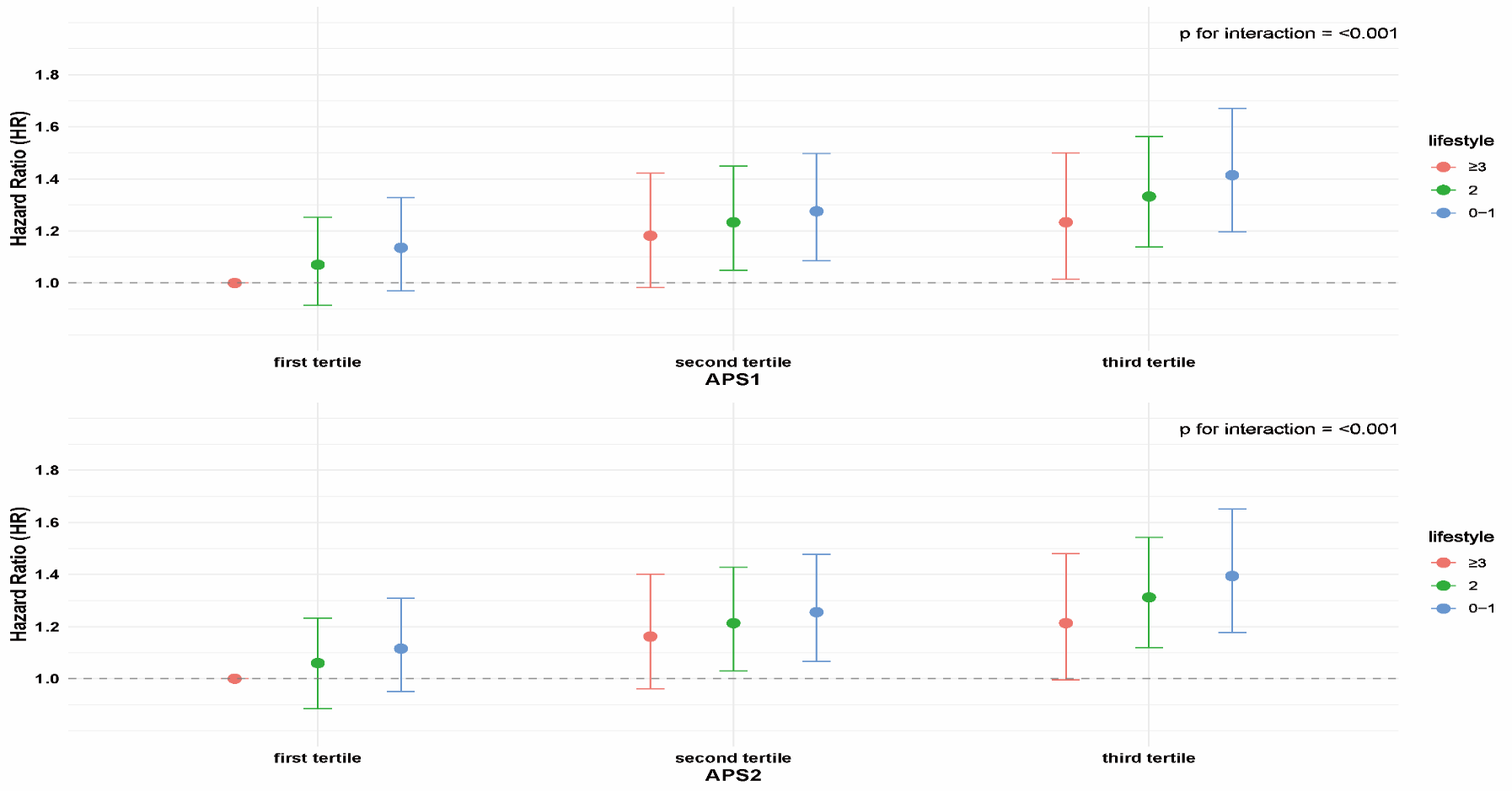
**

APS1: air pollution score calculated by PCA; APS2: air pollution score calculated by weighted coefficients; AMD, age-related macular degeneration. Models adjusted for age at recruitment, sex, region, body mass index, ethnicity, AMD genetic risk score, noise, green space, inverse distance to nearest major road, and length of time at current address, individual-level SES and area-level SES. Lifestyle score was constructed based on smoking status, alcohol consumption, physical activity, healthy diet score, and sleep duration. Each healthy behaviour was assigned a score of 1 (never smoked, never drank, high physical activity, diet score ≥6, sleep duration of 7-8 hours). The overall lifestyle score was then classified into three categories: “0-1”, “2”, and “≥3”.
